# *De Novo* Design in an M_1_ Positive Allosteric Modulator Backup Program: The Discovery of VU6069863

**DOI:** 10.1021/acsmedchemlett.6c00392

**Published:** 2026-08-21

**Authors:** Changho Han, Upendra Rathnayake, Alison R. Gregro, Julie L. Engers, Christopher C. Presley, Nathan D. Schley, Analisa D. Thompson Gray, Michael Bubser, Hyekyung P. Cho, Alice L. Rodriguez, Valerie M. Kramlinger, Olivier Boutaud, Colleen M. Niswender, Carrie K. Jones, Darren W. Engers, Craig W. Lindsley

**Affiliations:** † Warren Center for Neuroscience Drug Discovery, 5718 Vanderbilt University, Nashville, Tennessee 37232, United States; ‡ Department of Pharmacology, 12327 Vanderbilt University School of Medicine, Nashville, Tennessee 37232, United States; § Department of Chemistry, 5718 Vanderbilt University, Nashville Tennessee 37232, United States; ∥ Vanderbilt Kennedy Center, 12328 Vanderbilt University Medical Center, Nashville, Tennessee 37232, United States; ⊥ Vanderbilt Brain Institute, 5718 Vanderbilt University, Nashville, Tennessee 37232, United States; # Vanderbilt Institute of Chemical Biology, 5718 Vanderbilt University, Nashville, Tennessee 37232, United States

**Keywords:** cognition, de novo design, human dose projection, muscarinic acetylcholine receptor subtype 1 (M_1_), positive allosteric modulator (PAM)

## Abstract

Recently, we disclosed our M_1_ PAM that had advanced into Phase I clinical studies, VU0467319, as well as a deuterated back-up compound, VU6045422, to address a major metabolite produced in man and improve tox profile. Ultimately, this series failed to overcome its liabilities and was terminated. However, the team was challenged with developing chemically distinct backup candidates without performing a new HTS campaign. *De novo* design strategies based upon the other two M_1_ ago-PAMs that had previously entered clinical development, MK-7622 and TAK-071, led to the discovery of a fundamentally new chemotype based on a 3,4-dihydrobenzo­[*f*]­[1,4]­oxazepin-5-(*2H*)-one core, providing VU6069863. VU6069863 proved to be an M_1_ PAM (EC_50_ = 248 nM, 87% ACh Max) with minimal M_1_ agonism, (EC_50_ > 30 μM, 19% ACh Max) despite being derived from potent M_1_ ago-PAM chemotypes, with good CNS penetration (rat K_p_ = 0.59, K_p,uu_ = 0.13, MDCK-MDR1 ER = 1.8, P_app_ = 32.8 × 10^–6^ cm/s) and pro-cognitive efficacy in both contextual fear conditioning (minimally effective dose [MED] = 0.1 mg/kg PO) and novel object recognition (MED = 1 mg/kg PO) in rats.

## Graphical abstract

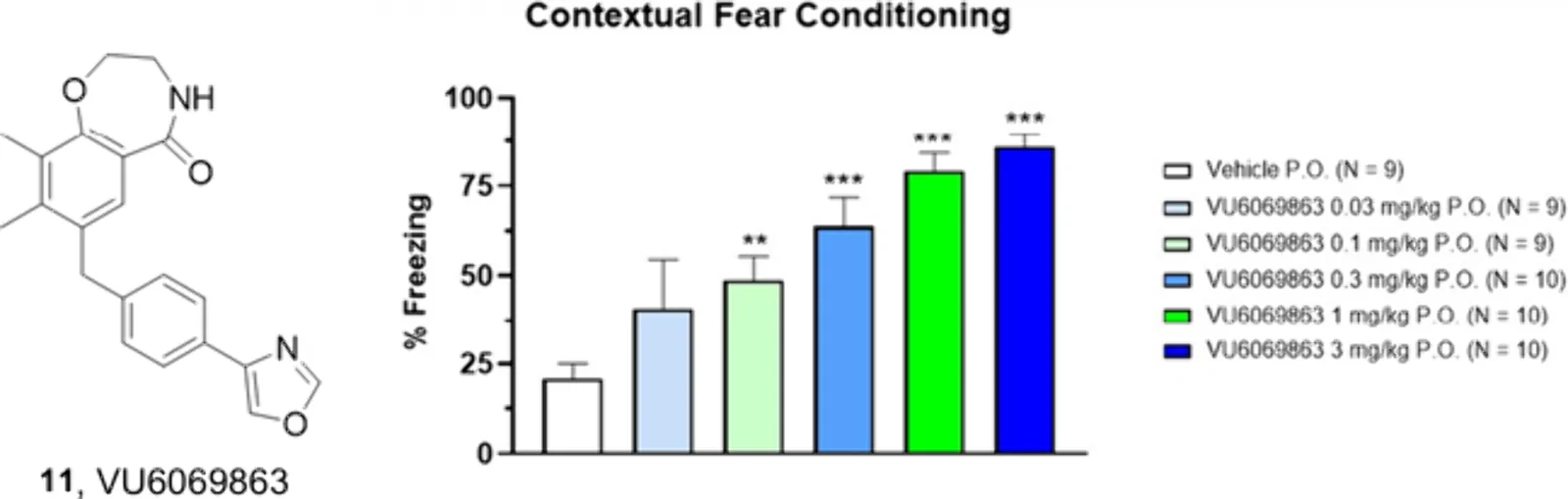

The selective activation of muscarinic acetylcholine receptors (M_1–5_), and in particular M_1_, has been a long sought-after mechanism to address the cognitive deficits in Alzheimer’s disease, schizophrenia and other neuropsychiatric disorders.^1–6^ Since the discovery that nonselective “M_1_ agonists” exhibited robust efficacy in improving cognition in Alzheimer’s patients in the 1980s, the focus was on muscarinic subtype selectivity to avoid activation of peripheral M_2_ and M_3_ receptors and the undesired SLUDGE reaction (salivation, lacrimation, urination, defecation, gastrointestinal distress and emesis).^1–6^ Toward this goal, we have been very active in the field of M_1_ positive allosteric modulators (PAMs), where exquisite subtype selectivity for M_1_ is achieved by virtue of binding to an allosteric site and potentiating the agonist effects of endogenous acetylcholine.^7–20^ Both preclinical *in vivo* tool M_1_ PAM compounds (with minimal M_1_ agonism) from our lab and the clinical compound VU0467319 (**1**) have demonstrated pro-cognitive efficacy and a lack of cholinergic and SLUDGE side effects in preclinical species (mouse, rat, dog, cynomolgus monkey) and in humans (Figure 1).^21^ However, a major inactive circulating metabolite, VU0481424 (**2**), accounted for ∼50% of the total drug-related AUC upon increasing the dose of **1** in the Phase I single ascending dose (SAD), coupled with tox findings in 13-week rat and development was stopped.^22–24^ The incorporation of deuterium into the core of **1**, as with VU6045422 (**3**) to diminish the production of **2,** was effective *in vitro* (e.g., microsome and hepatocyte incubations) as well as *in vivo* at low doses; however, upon dose escalation in rat and dog toxicology studies, the degree of oxidative metabolism of **3** was comparable to **1**, and all efforts around this chemotype were terminated.^25^ In need of a chemically distinct M_1_ PAM backup, we recently disclosed VU6007496 (**4**),^26^ a potent M_1_ PAM with minimal agonism; yet, disconnect between *in vitro* and *in vivo* metabolite identification, and the production of a potent M_1_ ago-PAM via oxidative metabolism that engendered cholinergic adverse events (AEs), led to the termination of this scaffold. In this paper, we will detail our efforts for the *de novo* design of a unique M_1_ PAM chemotype based on a 3,4-dihydrobenzo­[*f*]­[1,4]­oxazepin-5-(*2H*)-one core that displayed minimal M_1_ agonism and robust efficacy in rodent cognition models.

**Figure 1:**
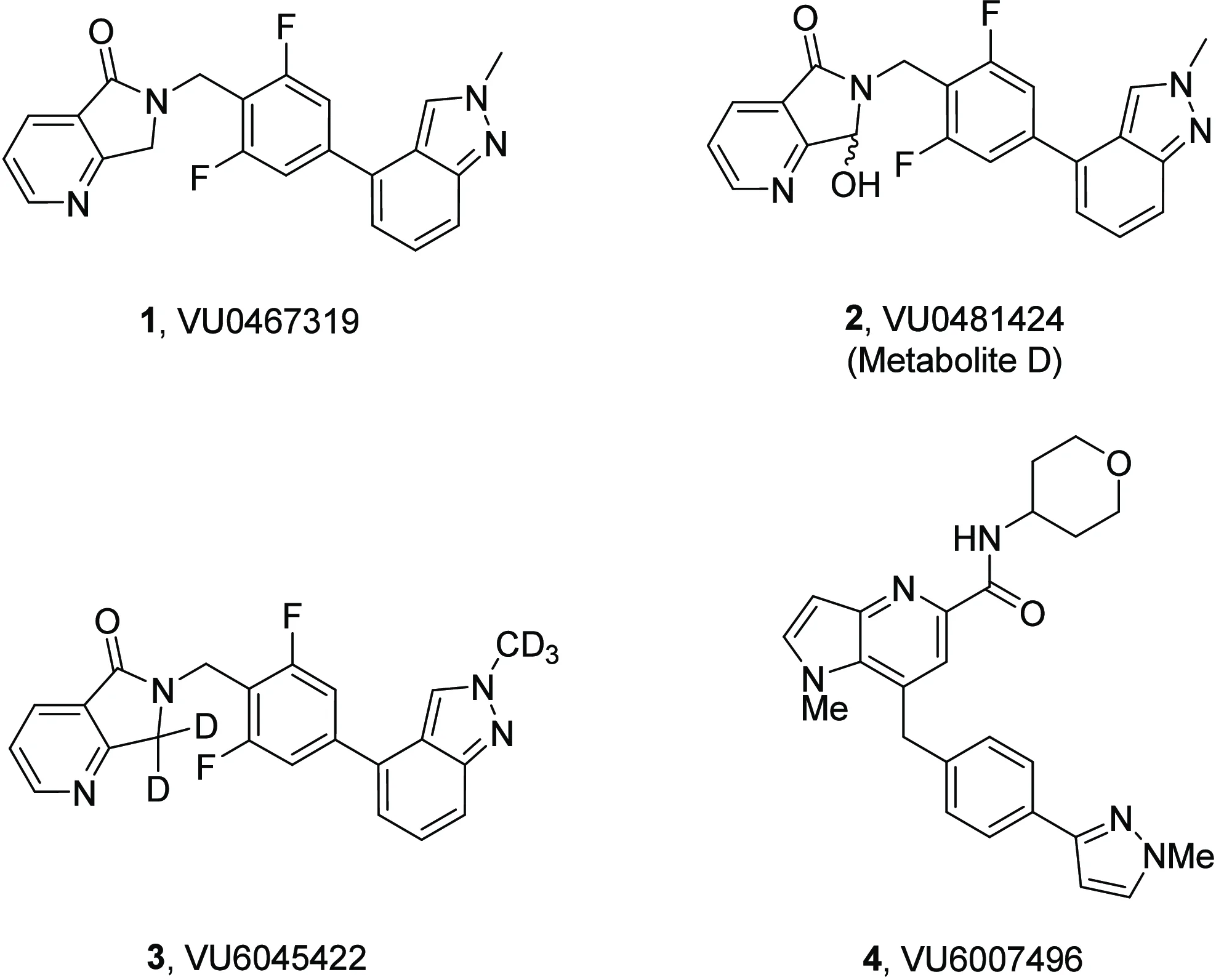
Structures of the clinical M_1_ PAM VU0467319 **1**, and the corresponding oxidative metabolite **2**, that accounted for ∼50% of the total plasma AUC in man. A penta-deutero congener **3** (VU6045422) was developed to address the undesired metabolism, which was effective *in vitro* and at low doses *in vivo*, but, upon dose escalation in rats and dogs was indistinguishable from **1** in terms of metabolite production. A distinct M_1_ PAM chemotype VU6007496 (**4**) was plagued with *in vitro:in vivo* metabolism disconnects and the production of an M_1_ ago-PAM metabolite affording cholinergic AEs.

Having exhausted our internal M_1_ PAM high-throughput screening hits, we turned to scaffold-hopping of the known M_1_ ago-PAMs that had advanced into the clinic. For this exercise, we employed Merck’s MK-7622 (**5**)^13^ and Takeda’s TAK-071 (**6**)^20^ as a basis for ligand design; however, this required dialing out the potent M_1_ agonist activity in **5** and **6** to generate an advanceable chemotype (Figure 2).^14^ It was known that the exocyclic cyclohexyl alcohol moiety of **5** and the exocyclic pyranyl alcohol moiety of **6** were significant contributors to the undesired M_1_ agonist activity; therefore, we deleted these in our design.^7–20,26^ However, we thought the design should be cautious because previously reported SAR trends in the BQCA scaffold (**7**) suggested that the exocyclic cyclohexyl alcohol moiety has a significant role in activity upon comparing **8** and **9**.^13^

**Figure 2:**
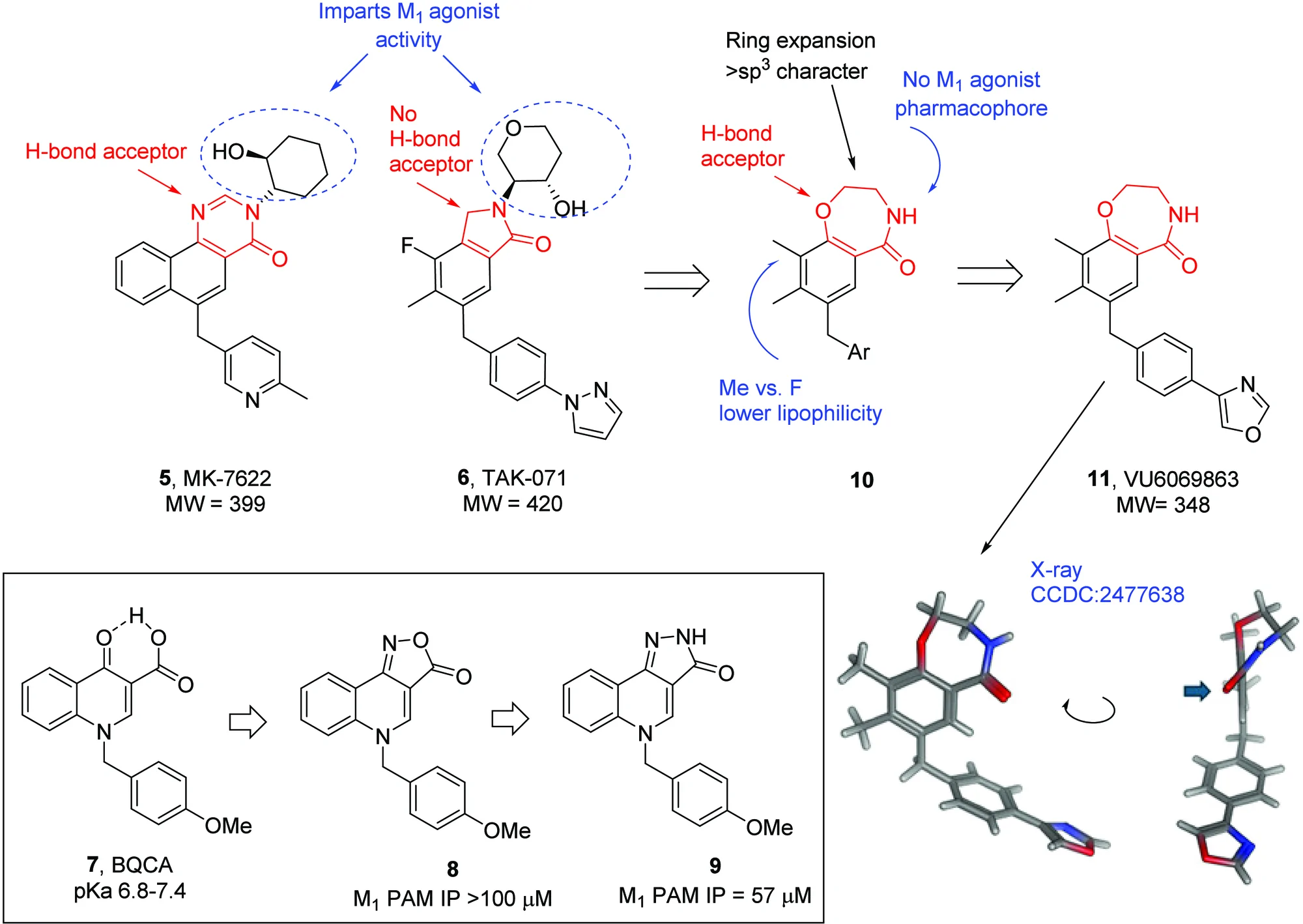
*De novo* design of a novel 3,4-dihydrobenzo­[*f*]­[1,4]­oxazepin-5-(*2H*)-one based M_1_ PAM (**11**) with minimal agonism, derived from the potent M_1_ ago-PAMs **5** and **6** that had advanced into clinical development, and SAR trend within the BQCA scaffold **6**–**8**. CCDC#: 2477638.

Ago-PAM **5** possesses an additional hydrogen bond acceptor in the pyrimidine ring that is absent in ago-PAM **6**; moreover, escaping “flat-land” and increasing sp^3^ character as with **6** were attractive. Thus, we incorporated an oxygen atom as a hydrogen bond acceptor into **6**, and expanded the γ-lactam of **6** into a 7-membered ring to afford a novel 3,4-dihydrobenzo­[*f*]­[1,4]­oxazepin-5-(*2H*)-one core **9**. Optimization of the southern “tail” group led to the identification of 4-oxazole phenyl heterobiaryl moiety as a preferred group, and the discovery of VU6069863 (**11**), a low molecular weight M_1_ PAM with minimal M_1_ agonism, derived, surprisingly, from potent M_1_ ago-PAMs. As can be seen in the X-ray crystal structure (Figure 2), the carbonyl of the 7-membered ring was distorted and successfully replaced the hydroxy moiety of exocyclic cyclohexanol. To our best knowledge, VU6069863 (**11**) is the first example showing the importance of the position and angle of the hydrogen bond acceptor, considering that several compounds synthesized for comparison were inactive or very weakly active in which the carbonyl was coplanar with the core.

A convergent synthetic approach was developed for the gram quantity production of M_1_ PAM **11** (Scheme 1) to enable full *in vitro* and *in vivo* characterization. Esterification of commercial benzoic acid **12** provides **13** in 93% yield. Bromination proceeded with excellent regio-chemistry to deliver **14** in 98% yield. A Mitsunobu reaction with *N*-Boc aminoethanol installs the tether for the 7-membered ring **15**, followed by removal of the Boc group to give **16**, and base-promoted lactam formation provides the 3,4-dihydrobenzo­[*f*]­[1,4]­oxazepin-5-(*2H*)-one core **17** in 76% yield for the three steps. A palladium catalyzed borylation of **17** with bis­(pinacolato)­diboron generates Suzuki coupling partner **18** in 88% yield. In parallel, the southern “tail” was prepared from commercial acetyl bromide **19**. Standard oxazole formation proceeded smoothly giving **20** in 32% yield, followed by a palladium-catalyzed carbonylation to provide ester **21** in 88% yield. Reduction to the benzyl alcohol with LAH proceeded in high yield, and subsequent chlorination afforded the other Suzuki coupling partner **23**. Finally, a Suzuki coupling between **18** and **23** delivered VU6069863 (**11**) in 82% yield. Overall, the convergent preparation of **11** required 11 steps (7 steps longest linear sequence) in 8.7% overall yield on a 10-g scale.

**Scheme 1:**
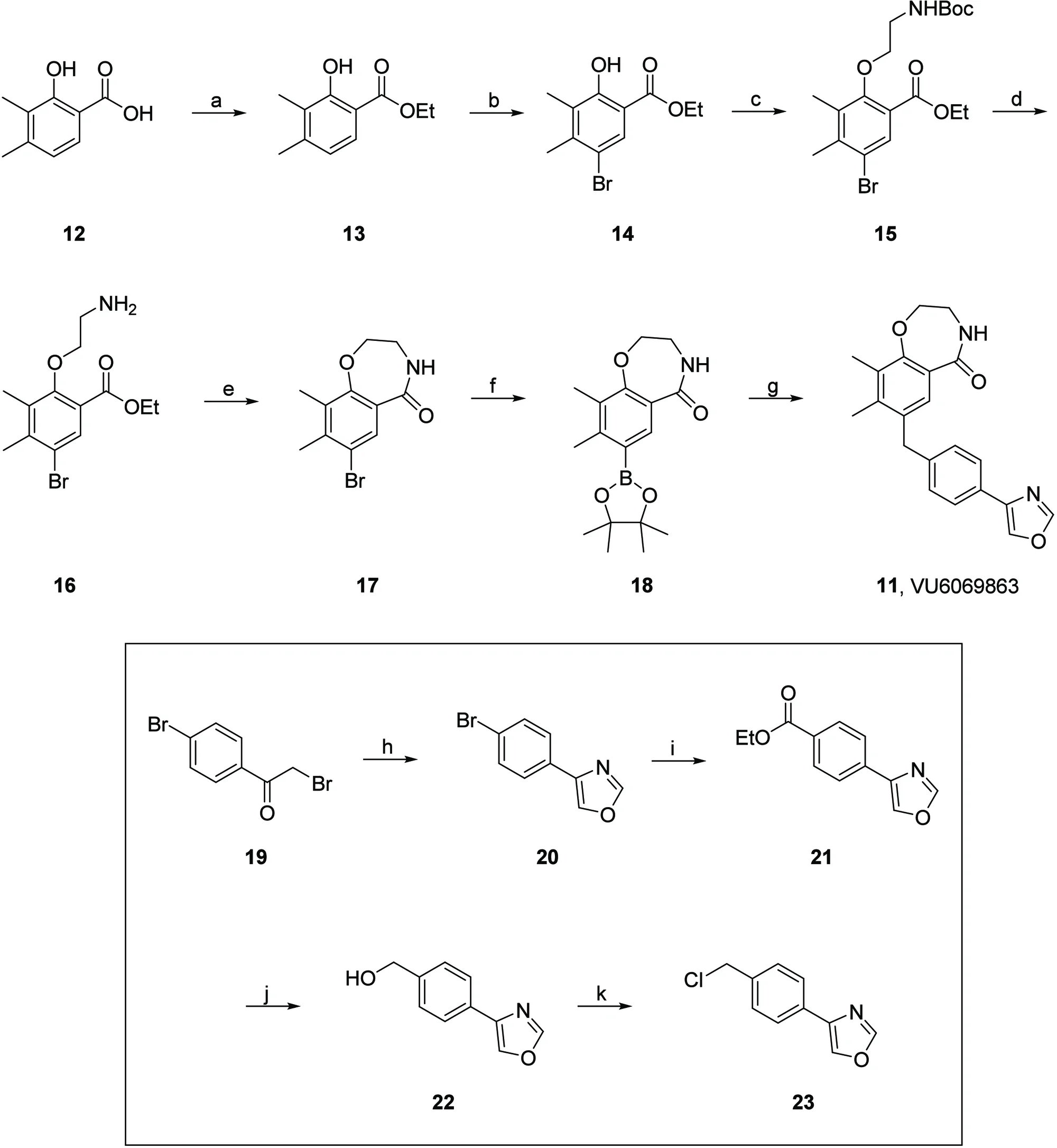
Synthesis of VU6069863 (**11**)­^a^

a Reagents and conditions: (a) EtOH, H_2_SO_4_, 100 °C, 16 h, 93%; (b) Br_2_, CHCl_3_, 0 °C, 98%; (c) HOCH_2_CH_2_NHBoc, PPh_3_, DIAD, THF, rt; (d) 4 N HCl in dioxane, rt; (e) K_2_CO_3_, EtOH, 80 °C, 18 h, 76% over three steps; (f) bis­(pinacolato)­diboron, KOAc, Pd­(dppf)­Cl_2_, 1,4-dioxane, 90 °C, 88%; (g) **23**, Cs_2_CO_3_, Pd­(dppf)­Cl_2_, 1,4-dioxane/H_2_O, 100 °C, 80%; (h) HCO_2_NH_2_, HCOOH, 115 °C, 2.5 h, 32%; (i) CO (g), Et_3_N, Pd­(OAc)_2_, dppp, EtOH/DMSO, 75 °C, 88%; (j) 2 M LiAlH_4_, THF, 0 °C, 83%; (k) SOCl_2_, DCM, rt, 97%.

As the basis for the design was known for M_1_ ago-PAMs,^13,20^ we hypothesized that our design strategy would mitigate and/or minimize the degree of M_1_ agonism in **11** to avoid overstimulation of the M_1_ receptor and resultant cholinergic AEs. Gratifyingly, **11** was found to be a moderately potent M_1_ PAM (human EC_50_ = 226 nM, 80% ACh max) with minimal M_1_ agonism (human EC_50_ > 30,000 nM, 19% ACh max, an ∼121-fold margin), and thus possessed the desired profile for advancement (Table 1). At rat M_1_, a similar pharmacological profile was noted, with compound activity observed as a potent M_1_ PAM (rat EC_50_ = 140 nM, 78% ACh max) with minimal M_1_ agonism (rat EC_50_ > 30,000 nM, 19% ACh max, an ∼210-fold margin). Relative to human M_1_, **11** was an ∼3-fold weaker PAM at both mouse (EC_50_ = 945 nM, 81% ACh max) and dog (EC_50_ = 895 nM, 62% ACh max) receptors, but also with minimal M_1_ agonism (EC_50_ > 30,000 nM, 20% ACh max). Additionally, PAM **11** was highly selective for M_1_ (inactive on human and rat M_2–5_) as well as dog M_2_ and M_4_ (dog M_3_ and M_5_ not tested). In terms of *in vitro* DMPK, PAM **11** displayed low predicted hepatic clearance in human (CL_hep_ = 4.8 mL/min/kg), moderate in mouse, dog and cyno (CL_hep_ = 39.4, 22.4, and 28.9 mL/min/kg, respectively) and high in rat (CL_hep_ = 57.2 mL/min/kg) microsomes. A favorable profile was also noted in terms of unbound fraction in plasma (*f*_u_ (m, r, d, c, h): 0.039, 0.066, 0.032, 0.013 and 0.039) and in rat brain homogenate binding (*f*_u_ = 0.014), as well as with regards to CYP_450_ inhibition (IC_50_ > 30 μM for 3A4, 2D6, 1A2 and 22.2 μM for 2C9). There was weak time-dependent inhibition of CYP3A and CYP2C8 along with weak induction of CYP3A4; however, these weak signals were not showstoppers for PAM **11**. Moreover, PAM **11** was predicted to be brain penetrant in human (MDCK-MDR1 ER = 1.8, P_app_ = 32.8 × 10^–6^ cm/s). A key issue to improve upon M_1_ PAMs **1** and **2** was solubility. In our kinetic solubility assay, PAM **11** showed high solubility at both neutral pH (79.7 μM at pH = 6.8) and acidic pH (77.9 μM at pH = 2.2), providing a significant improvement over **1** and **2**. Furthermore, PAM **11** displayed good solubility in FaSSIF (12.4 μg/mL) and FaSSGF (16.1 μg/mL) in addition to a sharp, low melting point (180–181.8 °C), suggesting good developability.

**Table 1:**
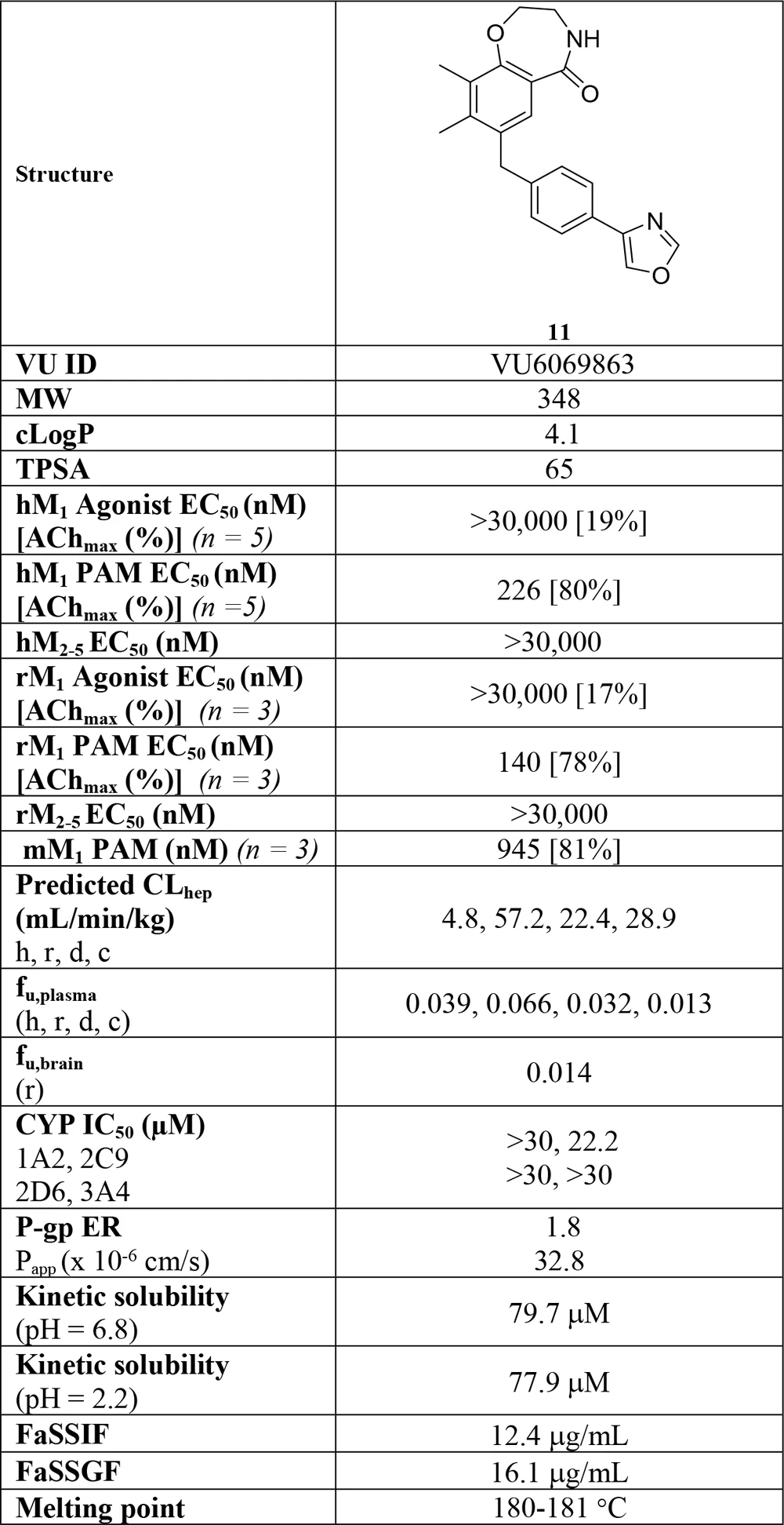
Pharmacology and *in Vitro* DMPK Profile of M_1_ PAM **11**

We performed electrophysiology studies in mouse native tissue layer V medial prefrontal cortex (mPFC) to ensure that **11** did not induce long-term depression (LTD) akin to the M_1_ ago-PAMs **5** and **6**.^14,16,21^ PAM **11** induced no significant changes in field excitatory postsynaptic potentials (fEPSPs) evoked by electrical stimulation in layer II/III at 10 μM concentration (see Supporting Information), ∼11× above the functional mouse EC_50_, and a projected 40× for human. Thus, in agreement with the minimal agonism in M_1_ cell lines, PAM **11** maintained activity dependence on PFC regulation.

To confirm a lack of central cholinergic AEs, PAM **11** was dosed by intraperitoneal (I.P.) injection at 100 mg/kg in mice to assess seizure liability using the Racine scale in comparison with a 100 mg/kg I.P. dose of the positive control, the potent M_1_ ago-PAM BQCA (**7**) (Figure 3). Across the 6-h time course of the study, no seizure activity was induced by PAM **11** (achieving total brain concentrations of 113 μM, or 120-fold the mouse M_1_ EC_50_ at 60 min). In contrast BQCA, at the same dose, rapidly induced seizure activity that peaked at Racine scale scores between 3 to 4 for the duration of the study. Combined with the lack of effect on LTD, these data encouraged further progression of PAM **11**.

**Figure 3:**
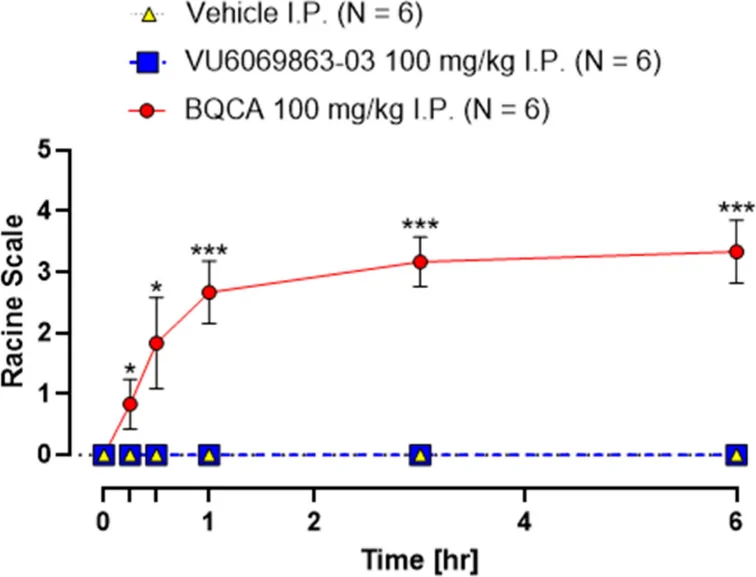
Seizure liability of M_1_ PAM **11** compared to the M_1_ ago-PAM BQCA in male C57BL/6 mice. *p < 0.05; ***p < 0.001 vs. Vehicle (two-way ANOVA followed by Šídák’s multiple comparisons test). Vehicle for M_1_ PAM **11**: aqueous 10% Tween 80; Vehicle for BQCA: aqueous 20% hydroxy-propyl-β-cyclodextrin.

In our standard rat plasma:brain level (PBL) cassette,^21^ PAM **11** was CNS penetrant, with a K_p_ of 0.59 and a K_p,uu_ of 0.13 (at a 15 min time point), and in agreement with the MDCK-MDR1 efflux data. *In vivo* IV/PO PK in rat was favorable (CL_p_ = 9.1 mL/min/kg, *t*_1/2_= 0.92 h, V_ss_ = 0.61 L/kg, %F = 100) and a clear IVIVC disconnect with the high rat predicted hepatic clearance. Dog *in vivo* pharmacokinetics was also better than predicted (CL_p_ = 11.5 mL/min/kg, *t*_1/2_= 1.4 h, V_ss_ = 0.97 L/kg, %F = 100), with high oral bioavailability in both of our presumed toxicology species. In a cyno IV PK cassette study (0.2 mg/kg), a similar favorable profile was obtained (CL_p_ = 13.5 mL/min/kg, *t*_1/2_= 0.67 h, V_ss_ = 0.63 L/kg). Prior to advancing PAM **11** into rat cognition models, we evaluated the broader ancillary pharmacology in a CEREP 44 safety screen radioligand displacement at 10 μM PAM **11**). For 42/44 targets, there was no displacement >50%@10 μM; however, in the single point screen, **11** displaced both the 5-HT_2A_ (82.2%) and 5-HT_2B_ (95.7%) agonist radioligands. In follow-up 10-point CRC assays, both proved to be >10 μM, and thus was clear to advance. Transitioning to cardiovascular safety, PAM **11** was clean on hERG (manual patch clamp IC_50_ > 30 μM) and in the broader CV EP panel (all >10 μM, except KV_1.3_ IC_50_ = 6.2 μM). Finally, PAM **11** was negative in a mini-AMES assay (4-strain, ±S9).

Our M_1_ PAM program had historically been driven by a rat novel object recognition (NOR) assay (Figure 4),^21^ a preclinical model of prefrontal cortical memory functions. We therefore started our pharmacodynamic characterization of PAM **11** in the NOR; and observed a dose-dependent enhancement in recognition index, consistent with earlier M_1_ PAMs evaluated in this assay.^21^ To assess if PAM **11** also facilitates hippocampal memory processes, we next examined its effects on the acquisition of contextual fear conditioning (CFC) in rats. Compared to NOR, the dose–response in CFC was leftward-shifted with efficacy observed at lower doses of PAM **11** (Figure 5).^27^ The MED in CFC was 0.1 mg/kg PO, which corresponded to 90 nM total brain levels or 0.62-fold the rat EC_50_. This PK/PD relationship is consistent with previously observed effects in the history of our M_1_ PAM program, where total rather than free brain compound exposure drives PD.^21,25,26^

**Figure 4:**
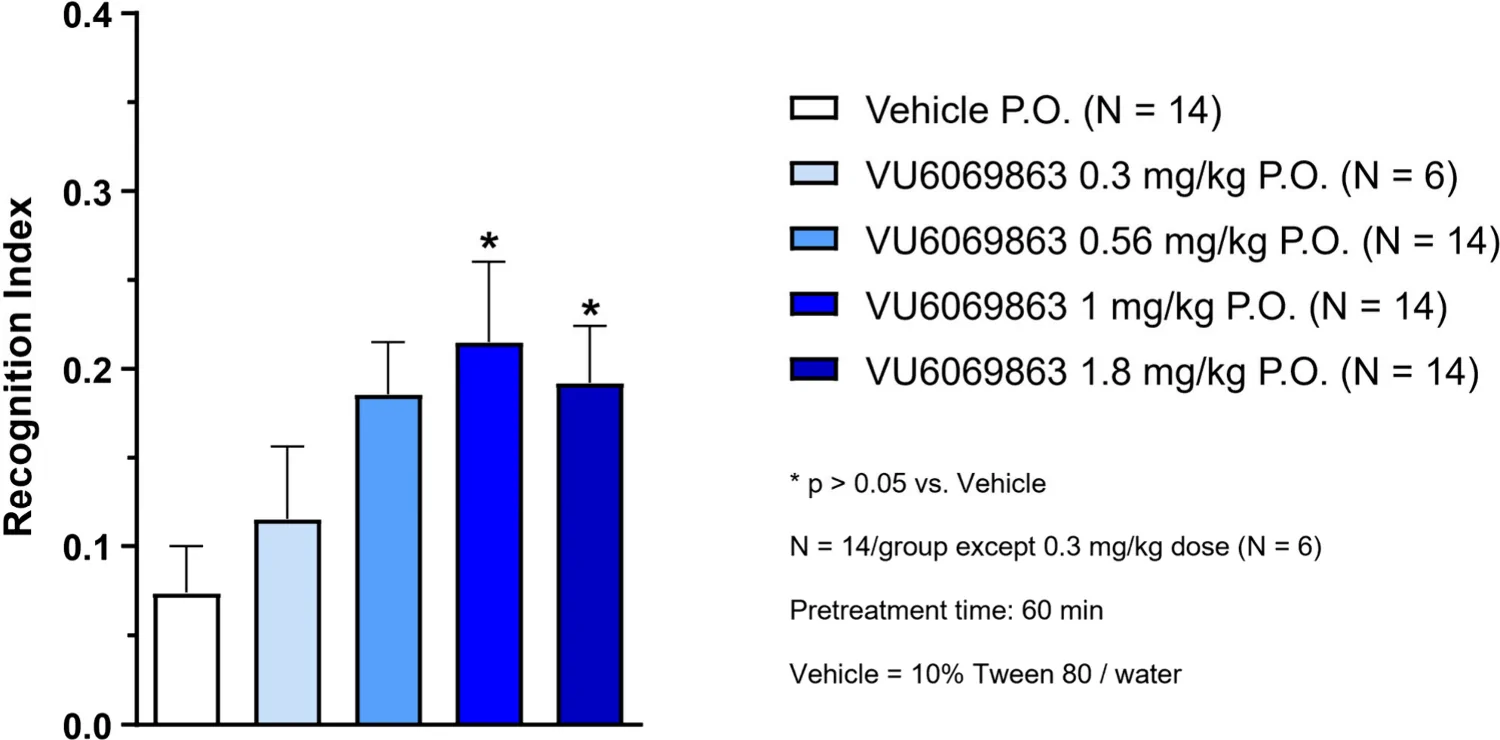
Effects of M_1_ PAM **11** in the Novel Object Recognition (NOR) test in rats. PAM **11** dose-dependently increased the Recognition Index in male Sprague–Dawley rats. Pretreatment by oral gavage with PAM **11** (0.3, 0.56, 1, and 1.8 mg/kg in aqueous 10% Tween 80 vehicle) 60 min prior to exposure to two identical objects significantly enhanced recognition memory assessed 24 h later. *p ≤ 0.05 vs. Vehicle (one-way ANOVA followed by Dunnett’s *posthoc* test).

**Figure 5:**
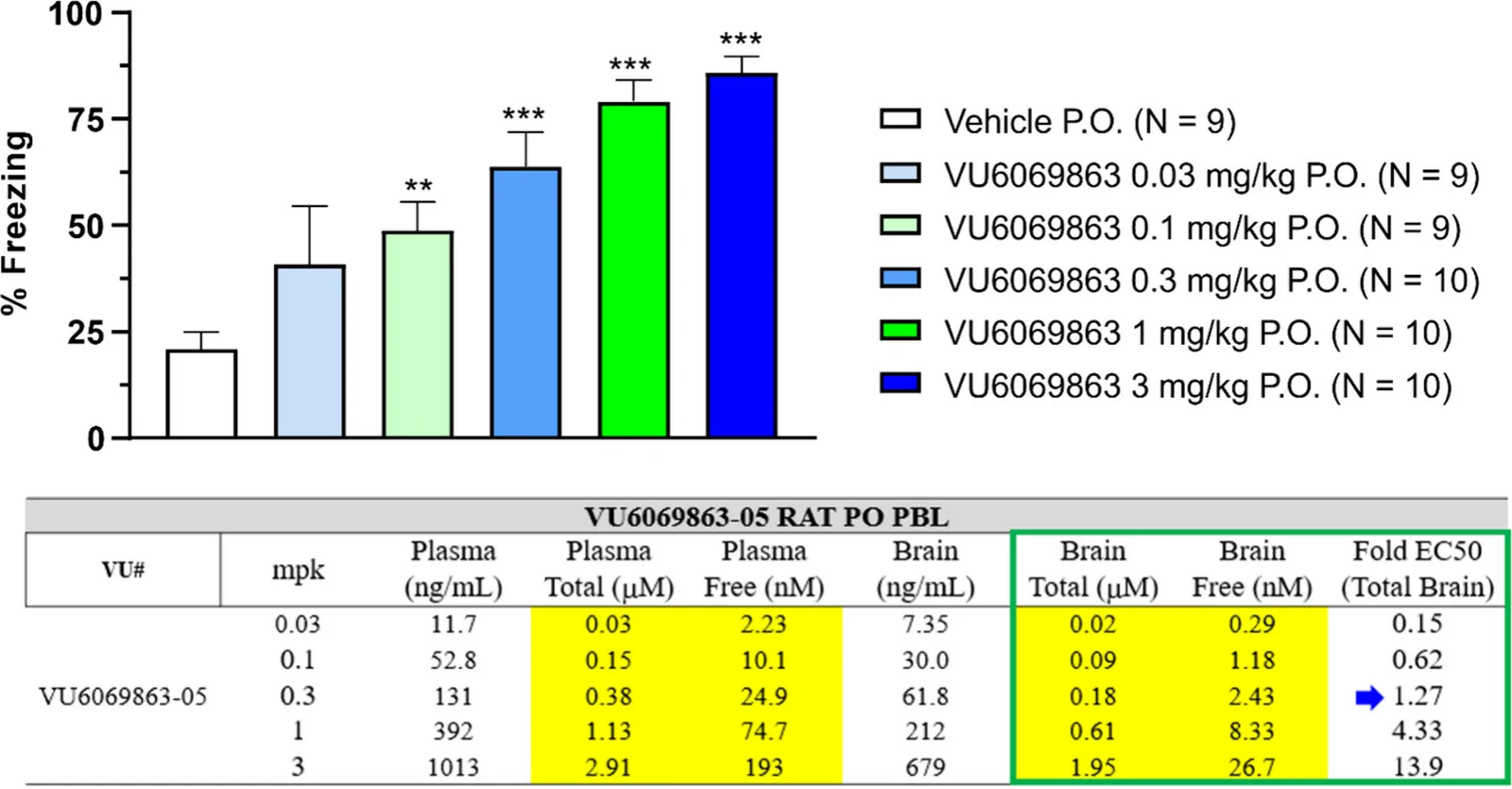
Effects of M_1_ PAM **11** on the acquisition of Contextual Fear Conditioning (CFC) in rats. On training day, pretreatment by oral gavage with the PAM **11** (dose range: 0.03, 0.1, 0.3, 1, and 3 mg/kg in aqueous 10% Tween 80 vehicle) 60 min prior to exposure to foot shock stimulus in rats, dose-dependently enhanced the precent of context-dependent freezing behavior 24 h later. *N* = 9–10/group of male Sprague–Dawley rats. **p < 0.01; ***p < 0.001 vs. Vehicle (one-way ANOVA followed by Dunnett’s posthoc test).

As discussed previously, this is likely due to the fact that we arbitrarily determine the PAM EC_50_ potency employing an EC_20_ concentration of ACh. However, in healthy young rats, cortical and hippocampal cholinergic tone may be higher, which would be predicted to increase PAM functional potency.^21,25,26^ Consistently across dozens of M_1_ PAM chemotypes, when total brain exposure approaches or meets the M_1_ PAM EC_50_, significant pharmacodynamic effects in rodent cognition models were observed. With no observed cholinergic AEs and robust efficacy in both NOR and CFC, PAM **11** was further profiled as a putative backup compound for VU0467319 (**1**).

To select the second toxicology species and to ensure there were no unique human metabolites, multispecies (rat, dog, monkey, human) hepatocyte incubations were conducted (Figure 6). Across all species, PAM **11** was the major species, with more extensive metabolism in rat and monkey. However, there was coverage across species (e.g., all metabolites were detected in all species), no unique human metabolites were detected, and dog was suitable as the second toxicology species. From our work with PAM **4**, we knew there was potential to form active metabolites as either M_1_ PAMs or undesired ago-PAMs.^21,25,26^ Thus, the team elected to synthesize (see Supporting Information for full synthetic details) the most abundant metabolites identified: the two regioisomeric benzyl alcohols M364a (**24**) and M364b (**25**), the ring-opened oxepinone M380a (**26**), and the α-hydroxy acid M355 (**27**) resulting from metabolism of the oxazole. In general (Table 2), the putative metabolites were weak to inactive M_1_ PAMs with minimal to no detectable M_1_ agonism. Interestingly, regioisomeric benzyl alcohol M364a (**24**) was more potent than M364b (**25**), and was reasonably active at rat M_1_ (EC_50_ = 248 nM, 84%), but weak at human M_1_ (EC_50_ = 1,000 nM, 66%). Fortunately, all four metabolites showed poor brain penetration in rat (K_p_s ∼ 0.05, K_p,uu_s ∼ 0.06). Unexpectedly, both **24** (CLp = 9.1 mL/min/kg, *t*_1/2_ = 6.1 h, V_ss_ = 2.3 L/kg) and **25** (CLp = 7.9 mL/min/kg, *t*_1/2_ = 7.4 h, V_ss_ = 2.3 L/kg) had longer *t*_1/2_ *in vivo* than **11**, which led to the concern of peripheral metabolite accumulation.

**Figure 6:**
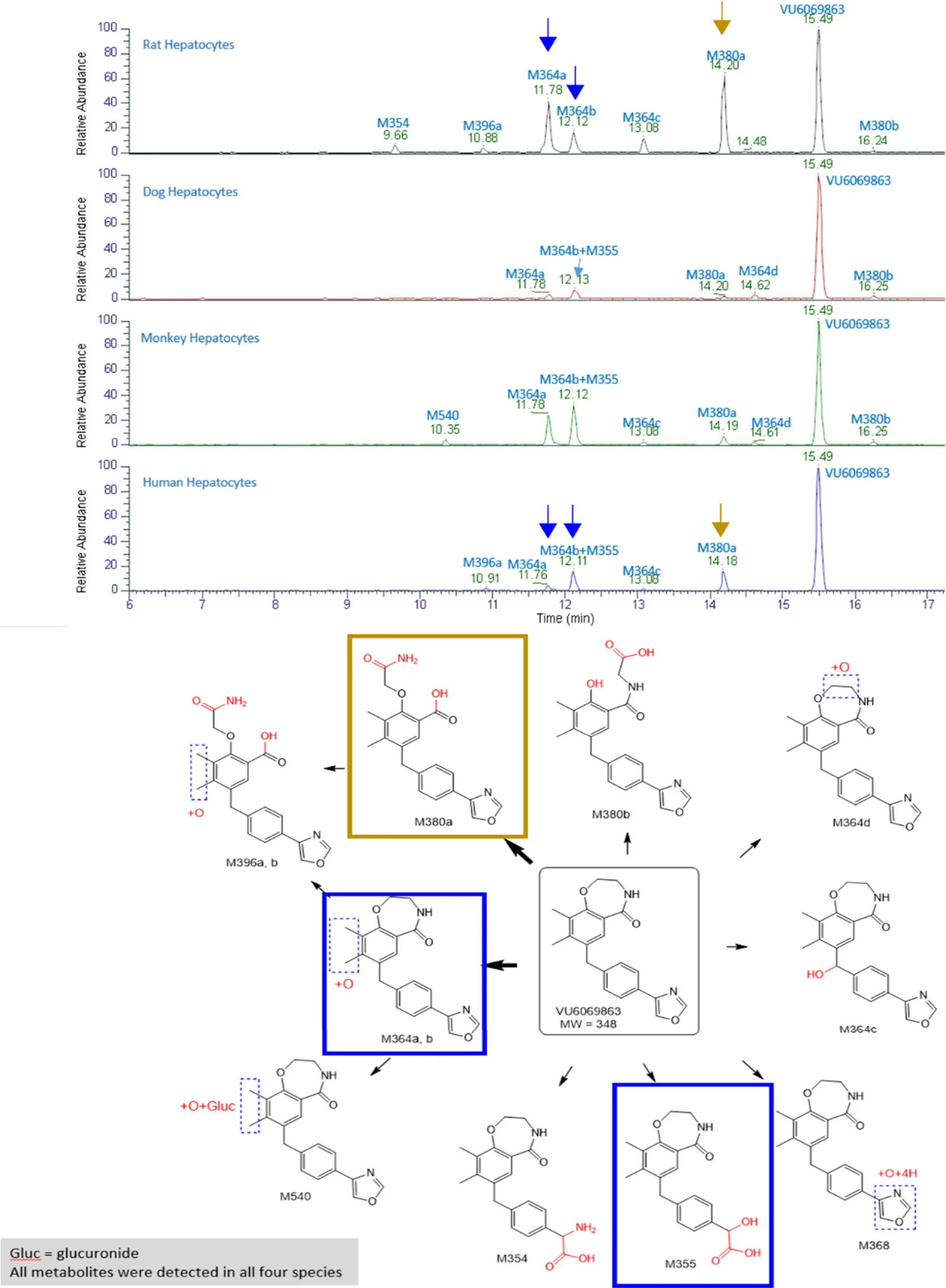
Metabolism of PAM **11** in rat, dog, monkey and human hepatocytes after a 4 h incubation period.

**Table 2:**
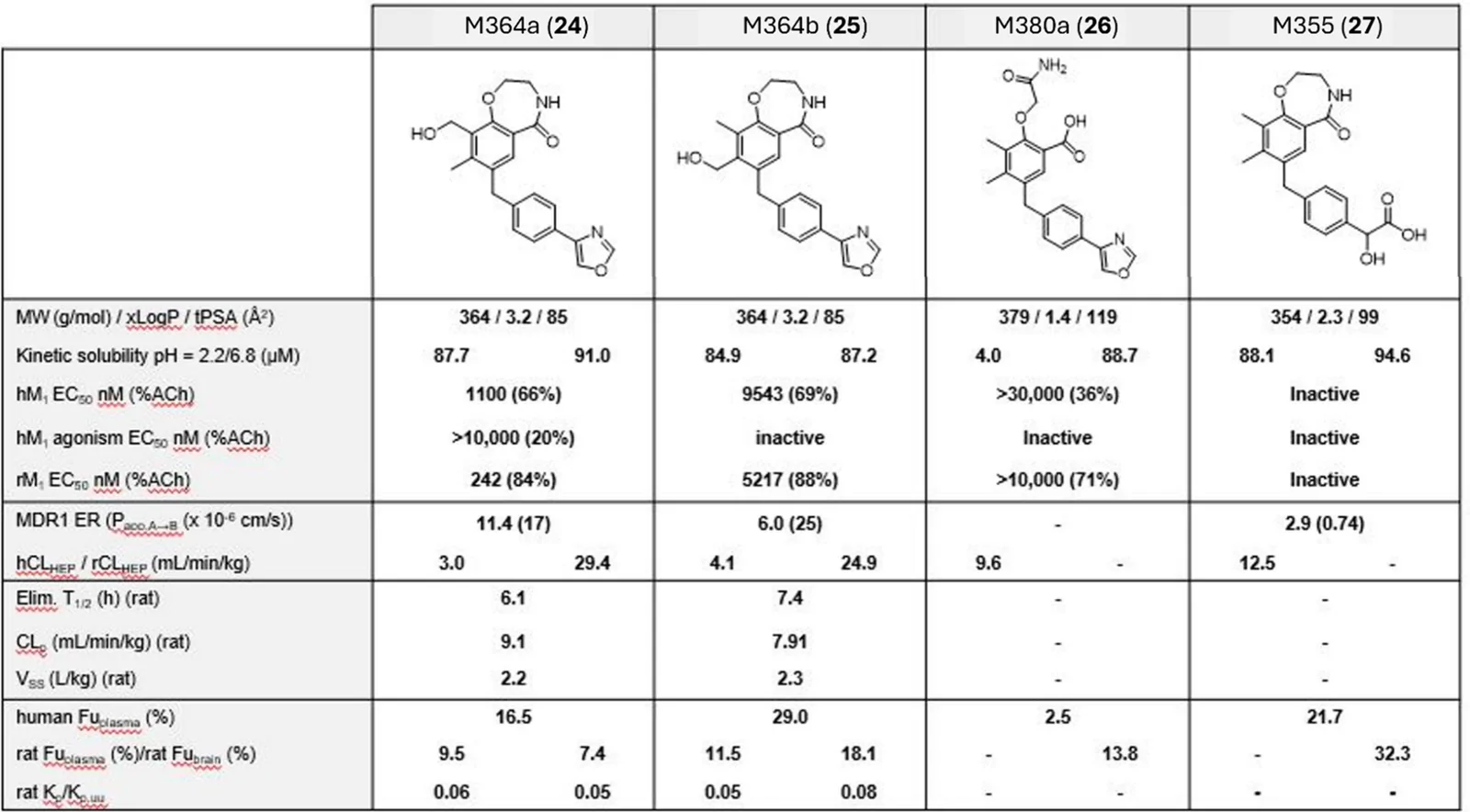
Pharmacology and DMPK Profile of Metabolites **24**–**27** of PAM **11**

With concerns of the potential accumulation of circulating peripheral metabolites notwithstanding, we proceeded with human PK and dose projections to decide whether PAM **11** could advance into IND-enabling studies. Human clearance was estimated using the 3 methods with the highest predictive accuracy as described in the Lombardo assessment (methods C7, C6a, and C3)^28^ as well as the rat single species scaling described by Hossea.^29^ Human V_ss_ was estimated using the eight methods with the highest predictive accuracy as described in the Lombardo assessment (methods V16, V16a, V7, V9, V10, V2, V3, and V6).^30^ For PAM **11**, our target *C*_min,tot,hum_ was 451 nM, the predicted human clearance was 3.12 mL/min/kg, V_ss_ was 0.71 L/kg, and human oral F was estimated at 72%. While this was reasonable, the human dose projection was high, with estimates between 169 mg BID based on rat clearance to >7,000 mg QD based on cyno and dog clearance. With the literature replete with idiosyncratic toxicology findings for drugs dosed at or above 100 mg QD in man^31^ coupled with the lengthy synthesis and projected cost of goods, PAM **11** could not advance further.

The necessity for a chemical optimization backup program for VU0467319 led our team to scaffold-hop known M_1_ ago-PAMs and *de novo* design a 3,4-dihydrobenzo­[*f*]­[1,4]­oxazepin-5-(*2H*)-one-derived M_1_ PAM (VU6069863, **11**) with minimal M_1_ agonism. PAM **11** exhibited an attractive pharmacological, physiochemical, and DMPK profile that resulted in robust efficacy in rat NOR (MED = 1 mg/kg) and CFC (MED = 0.3 mg/kg) without cholinergic AEs. The potential for accumulating metabolites and high human dose projections prevented PAM **11** from further development. However, PAM **11** represents another exceptional M_1_ PAM *in vivo* tool compound, devoid of cholinergic AEs and with a robust PK/PD relationship. Indeed, PAM **11** has become our standard positive control for the CFC assays. Further efforts to find a backup for VU0467319 are underway and will be reported in due course.

## Supplementary Material



## Notes

Additional experimental details, methods for the synthesis and characterization of all compounds, in vitro and in vivo DMPK protocols, and supplemental tables (PDF)

## Abbreviations

PAM: positive allosteric modulator
PBL: plasma/brain level
DMPK: drug metabolism and pharmacokinetics
AE: adverse event
M_1_: muscarinic acetylcholine receptor subtype 1
MED: minimum effective dose
NOR: novel object recognition
CFC: contextual fear conditioning
