# Supplementary material for: De Novo Design in an M1 Positive Allosteric Modulator Backup Program: The Discovery of VU6069863
Source: ACS Med Chem Lett. 2026 Aug 21;17(9):2085–94. doi: 10.1021/acsmedchemlett.6c00392 (PMC13573255; doi:10.1021/acsmedchemlett.6c00392)
Supplement: Supplementary file 1 [file ml6c00392_si_001.pdf]

# *De Novo* design in an $M_1$ Positive Allosteric Modulator back-up program: the discovery of VU6069863

Changho Han,[1,2]* Upendra Rathnayake,[1,2] Alison R. Gregro,[1,2] Julie L. Engers,[1,2] Christopher C. Presley,[1,2] Nathan D. Schley,[3] Analisa D. Thompson Gray,[1,2] Michael Bubser,[1,2] Hyekyung P. Cho,[1,2] Alice L. Rodriguez,[1,2] Valerie M. Kramlinger,[1,2] Olivier Boutaud,[1,2] Colleen M. Niswender,[1,2,4,5,6] Carrie K. Jones,[1,2,5] Darren W. Engers,[1,2] and Craig W. Lindsley*[1,2,3]

**Affiliation:**
[1]Warren Center for Neuroscience Drug Discovery, Vanderbilt University, Nashville, TN 37232, USA
[2]Department of Pharmacology, Vanderbilt University School of Medicine, Nashville, TN 37232, USA
[3]Department of Chemistry, Vanderbilt University, Nashville TN 37232, USA
[4]Vanderbilt Kennedy Center, Vanderbilt University Medical Center, Nashville, TN 37232, USA
[5]Vanderbilt Brain Institute, Vanderbilt University, Nashville, TN 37232, USA
[6]Vanderbilt Institute of Chemical Biology, Vanderbilt University, Nashville, TN 37232, USA

**To whom correspondence should be addressed at changho.han@vanderbilt.edu, craig.lindsley@vanderbilt.edu

## TABLE OF CONTENTS

Eurofins Lead Profiling Data....2
Effects on Ion Channels Expressed in Mammalian Cells....4
Procedures for Biological Experiments....8
General Methods....166
Synthetic Procedures and Spectra....18
Purity Analysis by LC-MS....56
X-Ray Structure....58
Supplemental Figures....59
References....62

## Eurofins Lead Profiling Data

**Table S1.** Eurofins Lead Profiling Screen Data - Study FR095-004497

This is a radioligand binding panel of 44 targets including GPCRs, ion channels, transporters and nuclear hormones. Biochemical assay results are presented as the percent inhibition of specific binding at a 10 μM concentration of VU6069863 (**11**).

| **Target/Protein** | **Species** | **% Inhibition at 10 μM** |
|---|---|---|
| Acetylcholinesterase | Human | 5 |
| Adenosine $A_{2A}$ | Human | 9 |
| Adrenergic $\alpha_{1A}$ | Human | 49 |
| Adrenergic $\alpha_{2A}$ | Human | 18 |
| Adrenergic $\beta_1$ | Human | 10 |
| Adrenergic $\beta_2$ | Human | 5 |
| Androgen (Testosterone) | Human | -10 |
| Calcium Channel L-Type, Nitrendipine | Rat | 42 |
| Cannabinoid $CB_1$ | Human | -27 |
| Cannabinoid $CB_2$ | Human | -9 |
| Cholecystokinin CCK1 | Human | -5 |
| Cyclooxygenase COX-1 | Human | 17 |
| Cyclooxygenase COX-2 | Human | -10 |
| Dopamine $D_1$ | Human | 2 |
| Dopamine $D_{2S}$ | Human | -22 |
| Endothelin $ET_A$ | Human | 19 |
| $GABA_A$, Flunitrazepam, Central | Rat | 21 |
| Glucocorticoid | Human | 5 |
| Glutamate, NMDA, Glycine | Rat | 8 |
| Histamine $H_1$ | Human | 1 |
| Histamine $H_2$ | Human | 8 |
| Lck TK Kinase | Human | 9 |
| Monoamine Oxidase A | Rat | 51 |
| Muscarinic $M_1$ | Human | 10 |
| Muscarinic $M_2$ | Human | 10 |
| Muscarinic $M_3$ | Human | -3 |
| Nicotinic Acetylcholine α4β2 | Human | -6 |
| Opiate $\delta_1$ (OP1, DOP) | Human | 15 |
| Opiate κ (OP2, KOP) | Human | 34 |
| Opiate μ (OP3, MOP) | Human | 4 |
| Phosphodiesterase (PDE3A) | Human | -4 |
| Phosphodiesterase (PDE4D2) | Human | 56 |
| Potassium Channel [$K_{ATP}$] | Rat | 3 |
| Potassium Channel hERG | Human | -2 |

| | | |
|---|---|---|
| Serotonin (5-$HT_{1A}$) | Human | 30 |
| Serotonin (5-$HT_{1B}$) | Human | 27 |
| Serotonin (5-$HT_{2A}$) | Human | 82 |
| Serotonin (5-$HT_{2B}$) | Human | 96 |
| Serotonin (5-$HT_3$) | Human | 8 |
| Sodium Channel, Site 2 | Rat | -7 |
| Transporter, Dopamine (DAT) | Human | 7 |
| Transporter, Norepinephrine (NET) | Human | 5 |
| Transporter, Serotonin (SERT) | Human | 14 |
| Vasopressin/Oxytocin ($V_{1a}$) | Human | 13 |

## Effects on Ion Channels Expressed in Mammalian Cells

**Table S2:** Effects of VU6069863 (**11**) on Ion Channels – Charles River Study 20496340

| Ion Channel | Test Article ID | Conc (μM) | Mean % Inhibition | Standard Deviation | Standard Error | n | Individual Data Points (% Inhibition) |
|---|---|---|---|---|---|---|---|
| hCav1.2 | VU6069863-06 | 10 | 50.9 | 13.0 | 6.5 | 4 | 35.2 |
| | | | | | | | 50.3 |
| | | | | | | | 51.3 |
| | | | | | | | 67.0 |
| hCav3.2 | VU6069863-06 | 10 | 66.3 | 7.2 | 3.6 | 4 | 76.8 |
| | | | | | | | 61.8 |
| | | | | | | | 61.4 |
| | | | | | | | 65.2 |
| hHCN2 | VU6069863-06 | 10 | 4.9 | 2.2 | 1.3 | 3 | 4.1 |
| | | | | | | | 7.4 |
| | | | | | | | 3.2 |
| hERG | VU6069863-06 | 10 | 18.9 | 6.5 | 3.2 | 4 | 13.6 |
| | | | | | | | 14.0 |
| | | | | | | | 20.7 |
| | | | | | | | 27.2 |
| hKv1.3 | VU6069863-06 | 10 | 54.6 | 5.3 | 2.2 | 6 | 48.6 |
| | | | | | | | 48.6 |
| | | | | | | | 60.5 |
| | | | | | | | 60.5 |
| | | | | | | | 54.2 |
| | | | | | | | 55.4 |
| hKv1.5 | VU6069863-06 | 10 | 34.6 | 6.3 | 2.8 | 5 | 31.6 |
| | | | | | | | 39.7 |
| | | | | | | | 28.5 |
| | | | | | | | 30.3 |
| | | | | | | | 42.8 |
| hKvLQT1/hminK | VU6069863-06 | 10 | 18.7 | 6.9 | 2.8 | 6 | 14.6 |
| | | | | | | | 25.7 |
| | | | | | | | 11.4 |
| | | | | | | | 14.4 |
| | | | | | | | 17.6 |
| | | | | | | | 28.5 |
| hNav1.5 (Tonic) | VU6069863-06 | 10 | 7.4 | 1.6 | 0.7 | 5 | 7.1 |
| | | | | | | | 5.6 |
| | | | | | | | 9.0 |
| | | | | | | | 6.3 |
| | | | | | | | 9.1 |
| hNav1.5 (Phasic) | VU6069863-06 | 10 | 16.0 | 3.4 | 1.5 | 5 | 18.1 |
| | | | | | | | 15.1 |
| | | | | | | | 16.5 |
| | | | | | | | 10.6 |
| | | | | | | | 19.5 |

**Table S3.** Effects of VU6069863 (**11**) on hCav1.2 Ion Channel Current – Study 20496340

| Test Article ID | $IC_{50}$ (μM) | Conc (μM) | Mean % hCav1.2 Inhibition | Standard Deviation | Standard Error | n | Individual Data Points (% Inhibition) |
|---|---|---|---|---|---|---|---|
| VU6069863-06 | 11.188 | 3 | 14.0 | 9.4 | 4.7 | 4 | 16.8 |
| | | | | | | | 2.8 |
| | | | | | | | 25.1 |
| | | | | | | | 11.4 |
| | | 10 | 48.3 | 12.4 | 6.2 | 4 | 32.5 |
| | | | | | | | 47.3 |
| | | | | | | | 51.0 |
| | | | | | | | 62.6 |
| | | 30 | 75.6 | 14.4 | 8.3 | 3 | 86.4 |
| | | | | | | | 81.1 |
| | | | | | | | 59.2 |
| | | 100 | 95.1 | 2.3 | 1.2 | 4 | 94.1 |
| | | | | | | | 94.4 |
| | | | | | | | 98.5 |
| | | | | | | | 93.3 |

**Table S4.** Effects of VU6069863 (**11**) on hCav3.2 Ion Channel Current – Study 20496340

| Test Article ID | $IC_{50}$ (μM) | Conc (μM) | Mean % hCav3.2 Inhibition | Standard Deviation | Standard Error | n | Individual Data Points (% Inhibition) |
|---|---|---|---|---|---|---|---|
| VU6069863-06 | 20.920 | 3 | 16.4 | 10.5 | 5.2 | 4 | 6.6 |
| | | | | | | | 9.9 |
| | | | | | | | 18.9 |
| | | | | | | | 30.0 |
| | | 10 | 32.1 | 5.4 | 2.4 | 5 | 26.1 |
| | | | | | | | 33.2 |
| | | | | | | | 40.2 |
| | | | | | | | 32.6 |
| | | | | | | | 28.4 |
| | | 30 | 55.7 | 10.6 | 4.0 | 7 | 53.0 |
| | | | | | | | 70.9 |
| | | | | | | | 52.3 |
| | | | | | | | 40.5 |
| | | | | | | | 51.5 |
| | | | | | | | 68.7 |

| | | | | | | | |
|---|---|---|---|---|---|---|---|
| | | | | | | | 52.8 |
| | | 100 | 92.5 | 0.8 | 0.5 | 3 | 91.6 |
| | | | | | | | 92.8 |
| | | | | | | | 93.0 |

**Table S5.** Effects of VU6069863 (**11**) on hERG Ion Channel Current – Study 20489053

| Test Article ID | $IC_{50}$ (µM) | Conc (µM) | Mean % hERG Inhibition | Standard Deviation | Standard Error | n | Individual Data Points (% Inhibition) |
|---|---|---|---|---|---|---|---|
| VU6069863-04 | 37.230 | 1 | 6.7 | 4.4 | 2.6 | 3 | 8.9 |
| | | | | | | | 1.6 |
| | | | | | | | 9.6 |
| | | 3 | 5.1 | 4.6 | 2.6 | 3 | 6.4 |
| | | | | | | | 0.0 |
| | | | | | | | 8.9 |
| | | 10 | 12.3 | 5.2 | 3.0 | 3 | 7.4 |
| | | | | | | | 11.9 |
| | | | | | | | 17.7 |
| | | 30 | 39.7 | 7.2 | 4.2 | 3 | 47.8 |
| | | | | | | | 34.1 |
| | | | | | | | 37.2 |
| | | 100* | 84.4 | 1.4 | 0.8 | 3 | 84.0 |
| | | | | | | | 86.0 |
| | | | | | | | 83.2 |

*There may be a solubility issue at this concentration as a visible precipitate was observed

**Table S6.** Effects of VU6069863 (**11**) on hKv1.3 Ion Channel Current – Study 20496340

| Test Article ID | $IC_{50}$ (µM) | Conc (µM) | Mean % hKv1.3 Inhibition | Standard Deviation | Standard Error | n | Individual Data Points (% Inhibition) |
|---|---|---|---|---|---|---|---|
| VU6069863-06 | 6.212 | 3 | 33.5 | 5.3 | 2.0 | 7 | 28.7 |
| | | | | | | | 39.6 |
| | | | | | | | 26.7 |
| | | | | | | | 36.4 |
| | | | | | | | 38.2 |
| | | | | | | | 36.6 |
| | | | | | | | 28.6 |
| | | 10 | 55.4 | 4.6 | 1.9 | 6 | 48.6 |
| | | | | | | | 53.3 |
| | | | | | | | 60.5 |

| | | | | | | | |
|---|---|---|---|---|---|---|---|
| | | | | | | | 60.5 |
| | | | | | | | 54.2 |
| | | | | | | | 55.4 |
| | | 30 | 93.5 | 3.2 | 1.4 | 5 | 97.5 |
| | | | | | | | 88.6 |
| | | | | | | | 93.1 |
| | | | | | | | 93.1 |
| | | | | | | | 95.0 |
| | | 100 | 93.7 | 6.3 | 2.8 | 5 | 85.3 |
| | | | | | | | 97.2 |
| | | | | | | | 100.2 |
| | | | | | | | 96.8 |
| | | | | | | | 88.8 |

## Procedures for Biological Experiments

**Calcium mobilization assays:**

All functional cell-based assays were performed in stable Chinese Hamster Ovary (CHO) cell lines constitutively expressing human $M_5$ or human $M_1$ receptors. For full muscarinic selectivity, CHO cells expressing human $M_3$, human $M_2$ plus $G_{qi5}$ or human $M_4$ plus $G_{qi5}$ were used. Cells were plated at 15,000 cells per 20 μL per well in black 384-well, TC-treated, clear-bottomed plates (Greiner) in Ham's F12 medium supplemented with 10% FBS and 20 mM HEPES. Cells were incubated overnight at 37 °C under 5% $CO_2$. The following day, the medium was removed and replaced with 1.2 μM Fluo-4 AM (Invitrogen) in assay buffer (Hank's Balanced Salt Solution supplemented with 20 mM HEPES and 2.5 mM Probenecid, pH 7.4) and the cells were incubated for 50 minutes at 37 °C under 5% $CO_2$. Dye was then removed and replaced with 20 μL of fresh assay buffer. Test compounds at a 10 mM concentration in DMSO were serially diluted in DMSO (either 1:3 or 1:5 dilution) to create a 10-point concentration series. The DMSO solutions were then diluted in assay buffer resulting in compound solutions at 2-times the final assay concentration with the highest assay concentration of 30 μM. The compound plate, cell plate, and plates containing $EC_{20}$ and $EC_{80}$ acetylcholine concentrations were placed in a Hamamatsu FDSS 6000 or 7000 kinetic imaging plate reader equipped to measure $Ex_{480}/Em_{540}$ fluorescence. Data were collected at 1 frame per second. After 2 seconds of collecting baseline fluorescence, 20 μL of the compound solutions were added to the cell plate. This was followed by the addition of an $EC_{20}$ concentration of acetylcholine at 142 seconds. At 267 seconds, an $EC_{80}$ concentration of acetylcholine was added along with a maximally effective acetylcholine concentration in wells not containing a compound to allow data normalization. The fluorescence signal was collected for a total of 300 seconds. Compound concentration response curves (CRCs) were collected in triplicate across three separate plates. Data were imported and analyzed in Dotmatics Informatics software. by normalizing all data in the individual kinetic traces to the initial fluorescence read. The magnitude of each agonist addition was then determined and normalized to the average maximum response. This percent maximum response was plotted against log[compound] and fit to a four parameter logistical equation to determine $\log(IC_{50})$. The $IC_{50}$ determined using the $EC_{80}$ of acetylcholine is the value reported. Compound CRC curves that did not plateau below 10% $ACh_{max}$ were assigned a low potency value of >10 μM.

**Drug Metabolism Methods:**

***In vitro***

**Plasma protein binding and Brain homogenate binding:** Determination of fraction unbound ($f_u$) in plasma was conducted in vitro via equilibrium dialysis using HTDialysis (HTD) membrane plates. The top half of the plate was filled with 100 uL of Dubelco's Phosphate Buffered Saline, pH 7.4 (DPBS). Compounds were diluted into plasma from each species (5 µM final concentration), which was aliquoted in triplicate to the 'bottom half' of the prepared HTD plate wells. The HTD plate was sealed and incubated for 6 hours at 37 ˚C. Following incubation, each well (both top and bottom halves) was transferred (20 µL) to the corresponding wells of a 96-shallow-well (V-bottom) plate. The daughter plates were then matrix-matched (DPBS side wells received equal volume of plasma, and plasma side wells received equal volume of DPBS), and extraction solution (120 µL; acetonitrile containing 50 nM carbamazepine as IS) was added to all wells of both daughter plates to precipitate protein and extract the test article. The plates were then sealed and centrifuged (3500 rcf) for 10 minutes at ambient temperature. Supernatant (60 µL) from each well of the daughter plates was then transferred to the corresponding wells of new daughter plates (96-shallow-well, V bottom) containing water (Milli-Q, 60 µL/well), and the plates were sealed in preparation for LC-MS/MS analysis (see LC-MS/MS analysis method below).

The unbound fraction ($f_u$) was calculated following the equation below, and mean values for each species were calculated from 3 replicates.

A similar approach was used to determine the degree of brain homogenate binding, which employed the same methodology and procedure with the following modifications: 1) a final compound concentration of 1 µM was used, 2) naïve rat brains were homogenized in DPBS (1:3 composition of brain: DPBS, w/w) using a Mini-Bead Beater™ machine in order to obtain brain homogenate, which was then treated in the same manner as the plasma samples in the previously described plasma protein binding assay. Fraction unbound for both plasma and brain samples was determined using Equation 4.

$$f_u = \frac{Conc_{buffer}}{Conc_{plasma}}$$

Equation 4 Determination of fraction unbound in plasma.

The diluted fraction unbound ($f_u2$) in brain was calculated in the same manner by using brain homogenate rather than plasma. Undiluted fraction unbound for the brain was calculated using Equation 5

$$f_u = \frac{1/4}{\left\{\left(\frac{1}{fu2}\right) - 1\right\} + 1/4}$$

Equation 5 Determination of fraction unbound in brain. $F_u2$ represents the diluted fraction unbound.

**Intrinsic clearance:** Human or rat hepatic microsomes (0.5 mg/mL) and 1 μM test compound were incubated in 100 mM potassium phosphate pH 7.4 buffer with 3 mM $MgCl_2$ at 37 °C with constant shaking. After a 5 min preincubation, the reaction was initiated by the addition of NADPH (1 mM). At selected time intervals (0, 3, 7, 15, 25, and 45 min), aliquots were taken and subsequently placed into a 96-well plate containing cold acetonitrile with internal standard (50 ng/mL carbamazepine). Plates were then centrifuged at 3000 rcf (4 °C) for 10 min, and the supernatant was transferred to a separate 96-well plate and diluted 1:1 with water for LC/MS/MS analysis. The *in vitro* half-life ($_{1/2}$, min, Eq. 1), intrinsic clearance ($CL_{int}$, mL/min/kg, Eq. 2), and subsequent predicted hepatic clearance ($CL_{hep}$, mL/min/kg, Eq. 3) was determined employing the following equations:

(1) $T_{1/2} = \frac{Ln(2)}{K}$

where k represents the slope from linear regression analysis of the natural log percent remaining of a test compound as a function of incubation time

(2) $CL_{int} = \frac{0.693}{in\ vitro T_{1/2}}\ x\ \frac{mL\ incubation}{mg\ microsomes}\ x\ \frac{45\ mg\ microsomes}{gram\ liver}\ x\ \frac{20^{a}\ gram\ liver}{kg\ body\ wt}$

[a]scale-up factors: of 20 (human) or 45 (rat)

(3) $CL_{hep} = \frac{Q_h \cdot CL_{int}}{Q_h + CL_{int}}$

where $Q_h$ (hepatic blood flow, mL/min/kg) is 21 (human) or 70 (rat).

**LC/MS/MS Bioanalysis of Samples from Plasma Protein Binding and Intrinsic Clearance Assays:**

Samples were analyzed on a Thermo Electron TSQ Quantum Ultra triple quad mass spectrometer (San Jose, CA) via electrospray ionization (ESI) with two Themo Electron Accella pumps (San Jose, CA), and a Leap Technologies CTC PAL autosampler (Carrboro, NC). Analytes were separated by gradient elution on a dual column system with two Thermo Hypersil Gold (2.1 x 30 mm, 1.9 μm) columns (San Jose, CA) thermostated at 40 °C. HPLC mobile phase A was 0.1% formic acid in water and mobile phase B was 0.1% formic acid in acetonitrile. The gradient started at 10% B after a 0.2 min hold and was linearly increased to 95% B over 0.8 min; hold at 95% B for 0.2 min; returned to 10% B in 0.1 min. The total run time was 1.3 min and the HPLC flow rate was 0.8 mL/min. While pump 1 ran the gradient method, pump 2 equilibrated the alternate column isocratically at 10% B. Compound optimization, data collection, and processing were performed using Thermo Electron's QuickQuan software (v2.3) and Xcalibur (v2.0.7 SP1).

***In vivo* DMPK experimental:**

Determination of brain to plasma ratio:

*Animal care and use*

All animal study procedures were approved by the Institutional Animal Care and Use Committee and were conducted in accordance with the National Institutes of Health regulations of animal care covered in Principles of Laboratory Animal Care (National Institutes of Health). All rats were fasted overnight prior to testing.

*In-life phase*

For determination of the brain over plasma ratio ($K_p$), compounds were formulated in 8% ethanol, 32% PEG400 and 60% DMSO (*v/v/v*) and administered as a single 0.2 mg/kg IV dose (1 mL/kg) to male, Sprague Dawley rats ($n$ = 1) via injection into a surgically-implanted jugular vein catheter. At 15 min post dosing, blood sample was collected into chilled, $K_2$EDTA anticoagulant-fortified tube and immediately placed on wet ice. The blood sample was then centrifuged (1700 rcf, 5 minutes, 4 ˚C) to obtain plasma sample. At the same post-administration time point, whole

brain sample was obtained by rapid dissection, rinsed with PBS, and immediately frozen in individual tissue collection box (dry ice). All brain and plasma samples were stored at -80 ˚C until analysis by LC-MS/MS.

*Sample Analysis:* Concentrations in plasma and brain homogenates were quantified by liquid chromatography tandem mass spectrometry (LC-MS/MS). Whole brains were homogenized in 3 mL of 70:30 IPA:water in a mini bead beater for 3 min, and centrifuged at 3,500 g for 5 min. 5 uL of the supernatant was diluted in 15 uL of blank plasma for quantification of the analytes. Plasma samples were centrifuged at 3,500 g for 5 min. A standard curve was generated by diluting the analytes DMSO stocks with blank plasma to obtain a final concentration of 10,000 ng/ml followed by a serial dilution down to 0.5 ng/ml. Quality controls were generated by a serial dilution of the 5,000 ng/ml standard curve solution in blank plasma to obtain 3 concentrations of 500, 50, and 5 ng/ml. 20 uL of brain diluted in plasma, plasma, blank plasma, standard curve and QC samples were loaded in a V-bottom 96-well plate. 120 uL of acetonitrile containing 0.05 uM carbamazepine (internal standard) was added to each well and the plate was centrifuged at 3,500 g for 5 min. 60 uL of the supernatant of each well (protein free) was transferred to a new 96-well plate containing 60 uL of water. The plates were sealed for analysis by LC-MS/MS.

Plasma and brain tissue samples originating from *in vivo* studies were analyzed by electrospray ionization using an AB Sciex Q-TRAP 5500 (Foster City, CA) that was coupled to a Shimadzu LC-20AD pump (Columbia, MD) and a Leap Technologies CTC PAL auto-sampler (Carrboro, NC). Analytes were separated by gradient elution using a C18 column (3 x 50 mm, 3 mm; Fortis Technologies Ltd, Cheshire, UK) that was thermostated at 40 °C. HPLC mobile phase A was 0.1% formic acid in water (pH unadjusted); mobile phase B was 0.1% formic acid in acetonitrile (pH unadjusted). A 10% B gradient was held for 0.2 min and was linearly increased to 90% B over 0.8 min, with an isocratic hold for 0.5 min, before transitioning to 10% B over 0.05 min. The column was re-equilibrated (1 min) before the next sample injection. The total run time was 2.55 min, and the HPLC flow rate was 0.5 ml/min. The source temperature was set at 500 °C, and mass spectral analyses were performed using a Turbo-Ion spray source in positive ionization mode (5.0-kV spray voltage) and using multiple-reaction monitoring of transitions specific for the analytes. All data were analyzed using AB Sciex Analyst 1.5.1 software.

Brain plasma concentration ratio ($K_p$) was calculated by dividing brain concentration by plasma concentration for each animal. Unbound brain to unbound plasma concentration ratio ($K_{p,uu}$) is calculated using the following formula: $K_{p,uu}$ = (Brain ng/g x brain fu)/(plasma ng/ml x plasma fu).

**Pharmacokinetic profiles in rats following oral single escalating doses**

Single escalating oral dosing in Sprague-Dawley rats was performed at Frontage Laboratories according to their non-GLP Standard Operating Procedure and IACUC protocols. In short, compounds were formulated in 10% Tween 80 in water and dosed at 10 mg/kg. At different times, arterial blood was collected from a femoral artery catheter, and compound concentration was determined in plasma by LC-MS/MS following their non-GLP protocol. PK parameters were determined using Phoenix WinNonlin software (version 6.3).

***In vitro* determination of blood-brain barrier penetration potential**

Blood-brain barrier penetration was determined using MDR1-MDCK cell monolayers by Absorption Systems, following their protocol. In short, compounds were incubated at 5 mM final concentration on one side of the cell monolayer for 2 hours. Compounds concentration on either side of the monolayer was determined by LC-MS/MS and apparent permeability and efflux ratio were determined as described in Wang, Q. et al.[1]

**Behavioral Studies Methods**

*Animals.* Adult male Sprague-Dawley rats weighing 275 - 299 g were obtained from Envigo (Indianapolis, Indiana) for the behavioral and pharmacokinetic studies. Rats were group-housed (2 - 3 animals per cage) under a 12 h light/12-h dark cycle with food and water ad libitum.

Adult male C57BL/6J mice aged 8 – 10 weeks were obtained from Jackson Laboratories (Bar Harbor, Maine) and were group-housed (5 animals per cage) under a 12 h light/12-h dark cycle with food and water ad libitum.

All animal studies were approved by the Vanderbilt University Institutional Animal Care and Use Committee and followed the guidelines set forth by the *Guide for the Care and Use of Laboratory Animals*.

***Novel Object Recognition Test.*** This study was carried out during the inactive phase of the rats (light phase) and all training and testing procedures were performed under red light conditions. The Novel Object Recognition task was performed as described previously.[1] For 2 consecutive days, rats were individually habituated for 10 min in an empty black composite chamber (40 cm × 64 cm × 33 cm). On the following day, vehicle (10% Tween 80 in milliQ water) or VU6069863 (0.3, 0.56, 1, or 1.8 mg/kg) was administered to the animals by oral gavage (p.o.), then rats were returned to their home cage. 60 min later, rats were placed for 10 min in the novel object recognition chamber containing two identical objects (either two 590-mL Gatorade® bottles filled with water or two weighted stainless steel pipette sterilization boxes [5.1 cm × 5.1 cm × 23.5 cm] and subsequently returned to their home cage. 24 h later, rats were returned to the chamber in a configuration where one of the familiar (previously exposed) objects remained while the other familiar object was replaced by a novel object. For each treatment group, the object type serving as familiar and novel was counterbalanced in a pseudo-randomized fashion. The behavior of each rat exploring the familiar and novel objects was video recorded for 3 min for subsequent analysis. Object exploration was defined as sniffing of the objects that was accompanied by active vibrissae movements. The time spent exploring each object was scored by an observer blinded to both the experimental treatment condition and the object types representing novel and familiar objects.

*Data Analysis.* For each rat the recognition index was calculated as follows:

$$Recognition\ Index\ = \frac{(\text{time exploring Novel Object} - \text{time exploring Familiar Object})}{(\text{time exploring Novel Object} + \text{time exploring Familiar Object})}$$

Thus, recognition indices of 1.0, 0, and -1.0 indicate exclusive exploration of Novel Object, equal exploration of Novel and Familiar Object, and exclusive exploration of Familiar Object, respectively.

***Contextual Fear Conditioning.*** Studies were conducted using conditioning chambers in sound attenuating cubicles equipped with a stainless steel grid floor for shock delivery and a video camera for recording freezing behavior (Med Associates, Fairfax, Vermont). Rats were acclimated to the apparatus for 3 days (10 minutes each day) prior to conditioning. On the training (conditioning) day, rats were pretreated by oral gavage with Vehicle (10% Tween 80) or VU6069863 (0.03, 0.1, 0.3, 1, or 3 mg/kg) 60 minutes before being placed in the conditioning chamber that contained 1 mL 10% vanilla extract as odor cue.[2] After a 180-sec habituation period in the chamber, a tone

was presented (30s tone,3kHz, 80db) that co-terminated with a single foot shock (1 second, 0.5 mA). Rats were left in the chamber for an additional 129 before being returned to their home cages. Approximately 24 h after conditioning, rats were returned to the identical conditioning chamber and odor cue for a 4-min session to assess freezing behavior, i.e., motionless posture, excluding respiratory movements, in the absence of foot shock using the automated conditioned freezing analysis software (Med Associates).

***Seizure liability studies.*** Mice were intraperitoneally injected with Vehicle (10% Tween 80), PAM **11** (100 mg/kg), or BQCA (100 mg/kg) and their behavior was scored 15, 30, 60, 180, and 360 min afterwards using a Racine scale.[3] Animals were observed for seizure activity using the following parameters:  0 = no behavioral alteration; 1 = immobility, mouth and facial movements, facial clonus; 2 = head nodding, forelimb and/or tail extension, rigid posture; 3 = forelimb clonus, repetitive movements; 4 = rearing, forelimb clonus with rearing, rearing and falling; 5 = continuous rearing and falling, jumping; 6 = severe tonic-clonic seizures.

*Statistics.* Data from the NOR and CFC studies are presented as means ± S.E.M. and were analyzed by one-way ANOVA followed by Dunnett’s test using GraphPad Prism 10 (GraphPad Software, San Diego, CA). Data from the seizure liability studies are presented as means ± S.D. and were analyzed by two-way ANOVA followed by Šídák's multiple comparisons tests using GraphPad Prism 10.

## General Methods

All NMR spectra were recorded on a 400 MHz AMX Bruker NMR spectrometer. $^1H$ and $^{13}C$ chemical shifts are reported in δ values in ppm downfield with the deuterated solvent as the internal standard. Data are reported as follows: chemical shift, multiplicity (s = singlet, d = doublet, t = triplet, q = quartet, b = broad, m = multiplet), integration, coupling constant (Hz). Low resolution mass spectra were obtained on an Agilent 6120/6150 or Waters QDa (Performance) SQ MS with ESI source. *Method A (Agilent 6120/6150):* MS parameters were as follows: fragmentor: 70, capillary voltage: 3000 V, nebulizer pressure: 30 psig, drying gas flow: 13 L/min, drying gas temperature: 350 ºC. Samples were introduced via an Agilent 1290 UHPLC comprised of a G4220A binary pump, G4226A ALS, G1316C TCC, and G4212A DAD with ULD flow cell. UV absorption was generally observed at 215 nm and 254 nm with a 4 nm bandwidth. Column: Waters Acquity BEH C18, 1.0 x 50 mm, 1.7 um. Gradient conditions: 5% to 95% $CH_3CN$ in $H_2O$ (0.1% TFA) over 1.4 min, hold at 95% $CH_3CN$ for 0.1 min, 0.5 mL/min, 55 ºC. *Method B (Agilent 6120/6150):* MS parameters were as follows: fragmentor: 100, capillary voltage: 3000 V, nebulizer pressure: 40 psig, drying gas flow: 11 L/min, drying gas temperature: 350 ºC. Samples were introduced via an Agilent 1200 HPLC comprised of a degasser, G1312A binary pump, G1367B HP-ALS, G1316A TCC, G1315D DAD, and a Varian 380 ELSD (if applicable). UV absorption was generally observed at 215 nm and 254 nm with a 4 nm bandwidth. Column: Thermo Accucore C18, 2.1 x 30 mm, 2.6 um. Gradient conditions: 7% to 95% $CH_3CN$ in $H_2O$ (0.1% TFA) over 1.6 min, hold at 95% $CH_3CN$ for 0.35 min, 1.5 mL/min, 45 ºC. *Method C (Waters QDa (Performance) SQ MS)*: MS parameters were as follows: cone voltage: 15 V, capillary voltage: 0.8 kV, probe temperature: 600° C. Samples were introduced via an Acquity I-Class PLUS UPLC comprised of a BSM, FL-SM, CH-A, and PDA. UV absorption was generally observed at 215 nm and 254 nm; 4 nm bandwidth. Column: Phenomenex EVO C18, 1.0 x 50 mm, 1.7 um. Column temperature: 55° C. Flow rate: 0.4 mL/min. Default gradient: 5% to 95% $CH_3CN$ (0.05% TFA) in $H_2O$ (0.05% TFA) over 1.4 min (curve 6), hold at 95% $CH_3CN$ for 0.1 min. "Polar" (2% to 70% $CH_3CN$ (0.05% TFA) in $H_2O$ (0.05% TFA) over 0.8 min (curve 6), transition to 95% $CH_3CN$ over 0.1 min (curve 6), hold at 95% $CH_3CN$ for 0.6 min.) and "Non-Polar" (40% to 95% $CH_3CN$ (0.05% TFA) in $H_2O$ (0.05% TFA) over 1.4 min (curve 6), hold at 95% $CH_3CN$ for 0.1 min.) gradients were also available. *Method D (Waters QDa (Performance) SQ MS):* MS parameters were as follows: cone voltage: 15 V, capillary voltage: 0.8 kV, probe temperature: 600° C.

Samples were introduced via an Acquity I-Class PLUS UPLC comprised of a BSM, FL-SM, CH-A, and PDA. UV absorption was generally observed at 215 nm and 254 nm with a 4 nm bandwidth. Column: Phenomenex EVO C18, 1.0 x 50 mm, 1.7 um. Column temperature: 55° C. Flow rate: 0.4 mL/min. Default gradient: 5% to 95% $CH_3CN$ in $H_2O$ (5 mM $NH_4HCO_3$) over 1.4 min (curve 6), hold at 95% $CH_3CN$ for 0.1 min. "Polar" (2% to 70% $CH_3CN$ in $H_2O$ (5 mM $NH_4HCO_3$) over 0.8 min (curve 6), transition to 95% $CH_3CN$ over 0.1 min (curve 6), hold at 95% $CH_3CN$ for 0.6 min.) and "Non-Polar" (40% to 95% $CH_3CN$ in $H_2O$ (5 mM $NH_4HCO_3$) over 1.4 min (curve 6), hold at 95% $CH_3CN$ for 0.1 min.) gradients were also available. High resolution mass spectra were obtained on an Agilent 6540 UHD Q-TOF with ESI source. MS parameters were as follows: fragmentor: 150, capillary voltage: 3500 V, nebulizer pressure: 60 psig, drying gas flow: 13 L/min, drying gas temperature: 275 ºC. Samples were introduced via an Agilent 1200 UHPLC comprised of a G4220A binary pump, G4226A 3 ALS, G1316C TCC, and G4212A DAD with ULD flow cell. UV absorption was observed at 215 nm and 254 nm with a 4 nm bandwidth. Column: Agilent Zorbax Extend C18, 1.8 µm, 2.1 x 50 mm. Gradient conditions: 5% to 95% $CH_3CN$ in $H_2O$ (0.1% formic acid) over 1 min, hold at 95% $CH_3CN$ for 0.1 min, 0.5 mL/min, 40 ºC. For compounds that were purified on a Gilson preparative reversed-phase HPLC, the system comprised of a 333 aqueous pump with solvent selection valve, 334 organic pump, GX 271 or GX-281 liquid hander, two column switching valves, and a 155 UV detector. UV wavelength for fraction collection was user-defined, with absorbance at 254 nm always monitored. Method 1: Phenomenex Axia-packed Luna C18, 30 x 50 mm, 5 µm column. Mobile phase: $CH_3CN$ in $H_2O$ (0.1% TFA). Gradient conditions: 0.75 min equilibration, followed by user defined gradient (starting organic percentage, ending organic percentage, duration), hold at 95% $CH_3CN$ in $H_2O$ (0.1% TFA) for 1 min, 50 mL/min, 23 ºC. Method 2: Phenomenex Axia packed Gemini C18, 50 x 250 mm, 10 um column. Mobile phase: $CH_3CN$ in $H_2O$ (0.1% TFA). Gradient conditions: 7 min equilibration, followed by user defined gradient (starting organic percentage, ending organic percentage, duration), hold at 95% $CH_3CN$ in $H_2O$ (0.1% TFA) for 7 min, 120 mL/min, 23 ºC. Solvents for extraction, washing and chromatography were HPLC grade. All compounds are >95% purity by HPLC.

**Safety statement:** No unexpected or unusually high safety hazards were encountered.

# Synthetic Procedures and Spectra

**Scheme 1.** Synthesis of VU6069863 (**11**).[a]

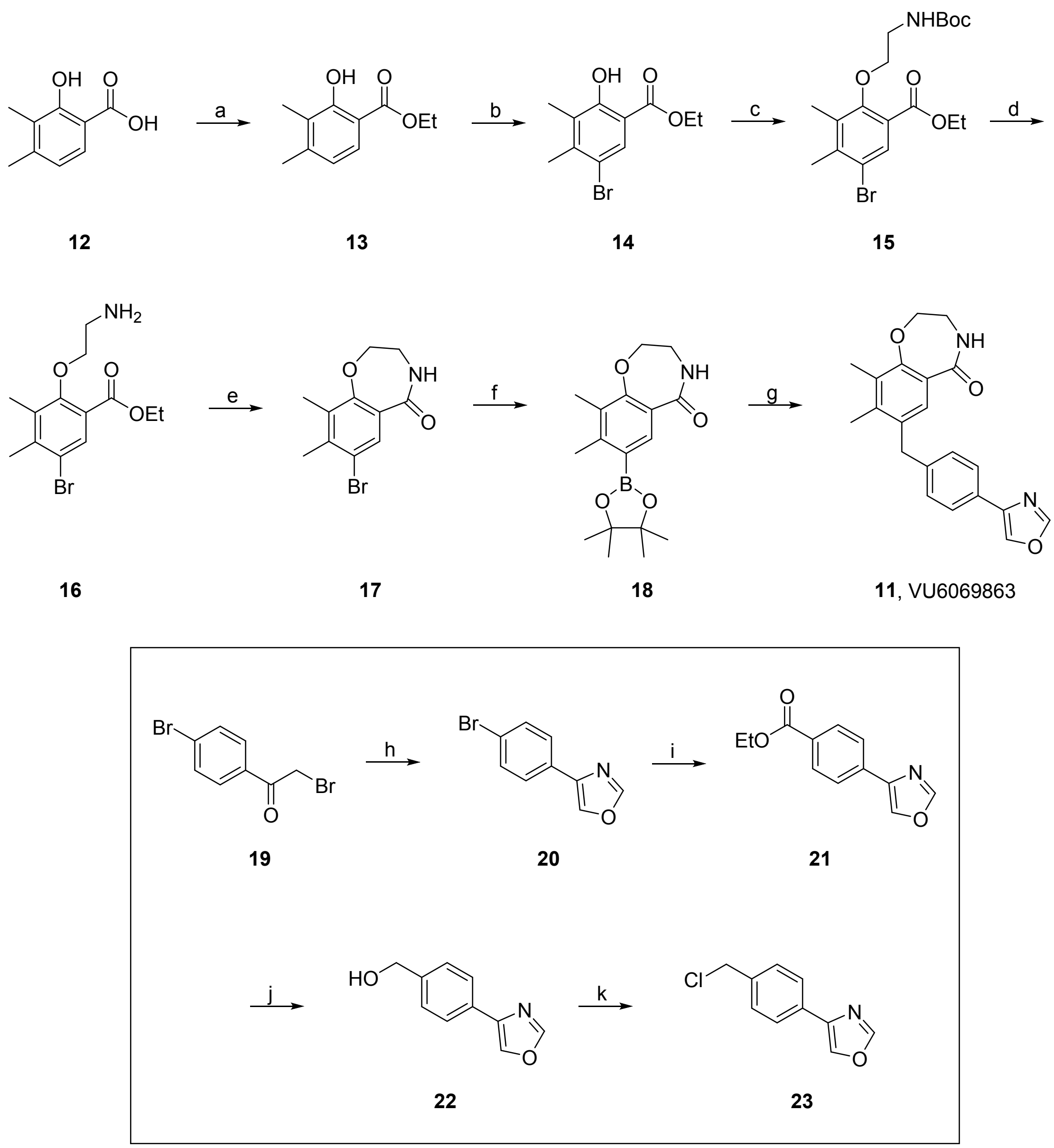


[a]Reagents and conditions: (a) EtOH, $H_2SO_4$, 100 °C, 16 h, 82%; (b) $Br_2$, $CHCl_3$, 0 °C, 82%; (c) $HOCH_2CH_2NHBoc$, $PPh_3$, DIAD, THF, rt; (d) 4 N HCl in dioxane, rt; (e) $K_2CO_3$, EtOH, 80 °C, 18 h, 76% over three steps; (f) Bis(pinacolato)diboron, KOAc, Pd(dppf)$Cl_2$, 1,4-dioxane, 90 °C, 88%; (g) **23**, $Cs_2CO_3$, Pd(dppf)$Cl_2$, 1,4-dioxane/$H_2O$, 100 °C, 80%; (h) $HCO_2NH_2$, HCOOH, 115 °C, 2.5 h, 32%; (i) CO (g), $Et_3N$, $Pd(OAc)_2$, dppp, EtOH/DMSO, 75 °C, 88%; (j) 2M $LiAlH_4$, THF, 0 °C, 83%; (k) $SOCl_2$, DCM, rt, 97%.

## Step a: Preparation of ethyl 2-hydroxy-3,4-dimethylbenzoate (13)

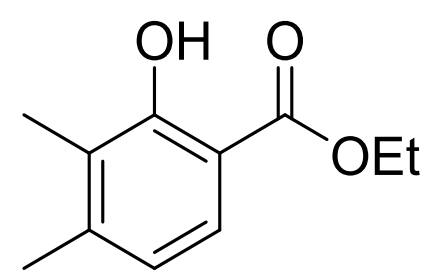

**13**

To a solution of 2-hydroxy-3,4-dimethylbenzoic acid (51 g, 307 mmol) in ethanol (614 mL) was added sulfuric acid (163.6 mL, 3069 mmol) dropwise at 0 °C. The reaction mixture was stirred at 100 °C under refluxing condition for 16 h. The reaction mixture was poured into ice-water and the resulting solid was collected by vacuum filtration. The crude product was dissolved in DCM and washed with sat $NaHCO_3$ to remove any unreacted carboxylic acid. The organic layer was dried over $Na_2SO_4$ and concentrated under vacuum to afford the title compound (55.4 g, 93% yield) as a brown oil which was taken to the next step without purification. $^1$H NMR (400 MHz, DMSO-$d_6$) δ 11.04 (s, 1H), 7.55 (d, $J$ = 8.1 Hz, 1H), 6.77 (d, $J$ = 8.1 Hz, 1H), 4.35 (q, $J$ = 7.1 Hz, 2H), 2.26 (s, 3H), 2.10 (s, 3H), 1.33 (t, $J$ = 7.1 Hz, 3H); $^{13}$C NMR (101 MHz, $CDCl_3$) δ 170.9, 159.9, 145.0, 126.6, 124.7, 120.7, 109.9, 61.3, 20.8, 14.4, 11.3; HRMS (ESI/Q-TOF) m/z: $[M+H]^+$ Calcd for $C_{11}H_{14}O_3$ 195.1016, Found 195.1017.

**Step b: Preparation of ethyl 5-bromo-2-hydroxy-3,4-dimethylbenzoate (14)**

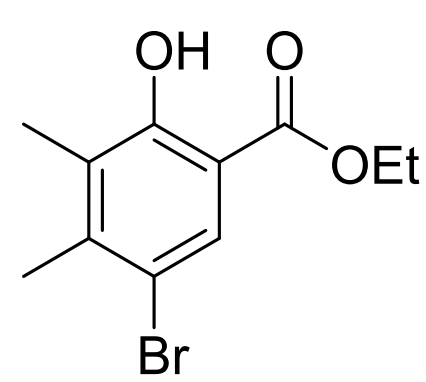

**14**

To a solution of ethyl 2-hydroxy-3,4-dimethylbenzoate (55.4 g, 285.4 mmol) in chloroform (1.3 L) was added bromine (20.5 mL, 399.6 mmol) dropwise at 0 °C. The resulting solution was stirred at 0 °C for 15 min. The reaction mixture was quenched by adding sat. soln. $Na_2S_2O_3$ and stirred for another 15 min. Layers were separated. The aqueous layer was extracted with DCM. The combined organic layers were washed with brine, dried over $Na_2SO_4$ and concentrated under reduced pressure to afford the title compound (76.5 g, 98% yield) as a brown solid which was taken to the next step without further purification. $^1$H NMR (400 MHz, DMSO-$d_6$) 10.95 (s, 1H), 7.79 (s, 1H), 4.37 (q, $J$ = 7.1 Hz, 2H), 2.35 (s, 3H), 2.19 (s, 3H), 1.34 (t, $J$ = 7.1 Hz, 3H); $^{13}$C NMR

(101 MHz, DMSO-$d_6$) δ 168.7, 157.7, 143.5, 129.2, 126.7, 113.6, 110.9, 61.8, 20.2, 13.9, 12.3; ES-MS $[M+H]^+$ = 273.0 and 275.0; HRMS (ESI/Q-TOF) m/z: $[M+H]^+$ Calcd for $C_{11}H_{13}BrO_3$ 275.0101, Found 275.0099.

**Step c: Preparation of ethyl 5-bromo-2-(2-((*tert*-butoxycarbonyl)amino)ethoxy)-3,4-dimethylbenzoate (15)**

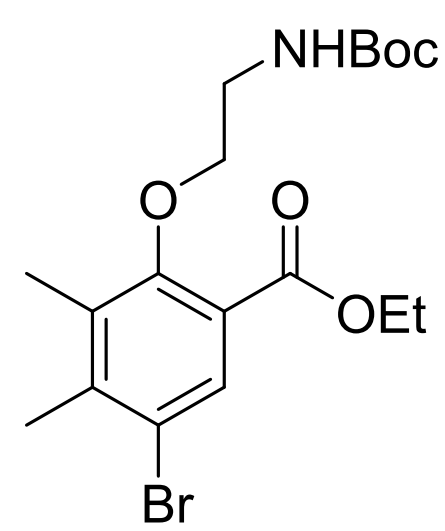


**15**

Under nitrogen atmosphere, to a solution of ethyl 5-bromo-2-hydroxy-3,4-dimethylbenzoate 40.0 g, 146.5 mmol) in THF (666 mL) was added *N*-Boc-ethanolamine (47.2 g, 293 mmol) and triphenylphosphine (64.5 g, 234.3 mmol). The resulting solution was cooled to 0 °C and di-*tert*-butyl azodicarboxylate (54.0 g, 234.3 mmol) was added portion-wise. After 12 h at rt, the mixture was cooled to 0 °C and recharged with *N*-Boc-ethanolamine (47.2 g, 293 mmol), triphenylphosphine (64.5 g, 234.3 mmol) and di-*tert*-butyl azodicarboxylate (54.0 g, 234.3 mmol). The step above was repeated after 24 h until the starting material was consumed. The reaction mixture was diluted with water and DCM. Layers were separated. Aqueous layer was extracted with DCM (3x). The combined extracts were dried over $Na_2SO_4$, filtered and concentrated to afford the title compound (411.5 g) as a brown viscous oil which was carried forward without further purification. ES-MS $[M+H-Boc]^+$ = 316.1 and 318.1.

**Step d: Preparation of ethyl 2-(2-aminoethoxy)-5-bromo-3,4-dimethylbenzoate (16)**

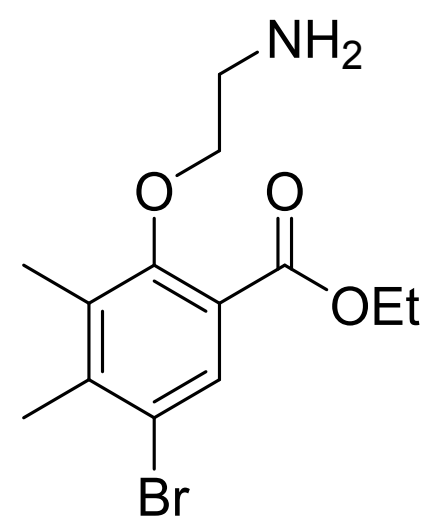

**16**

To a solution of crude ethyl 5-bromo-2-(2-((tert-butoxycarbonyl)amino)ethoxy)-3,4-dimethylbenzoate (185.6 g) in 1,4-dioxane (175 mL) was added a solution of hydrochloric acid (4M in 1,4-dioxane, 195 mL). After 6 h, another 195 mL of 4M HCl in 1,4-dioxane was added. This step was repeated after another 6 h until the starting material was consumed. The precipitate was collected using vacuum filtration and washed with cold 1,4-dioxane to remove triphenylphosphine oxide. The solid was further dried under vacuum to provide ethyl 2-(2-aminoethoxy)-5-bromo-3,4-dimethylbenzoate (57.2 g) as a white powder which was taken as an HCl salt to the next step without further purification.

The procedure was repeated for another batch of ethyl 5-bromo-2-(2-((tert-butoxycarbonyl)amino)ethoxy)-3,4-dimethylbenzoate (219.4 g) to obtain 62.1 g of the corresponding HCl salt which was combined with 57.2 g and used in the next step. HRMS (ESI/Q-TOF) m/z: $[M+H]^+$ Calcd for $C_{13}H_{18}BrNO_3$ 316.0543, Found 316.0543.

**Step e: Preparation of 7-bromo-8,9-dimethyl-3,4-dihydrobenzo[*f*][1,4]oxazepin-5(2*H*)-one (11)**

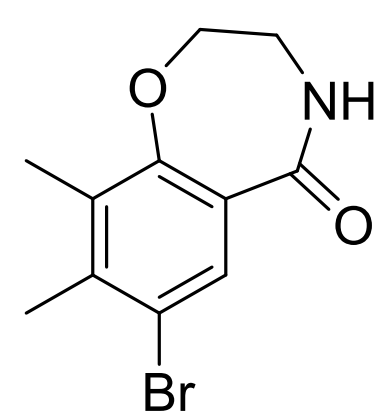

**17**

A solution of crude methyl 2-(2-aminoethoxy)-5-bromo-3,4-dimethylbenzoate hydrochloride salt (119.3 g, 377 mmol) in ethanol (1.5 L) was added to potassium carbonate (101.04 g, 731 mmol). The resulting solution was heated at 80 °C for 18 h and concentrated under reduced pressure until dryness. The off-white solid was suspended in water and sonicated to dissolve the unreacted $K_2CO_3$ and KCl. The precipitate was collected by vacuum filtration and the solid was washed with

water and dried under vacuum. The resulting powder was redissolved in DCM and passed through a short pad of silica which was thoroughly washed with DCM (500-750 mL) followed by 10% MeOH/DCM 500 mL). The combined organic fractions were concentrated under reduced pressure to provide the title compound (30 g, 76% yield over three steps) as an off-white solid. $^{1}$H NMR (400 MHz, $CDCl_3$) δ 7.90 (s, 1H), 6.91 (bs, 1H), 4.34 (t, $J$ = 5.2 Hz, 2H), 3.42 (q, $J$ = 5.4 Hz, 2H), 2.41 (s, 3H), 2.27 (s, 3H); $^{13}$C NMR (101 MHz, $CDCl_3$) δ 170.3, 151.8, 141.8, 131.5, 131.4, 125.2, 119.8, 73.0, 41.0, 20.6, 13.7; HRMS (ESI/Q-TOF) m/z: $[M+H]^+$ Calcd for $C_{11}H_{12}BrNO_2$ 270.0127, Found 270.0126.

**Step f: Preparation of 8,9-dimethyl-7-(4,4,5,5-tetramethyl-1,3,2-dioxaborolan-2-yl)-3,4-dihydrobenzo[*f*][1,4]oxazepin-5(2*H*)-one (18)**

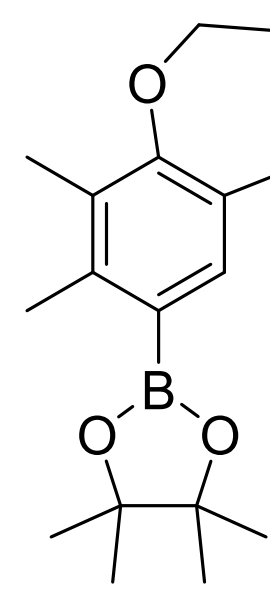

**18**

To a solution of 7-bromo-8,9-dimethyl-3,4-dihydrobenzo[*f*][1,4]oxazepin-5(2*H*)-one (20.0 g, 74.0 mmol) in anhydrous 1,4-dioxane (370 mL), bis(pinacolato)diboron (37.6 g, 148.1 mmol) and KOAc (21.8 g, 222.1 mmol) were added followed by Pd(dppf)$Cl_2$ (5.43 g, 7.40 mmol). The reaction mixture was evacuated and purged with $N_2$ (3x) and the reaction mixture was heated to 90 °C. Work-up procedure: After 6 h, the mixture was diluted with DCM and sat. soln. $NaHCO_3$. Layers were separated. Aqueous layer was extracted with DCM (3x). The combined extracts were concentrated. The crude material was re-dissolved in DCM and precipitated with the addition of hexanes. The precipitate was collected by vacuum filtration to provide the title compound (20.8 g, 88 % yield) as a dark gray solid which was used in the next step without further purification. $^{1}$H NMR (400 MHz, $CDCl_3$) δ 8.12 (s, 1H), 6.51 (m, 1H), 4.34 (t, $J$ = 4.8 Hz, 2H), 3.44 – 3.34 (m, 2H), 2.51 (s, 3H), 2.20 (s, 3H), 1.33 (s, 12H); ES-MS $[M+H]^+$ = 318.2.

Alternative work-up and purification procedure: After 6 h, the mixture was diluted with EtOAc/DCM and filtered through a pad of Celite. The filtrate was concentrated under reduced

pressure. Purification using normal phase chromatography on silica gel (0-70% EtOAc/DCM) to provide the title as a white powder. ES-MS $[M+H]^+ = 318.1$.

**Step g: Preparation of 8,9-dimethyl-7-(4-(oxazol-4-yl)benzyl)-3,4-dihydrobenzo[*f*][1,4]oxazepin-5(2*H*)-one (11, VU606069863)**

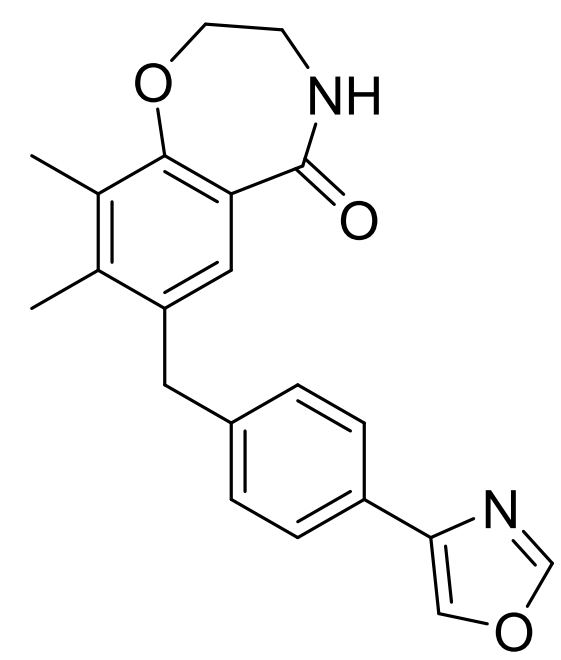

**11**, VU6069863

In a 100 mL RBF, 8,9-dimethyl-7-(4,4,5,5-tetramethyl-1,3,2-dioxaborolan-2-yl)-3,4-dihydrobenzo[*f*][1,4]oxazepin-5(2*H*)-one (4.08 g, 12.86 mmol), 4-(4-(chloromethyl)phenyl)oxazole (2.74 g, 14.15 mmol), $Cs_2CO_3$ (12.65 g, 38.59 mmol), Pd(dppf)$Cl_2$ (0.94 g, 1.29 mmol) were combined. Anhydrous 1,4-dioxane (67 mL) was added followed by water (13 mL). The reaction mixture was evacuated and purged with $N_2$ (3x) and heated to 105 °C. After 5 h, the reaction mixture was cooled to rt, and filtered through a pad of Celite which was washed thoroughly with DCM. The filtrate was concentrated under reduced pressure. The crude material was redissolved in DCM, washed with water (2x), dried ($MgSO_4$), filtered and concentrated. Purification using normal phase chromatography (0-3% MeOH/DCM/0.05% $NH_4OH$). The sample was further purified using reverse phase chromatography (10-55% MeCN/Water/0.05% aq. $NH_4OH$) to give the title compound (3.67 g, 82% yield). $^1$H NMR (400 MHz, DMSO-$d_6$) δ 8.56 (d, $J$ = 1.0 Hz, 1H), 8.43 (d, $J$ = 1.0 Hz, 1H), 8.19 (t, $J$ = 5.5 Hz, 1H), 7.73 – 7.67 (m, 2H), 7.26 (s, 1H), 7.19 – 7.17 (m, 2H), 4.20 (t, $J$ = 5.3 Hz, 2H), 3.99 (s, 2H), 3.34 (s, 1H), 3.19 (q, $J$ = 5.4 Hz, 2H), 2.14 (s, 3H), 2.15 (s, 3H); $^{13}$C NMR (101 MHz, $CDCl_3$) δ 171.5, 151.5, 151.4, 141.6, 140.7, 140.5, 134.2, 133.6, 130.0, 129.7, 129.1 (2C), 128.6, 125.8 (2C), 123.6, 72.8, 41.1, 39.9, 16.7, 12.8; HRMS (ESI/Q-TOF) m/z: $[M+H]^+$ Calcd for $C_{21}H_{20}N_2O_3$ 349.1547, Found 349.1548.

**Step h: Preparation of 4-(4-bromophenyl)oxazole (20)**

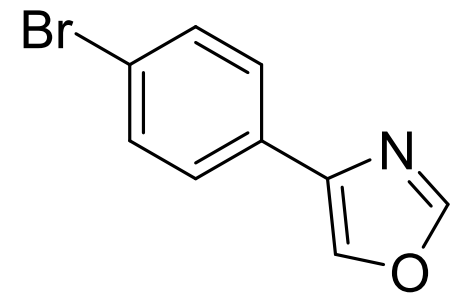


**20**

Ammonium formate (49.23 g, 780.7 mmol) was added portion-wise over 10 min to a solution of 2-bromo-1-(4-bromophenyl)ethan-1-one (62 g, 223.1 mmol) in formic acid (130 mL, 3445.9 mmol) at room temperature. The solution mixture was refluxed at 115 °C. After 2.5 h, the mixture was cooled to room temperature and concentrated *in vacuo*. The residue was dissolved in EtOAc and water. The organic layers were separated, and the aqueous layer was extracted with EtOAc (2x). The combined organics were dried ($MgSO_4$), filtered, and concentrated. The crude material was purified by normal phase chromatography on silica gel (0-15% EtOAc/hexanes) to afford the title compound (15.8 g, 32% yield). $^1H$ NMR (400 MHz, $CDCl_3$) δ 7.95 (d, $J$ = 0.9 Hz, 1H), 7.94 (s, 1H), 7.65 – 7.59 (m, 2H), 7.57 – 7.51 (m, 2H); $^{13}C$ NMR (101 MHz, DMSO-$d_6$) δ 152.8, 138.3, 135.7, 131.8 (2C), 130.0, 127.2 (2C), 121.0; HRMS (ESI/Q-TOF) m/z: $[M+H]^+$ Calcd for $C_9H_6BrNO$ 223.9706, Found 223.9708.

**Step i: Preparation of ethyl 4-(oxazol-4-yl)benzoate (21)**

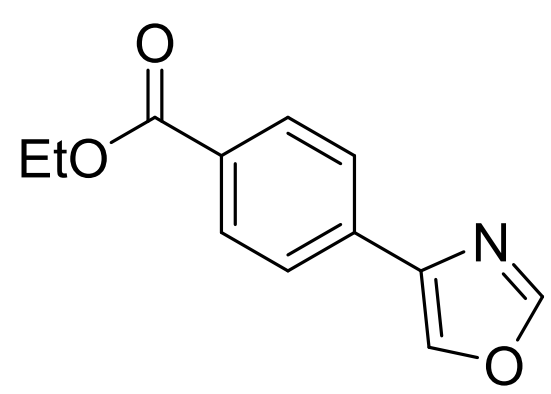


**21**

To a solution of 4-(4-bromophenyl)-1,3-oxazole (15.8 g, 70.5 mmol) was dissolved in ethanol (222 mL) and DMSO (113 mL) was added triethylamine (92.3 mL, 705.2 mmol). Under nitrogen atm, palladium(II) acetate (3.19 g, 14.1 mmol) was added followed by 1,3-bis(diphenylphosphino)propane (5.82 g, 14.1 mmol). The mixture was purged thoroughly with CO (g) (1 atm, balloon) and stirred at 75 °C for 16 h. The mixture was cooled to room temperature, diluted with MeOH, filtered over Celite and concentrated under reduced pressure to remove methanol. The remaining material in DMSO was diluted with EtOAc (800 mL) and washed with water (3 x 50mL) and brine (2 x 50mL). The organic layer was dried ($MgSO_4$), filtered and concentrated. Purification using normal phase chromatography on silica gel (0-25%

EtOAc/hexanes) to provide the title compound as a pale yellow crystalline solid (13.5 g, 88% yield). $^{1}H$ NMR (400 MHz, $CDCl_3$) δ 8.11 – 8.06 (m, 2H), 8.04 (d, *J* = 0.9 Hz, 1H), 7.97 (d, *J* = 0.9 Hz, 1H), 7.86 – 7.79 (m, 2H), 4.39 (q, *J* = 7.1 Hz, 2H), 1.41 (t, *J* = 7.1 Hz, 3H); $^{13}C$ NMR (101 MHz, DMSO-$d_6$) δ 165.4, 153.0, 138.4, 136.8, 135.2, 129.8 (2C), 129.1, 125.3 (2C), 60.8, 34.2; HRMS (ESI/Q-TOF) m/z: $[M+H]^+$ Calcd for $C_{12}H_{11}NO_3$ 218.0812, Found 218.0812.

**Step j: Preparation of (4-(oxazol-4-yl)phenyl)methanol (22)**

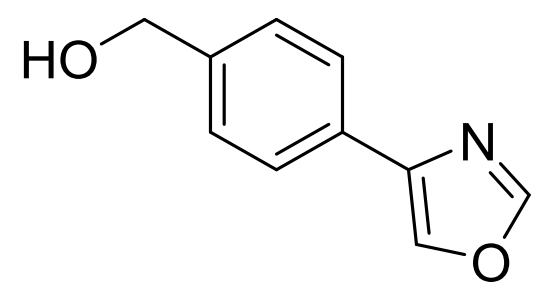

**22**

Under nitrogen, a solution of lithium aluminum hydride (2M in THF, 100 mL, 200 mmol) was added via cannula to anhydrous THF (55.9 mL) at 0 °C. The mixture was stirred for 5 min and a solution of ethyl 4-(1,3-oxazol-4-yl)benzoate (13.49 g, 62.1 mmol) in THF (254.6 mL) was added dropwise via a cannula. The resulting mixture was allowed to stir at 0 ℃ for 1 h. Upon completion, the reaction was worked up using Fieser procedure: at 0 °C to the reaction mixture, water (7.59 mL) was added slowly (caution: strong exotherm) followed by 15% NaOH aq. solution (7.59 mL) and water (22.8 mL). The thick resulting suspension was stirred with $MgSO_4$ vigorously at rt for 2 h and filtered through a thin pad of Celite which was rinsed thoroughly with EtOAc (200 mL), DCM (100 mL) then 10% MeOH/DCM (200 mL). The filtrate was concentrated under reduced pressure. Purification was completed using normal phase chromatography on silica gel (0-10% MeOH/DCM) to afford the title compound (9.05 g, 83% yield). $^{1}H$ NMR (400 MHz, DMSO-$d_6$) δ 8.59 (d, *J* = 0.9 Hz, 1H), 8.45 (d, *J* = 1.0 Hz, 1H), 7.78 – 7.72 (m, 2H), 7.39 – 7.36 (m, 2H), 5.23 (t, *J* = 5.7 Hz, 1H), 4.52 (d, *J* = 5.7 Hz, 2H); $^{13}C$ NMR (101 MHz, DMSO-$d_6$) δ 152.6, 142.4, 139.2, 134.8, 129.1, 126.8 (2C), 125.0 (2C), 62.65; HRMS (ESI/Q-TOF) m/z: $[M+H]^+$ Calcd for $C_{10}H_9NO_2$ 176.0706, Found 176.0709.

**Step k: Preparation of 4-(4-(chloromethyl)phenyl)oxazole (23)**

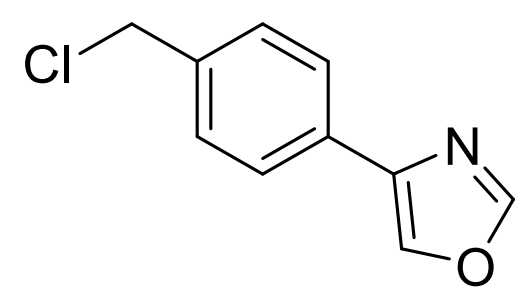

**23**

To a clear solution of (4-(oxazol-4-yl)phenyl)methanol (5.07 g, 28.9 mmol) in DCM (186 mL) at 0 °C was added thionyl chloride (10.51 mL, 144.7 mmol) dropwise using an addition funnel. The white suspension mixture was stirred at rt for 1 h and concentrated under reduced pressure to afford the title compound (5.41 g, 97% yield) which was carried forward without purification. $^1$H NMR (400 MHz, $CDCl_3$) δ 7.97 (d, $J$ = 0.9 Hz, 1H), 7.96 (d, $J$ = 1.0 Hz, 1H), 7.79 – 7.71 (m, 2H), 7.48 – 7.40 (m, 2H), 4.61 (s, 2H); $^{13}$C NMR (101 MHz, DMSO-$d_6$) δ 152.7, 138.8, 137.3, 135.5, 130.7, 129.4 (2C), 125.4 (2C), 46.0; HRMS (ESI/Q-TOF) m/z: $[M+H]^+$ Calcd for $C_{10}H_8ClNO$ 194.0367, Found 194.0365.

**Scheme S1: Synthesis of M364a (24)**

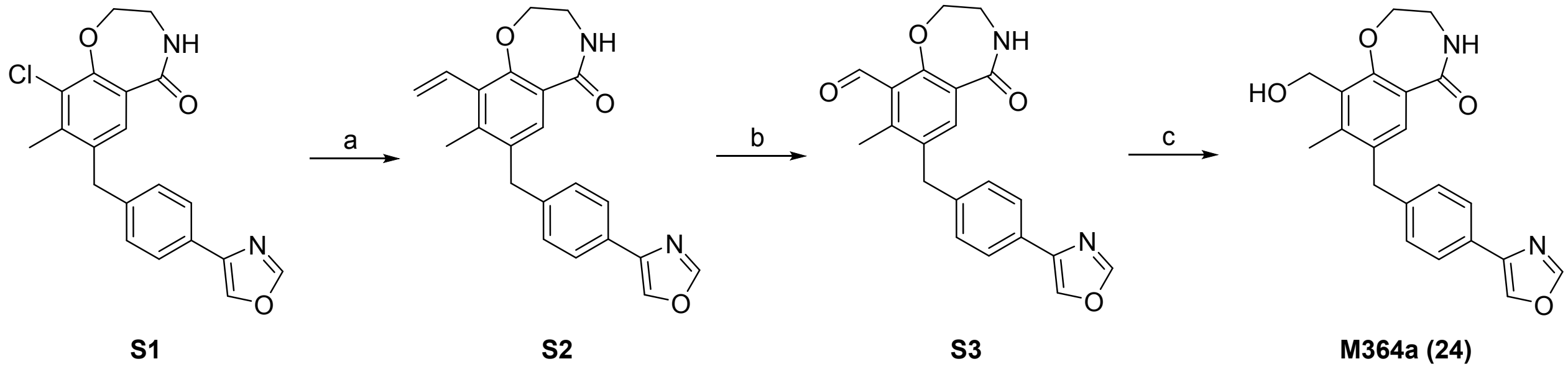


$^a$Reagents and conditions: (a) Potassium vinyltrifluoroborate, RuPhos-G3-Pd, $K_2CO_3$, TFA/$H_2O$, microwave, 140 °C, 30 min, 83%; (b) $OsO_4$, NMO, THF/$H_2O$, 0 °C – rt, 1 h, then $NaIO_4$, rt, 1 h; (c) $NaBH_4$, MeOH, 0 °C – rt, 1 h, 80% over three steps.

**Step a: Preparation of 8-methyl-7-(4-(oxazol-4-yl)benzyl)-9-vinyl-3,4-dihydrobenzo[*f*][1,4]oxazepin-5(2*H*)-one (S2)**

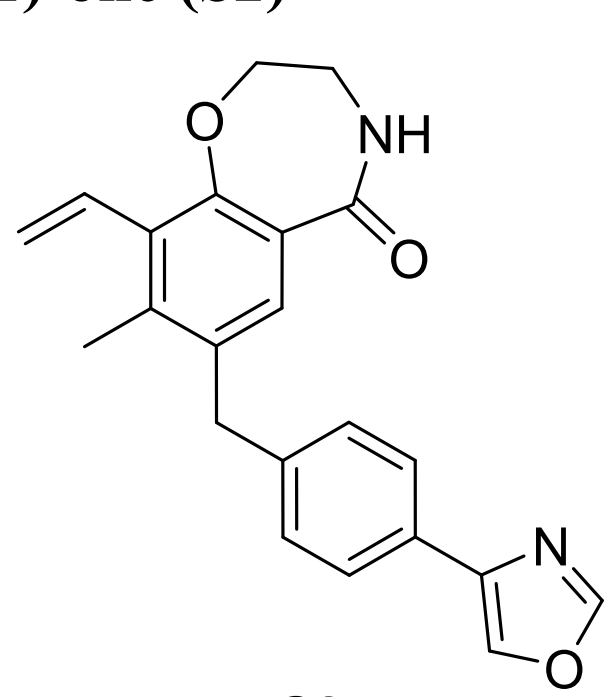


**S2**

To a solution of 9-chloro-8-methyl-7-(4-(oxazol-4-yl)benzyl)-3,4-dihydrobenzo[*f*][1,4]oxazepin-5(2*H*)-one (117.0 mg, 0.32 mmol) in THF (2.7 mL) and $H_2O$ (0.8 mL) was added potassium carbonate (133.5 mg, 0.95 mmol), potassium vinyltrifluoroborate (85 mg, 0.63 mmol) and

RuPhos-G3-Palladacycle (26.6 mg, 0.03 mmol). The reaction mixture was subjected to a microwave reactor for 15 min at 140 ℃. Upon completion, the mixture was diluted with DCM and $H_2O$. Layers were separated. Aqueous layer was extracted with DCM (3x). The combined extracts were concentrated under reduced pressure. Purification using normal phase chromatography on silica gel (0-10% MeOH/DCM) to provide the title compound (106 mg, 83% yield). $^1$H NMR (400 MHz, DMSO-$d_6$) δ 8.57 (d, $J$ = 1.0 Hz, 1H), 8.44 (d, $J$ = 1.0 Hz, 1H), 8.24 (t, $J$ = 5.6 Hz, 1H), 7.75 – 7.67 (m, 2H), 7.33 (s, 1H), 7.23 – 7.17 (m, 2H), 6.71 (dd, $J$ = 17.9, 11.6 Hz, 1H), 5.54 (dd, $J$ = 11.6, 2.1 Hz, 1H), 5.43 (dd, $J$ = 17.9, 2.1 Hz, 1H), 4.17 (t, $J$ = 5.3 Hz, 2H), 4.01 (s, 2H), 3.22 (q, $J$ = 5.7 Hz, 2H), 2.21 (s, 3H); ES-MS $[M+H]^+$ = 361.3.

**Step b: Preparation of 8-methyl-7-(4-(oxazol-4-yl)benzyl)-5-oxo-2,3,4,5-tetrahydrobenzo[*f*][1,4]oxazepine-9-carbaldehyde (S3)**

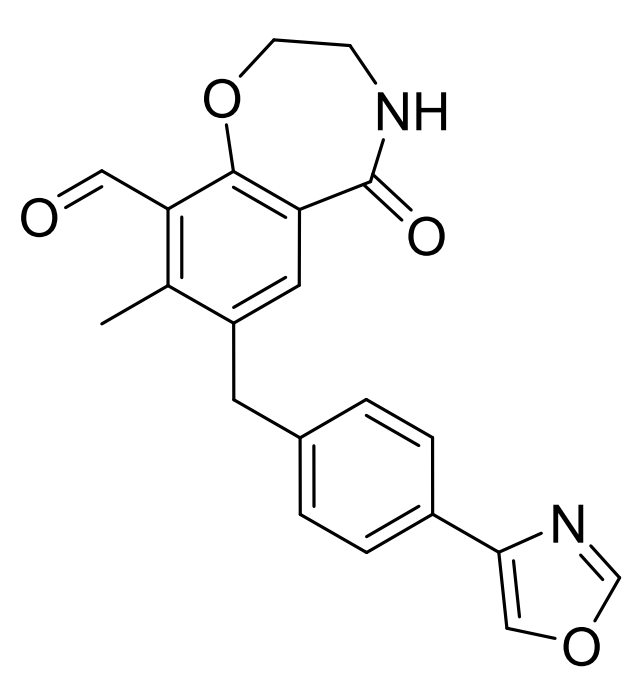

**S3**

To a solution of 8-methyl-7-(4-(oxazol-4-yl)benzyl)-9-vinyl-3,4-dihydrobenzo[*f*][1,4]oxazepin-5(2*H*)-one (98 mg, 0.27 mmol) in THF (0.9 mL) and $H_2O$ (0.9 mL) at 0 ℃ was added a solution of osmium tetraoxide (2.5% wt in *t*-BuOH, 0.34 mL, 0.03 mmol) and 4-methylmorpholine *N*-oxide (38.2 mg, 0.33 mmol). The resulting mixture was stirred at rt until the starting material was completely converted to the diol intermediate. At which point, sodium periodate (146 mg, 0.68 mmol) was added to the reaction mixture and allowed to stir for 16 h. Upon completion, the mixture was diluted with DCM and 10% aq. $Na_2S_2O_3$ solution. The layers were separated. The aqueous layer was extracted with DCM (3x). The combined extracts were washed with brine, dried over $Na_2SO_4$, filtered and concentrated to afford the title compound (98.4 mg) which was assumed quantitative yield and carried forward without further purification. $^1$H NMR (400 MHz, DMSO-$d_6$) δ 10.43 (s, 1H), 8.57 (d, $J$ = 1.0 Hz, 1H), 8.48 – 8.41 (m, 2H), 7.75 – 7.68 (m, 3H), 7.23 – 7.16 (m, 2H), 4.40 (t, $J$ = 5.3 Hz, 2H), 4.06 (s, 2H), 3.36 (d, $J$ = 5.3 Hz, 2H), 2.38 (s, 3H); ES-MS $[M+H]^+$ = 363.3.

**Step c: Preparation of 9-(hydroxymethyl)-8-methyl-7-(4-(oxazol-4-yl)benzyl)-3,4-dihydrobenzo[*f*][1,4]oxazepin-5(2*H*)-one (24)**

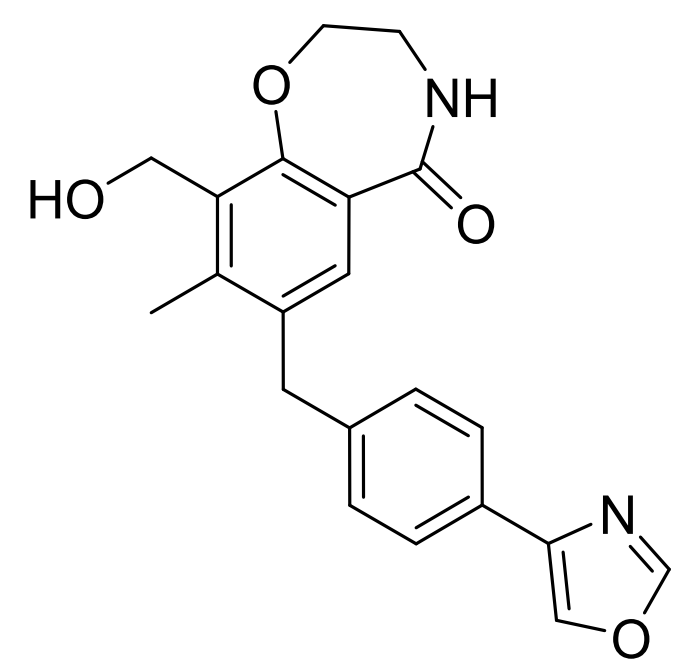


**M364a (24)**

To a solution of 8-methyl-7-(4-(oxazol-4-yl)benzyl)-5-oxo-2,3,4,5-tetrahydrobenzo[*f*][1,4]oxazepine-9-carbaldehyde (95.0 mg, 0.26 mmol) in methanol (2.6 mL) at 0 ℃ was added sodium borohydride (20 mg, 0.52 mmol). The reaction mixture was allowed to stir at rt for 1 h and concentrated to dryness under reduced pressure. Purification using normal phase chromatography on silica gel (0-10% MeOH/DCM) followed by RP-HPLC (5-95% MeCN/0.1% aq. TFA). After work-up with sat. soln. $NaHCO_3$ and extraction with iPA/$CHCl_3$ (1:3 v/v), the title compound was obtained as an off-white powder. (76 mg, 80% yield). $^1$H NMR (400 MHz, DMSO-$d_6$) δ 8.56 (d, $J$ = 1.0 Hz, 1H), 8.43 (d, $J$ = 1.0 Hz, 1H), 8.21 (t, $J$ = 5.5 Hz, 1H), 7.75 – 7.67 (m, 2H), 7.32 (s, 1H), 7.23 – 7.17 (m, 2H), 4.75 (t, $J$ = 5.2 Hz, 1H), 4.54 (d, $J$ = 5.3 Hz, 2H), 4.22 (t, $J$ = 5.4 Hz, 2H), 4.00 (s, 2H), 3.23 – 3.14 (m, 2H), 2.28 (s, 3H); HRMS (ESI/Q-TOF) m/z: $[M+H]^+$ Calcd for $C_{21}H_{20}N_2O_4$ 365.1496, Found 365.1500.

**Scheme S2: Synthesis of M364b (25)**

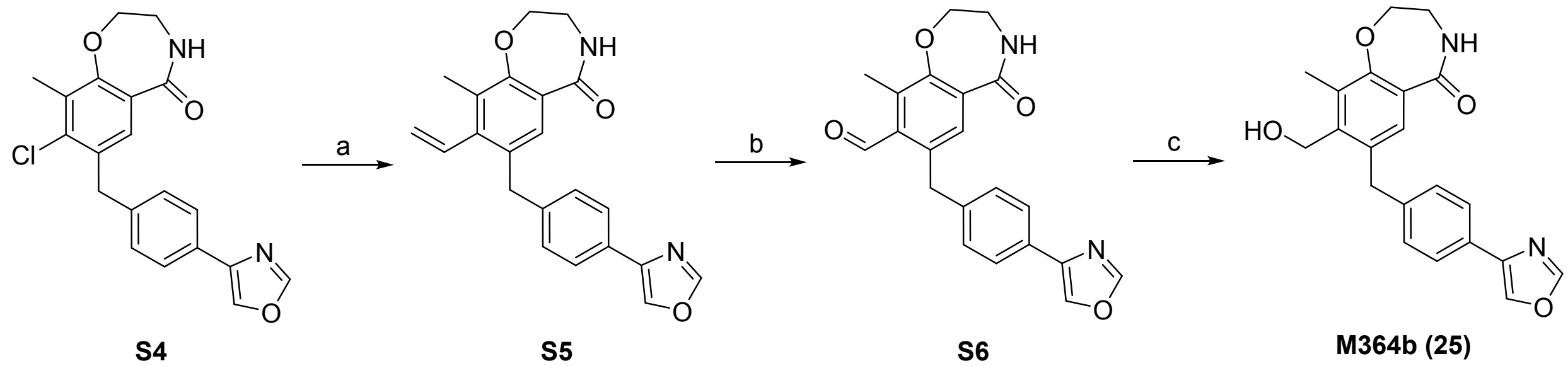


$^a$Reagents and conditions: (a) Potassium vinyltrifluoroborate, RuPhos-G3-Pd, $K_2CO_3$, TFA/$H_2O$, microwave, 140 °C, 30 min, 82%; (b) $OsO_4$, NMO, THF/$H_2O$, 0 °C – rt, 1 h, then $NaIO_4$, rt, 1 h; (c) $NaBH_4$, MeOH, 0 °C – rt, 1 h, 49% over three steps.

**Step a: Preparation of 9-methyl-7-(4-(oxazol-4-yl)benzyl)-8-vinyl-3,4-dihydrobenzo[*f*][1,4]oxazepin-5(2*H*)-one**

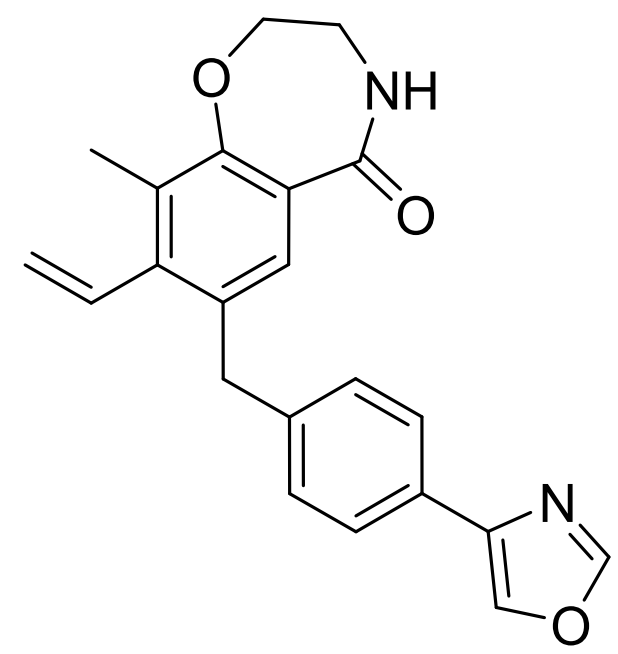

**S5**

To a solution of 8-chloro-9-methyl-7-(4-(oxazol-4-yl)benzyl)-3,4-dihydrobenzo[*f*][1,4]oxazepin-5(2*H*)-one (117 mg, 0.32 mmol) in THF (2.7 mL) and $H_2O$ (0.8 mL) was added potassium carbonate (133.5 mg, 0.95 mmol), potassium vinyltrifluoroborate (85 mg, 0.63 mmol) and RuPhos-G3-Palladacycle (26.6 mg, 0.03 mmol). The reaction mixture was subjected to a microwave reactor for 15 min at 140 °C. Upon completion, the mixture was diluted with DCM and $H_2O$. Layers were separated. Aqueous layer was extracted with DCM (3x). The combined extracts were concentrated under reduced pressure. Purification using normal phase chromatography on silica gel (0-10% MeOH/DCM) to provide the title compound (93.7 mg, 82% yield). $^1$H NMR (400 MHz, DMSO-$d_6$) δ 8.56 (d, $J$ = 1.0 Hz, 1H), 8.43 (d, $J$ = 1.0 Hz, 1H), 8.24 (t, $J$ = 5.5 Hz, 1H), 7.73 – 7.65 (m, 2H), 7.25 (s, 1H), 7.20 – 7.13 (m, 2H), 6.72 (dd, $J$ = 18.0, 11.6 Hz, 1H), 5.59 (dd, $J$ = 11.6, 1.9 Hz, 1H), 5.23 (dd, $J$ = 18.0, 1.9 Hz, 1H), 4.22 (t, $J$ = 5.3 Hz, 2H), 3.99 (s, 2H), 3.21 (q, $J$ = 5.4 Hz, 2H), 2.18 (s, 3H); ES-MS $[M+H]^+$ = 361.3.

**Step b: Preparation of 9-methyl-7-(4-(oxazol-4-yl)benzyl)-5-oxo-2,3,4,5-tetrahydrobenzo[*f*][1,4]oxazepine-8-carbaldehyde**

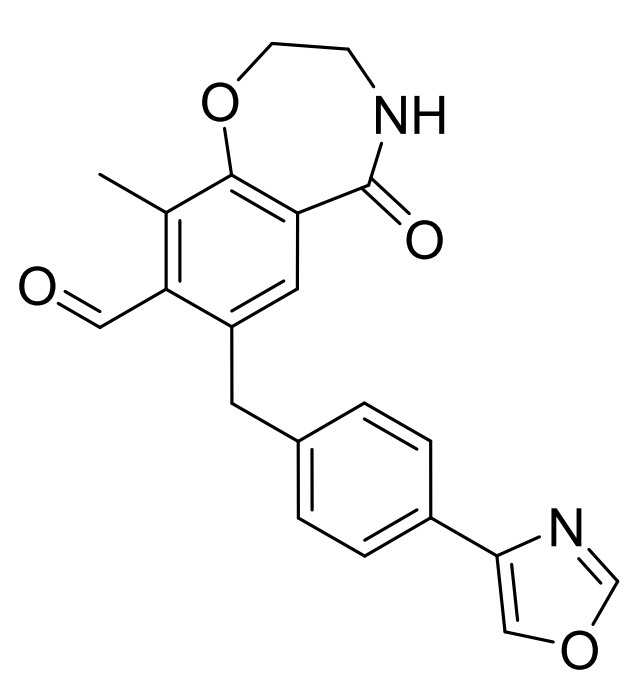

**S6**

To a solution of 9-methyl-7-(4-(oxazol-4-yl)benzyl)-8-vinyl-3,4-dihydrobenzo[*f*][1,4]oxazepin-5(2*H*)-one (93 mg, 0.26 mmol) in THF (0.9 mL) and $H_2O$ (0.9 mL) at 0 ℃ was added a solution of osmium tetraoxide (2.5% wt in *t*-BuOH, 0.32 mL, 0.03 mmol) and 4-methylmorpholine *N*-oxide (36.3 mg, 0.31 mmol). The resulting mixture was stirred at rt until the starting material was completely converted to the diol intermediate. At which point, sodium periodate (138.6 mg, 0.65 mmol) was added to the reaction mixture. After 1 h, the reaction mixture was diluted with DCM and 10% aq. $Na_2S_2O_3$ solution. The layers were separated. The aqueous layer was extracted with DCM (3x). The combined extracts were washed with brine, dried over $Na_2SO_4$, filtered and concentrated to afford (100 mg) which was assumed quantitative yield and carried forward without further purification. ES-MS $[M+H]^+$ = 363.3.

**Step c: Preparation of 8-(hydroxymethyl)-9-methyl-7-(4-(oxazol-4-yl)benzyl)-3,4-dihydrobenzo[*f*][1,4]oxazepin-5(2*H*)-one**

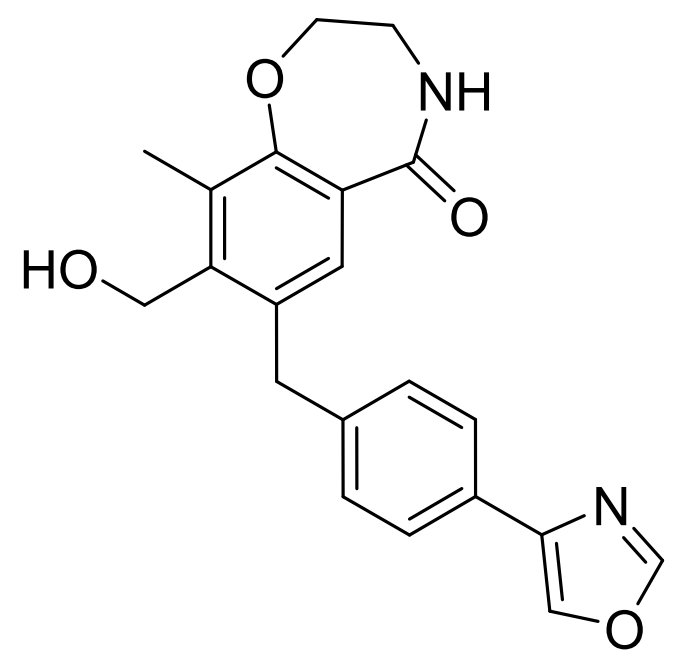


**M364b (25)**

To a solution of 9-methyl-7-(4-(oxazol-4-yl)benzyl)-5-oxo-2,3,4,5-tetrahydrobenzo[*f*][1,4]oxazepine-8-carbaldehyde (100 mg, 0.27 mmol) in methanol (2.6 mL) at 0 ℃ was added sodium borohydride (21 mg, 0.55 mmol). The reaction mixture was allowed to stir at rt for 1 h and concentrated to dryness under reduced pressure. Purification using normal phase chromatography on silica gel (0-10% MeOH/DCM) followed by RP-HPLC (5-95% MeCN/0.1% aq. TFA). After work-up with sat. soln. $NaHCO_3$ and extraction with iPA/$CHCl_3$ (1:3, v/v), the title compound was obtained as an off-white powder. (48 mg, 48% yield). $^1H$ NMR (400 MHz, DMSO-$d_6$) δ 8.56 (d, $J$ = 1.0 Hz, 1H), 8.43 (d, $J$ = 1.0 Hz, 1H), 8.23 (t, $J$ = 5.6 Hz, 1H), 7.74 – 7.66 (m, 2H), 7.21 – 7.19 (m, 3H), 4.97 (t, $J$ = 5.1 Hz, 1H), 4.50 (d, $J$ = 5.1 Hz, 2H), 4.20 (t, $J$ = 5.3 Hz, 2H), 4.11 (s, 2H), 3.21 – 3.14 (m, 2H), 2.28 (s, 3H); HRMS (ESI/Q-TOF) m/z: $[M+H]^+$ Calcd for $C_{21}H_{20}N_2O_4$ 365.1496, Found 365.1498.

**Scheme S3: Synthesis of M380a (26)**

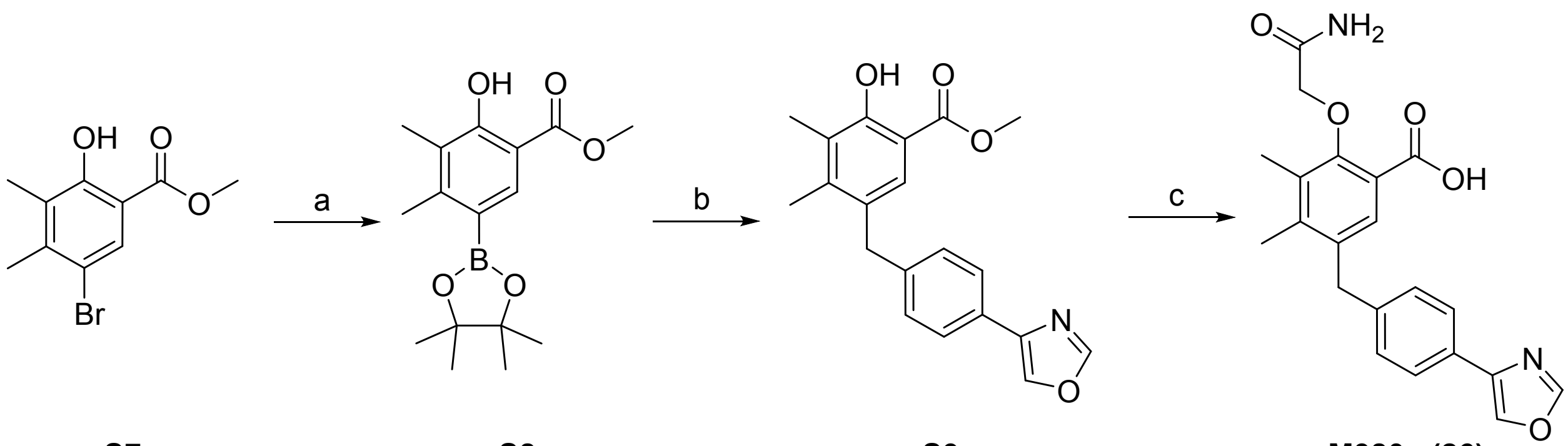


S7 S8 S9 M380a (26)

[a]Reagents and conditions: (a) Bis(pinacolato)diboron, KOAc, Pd(dppf)$Cl_2$, toluene, 100 °C; (b) **23**, $Cs_2CO_3$, Pd(dppf)$Cl_2$, 1,4-dioxane/$H_2O$, 100 °C, 64% over two steps; (c) 2-bromoacetamide, NaH (60% in mineral oil), DMF, 0 °C – rt, 1 h then LiOH, rt, 5%.

**Step a: Preparation of methyl 2-hydroxy-3,4-dimethyl-5-(4,4,5,5-tetramethyl-1,3,2-dioxaborolan-2-yl)benzoate (S8)**

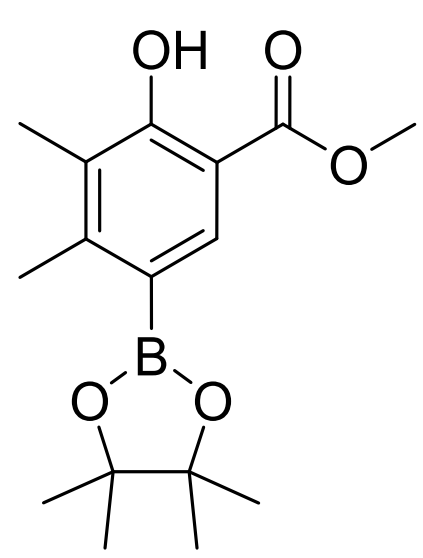


**S8**

To a solution of methyl 5-bromo-2-hydroxy-3,4-dimethylbenzoate (200 mg, 0.77 mmol) in toluene (5.5 mL), bis(pinacolato)diboron (294 mg, 1.16 mmol) and KOAc (227.3 g, 2.32 mmol) were added followed by Pd(dppf)$Cl_2$ (40.8 mg, 0.06 mmol). The reaction mixture was evacuated and purged with $N_2$ (3x) and the reaction mixture was heated to 110 °C. After 8 h, the mixture was filtered through a pad of Celite which was rinsed thoroughly with DCM and EtOAc. The filtrate was concentrated under reduced pressure to provide the title compound (507 mg) which was carried forward without purification.

**Step b: Preparation of methyl 2-hydroxy-3,4-dimethyl-5-(4-(oxazol-4-yl)benzyl)benzoate (S9)**

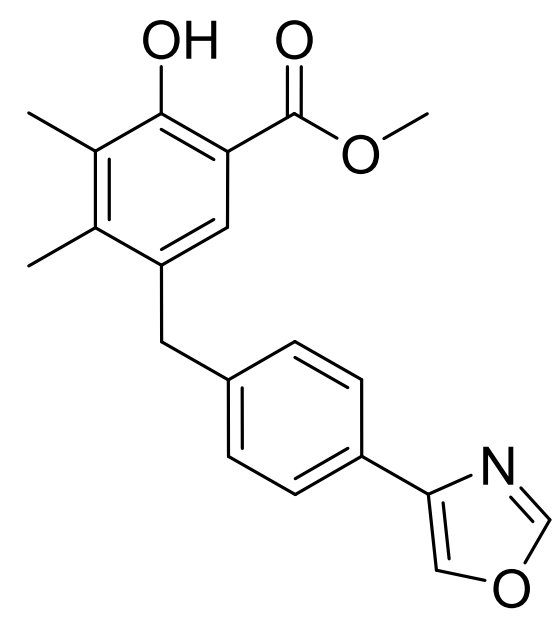

**S9**

To a solution of methyl 2-hydroxy-3,4-dimethyl-5-(4,4,5,5-tetramethyl-1,3,2-dioxaborolan-2-yl)benzoate (236 mg, 0.77 mmol) in anhydrous 1,4-dioxane (4.3 mL) and water (0.86 mL) was added 4-(4-(chloromethyl)phenyl)oxazole (195 mg, 0.85 mmol), $Cs_2CO_3$ (758 mg, 2.31 mmol), $Pd(dppf)Cl_2$ (56.6 mg, 0.08 mmol). The reaction mixture was evacuated and purged with $N_2$ (3x) and heated to 100 °C. After 5 h, the reaction mixture was cooled to rt, diluted with DCM and sat. soln. $NaHCO_3$. Layers were separated. Aqueous layer was extracted with DCM (3x). The combined extracts were concentrated and purified using normal phase chromatography (0-100% EtOAc/hexanes) to provide the title compound (165.5 mg, 64% yield over two steps. ES-MS $[M+H]^+ = 338.2$.

**Step c: 2-(2-amino-2-oxoethoxy)-3,4-dimethyl-5-(4-(oxazol-4-yl)benzyl)benzoic acid (M380a, 26)**

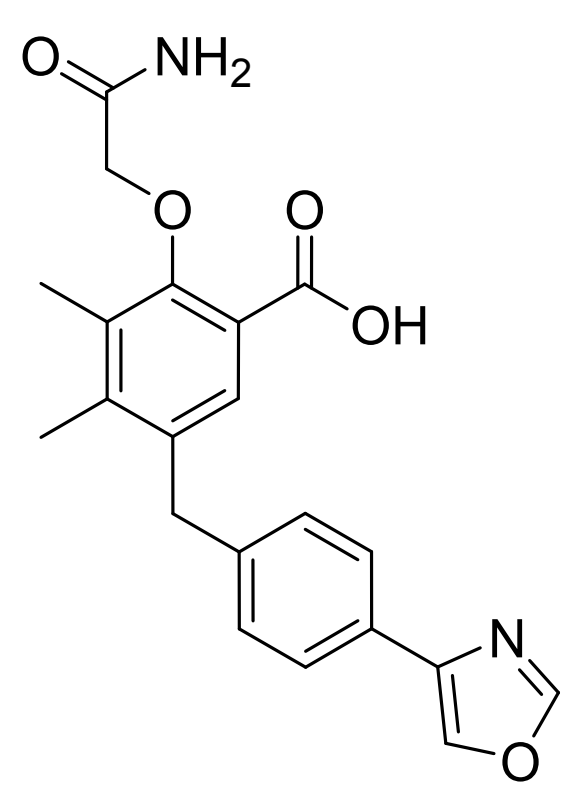

**M380a (26)**

To a solution of methyl 2-hydroxy-3,4-dimethyl-5-(4-(oxazol-4-yl)benzyl)benzoate (46 mg, 0.14 mmol) in DMF (0.68 mL) at 0 °C was added sodium hydride (60% in mineral oil, 6.4 mg, 0.16 mmol). The reaction mixture was stirred for 45 min and 2-bromoacetamide (37.6 mg, 0.28 mmol) was added dropwise. The reaction was allowed to stir at rt for 1 h or until the starting material was completely consumed. A solution of lithium hydroxide (1M, 0.68 mL, 0.68 mmol) was added.

After 1 h, the reaction mixture was neutralized with HCl (1M) and extracted with DCM (2x) and iPA/$CHCl_3$ (1:3, v/v. 1x). The combined extracts were concentrated and purified using normal phase chromatography on silica gel (0-10% MeOH/DCM). The material was further purified using RP-HPLC (5-95% MeCN/0.05 aq. $NH_4OH$). After concentration, the title compound was obtained as a white powder (2.5 mg, 5% yield). $^1$H NMR (400 MHz, MeOD) δ 8.25 (d, $J$ = 1.0 Hz, 1H), 8.22 (d, $J$ = 1.0 Hz, 1H), 7.68 – 7.65 (m, 2H), 7.54 (s, 1H), 7.18 – 7.15 (m, 2H), 4.32 (s, 2H), 4.06 (s, 2H), 2.66 (s, 3H), 2.25 (s, 3H), 2.19 (s, 3H); HRMS (ESI/Q-TOF) m/z: $[M+H]^+$ Calcd for $C_{21}H_{20}N_2O_5$ 381.1445, Found 381.1451.

**Scheme S4: Synthesis of M355 (27)**

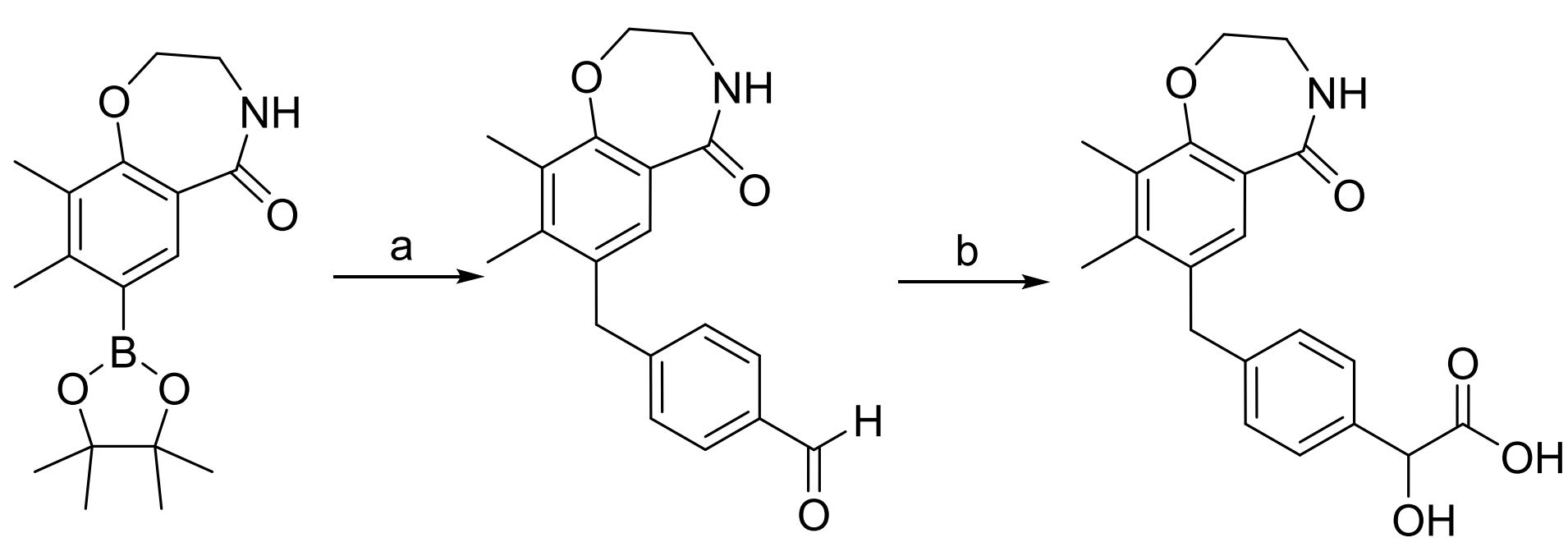


**18** **S10** **M355 (27)**

[a]Reagents and conditions: (a) 4-(bromomethyl)benzaldehyde, $Cs_2CO_3$, Pd(dppf)$Cl_2$, 1,4-dioxane/$H_2O$, 100 °C, 56%; (b) benzyltriethylammonium chloride, NaOH, $CHCl_3$ rt - 50 °C, 1 h , 19%.

**Step a: Synthesis of 4-((8,9-dimethyl-5-oxo-2,3,4,5-tetrahydrobenzo[*f*][1,4]oxazepin-7-yl)methyl)benzaldehyde (S10)**

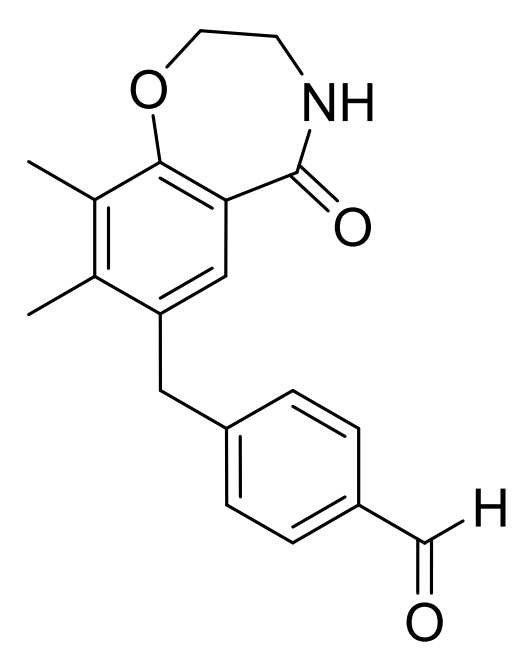


**S10**

To a solution of 7-bromo-8,9-dimethyl-3,4-dihydrobenzo[*f*][1,4]oxazepin-5(2*H*)-one (150 mg, 0.47 mmol) in anhydrous 1,4-dioxane (2.6 mL) and water (0.5 mL) was added 4-(bromomethyl)benzyladehyde (70 µL, 0.52 mmol), $Cs_2CO_3$ (461 mg, 1.42 mmol), Pd(dppf)$Cl_2$ (35 mg, 0.05 mmol). The reaction mixture was evacuated and purged with $N_2$ (3x) and heated to 100

°C. After 6 h, the reaction mixture was cooled to rt, diluted with DCM and sat. soln. $NaHCO_3$. Layers were separated. Aqueous layer was extracted with DCM (3x). The combined extracts were concentrated and purified using normal phase chromatography (0-100% EtOAc/hexanes then 0-10% MeOH/DCM) to provide the title compound (82 mg, 56% yield). ES-MS $[M+H]^+$ = 310.2.

**Step b: Synthesis of 2-(4-((8,9-dimethyl-5-oxo-2,3,4,5-tetrahydrobenzo[*f*][1,4]oxazepin-7-yl)methyl)phenyl)-2-hydroxyacetic acid (M355, 27)**

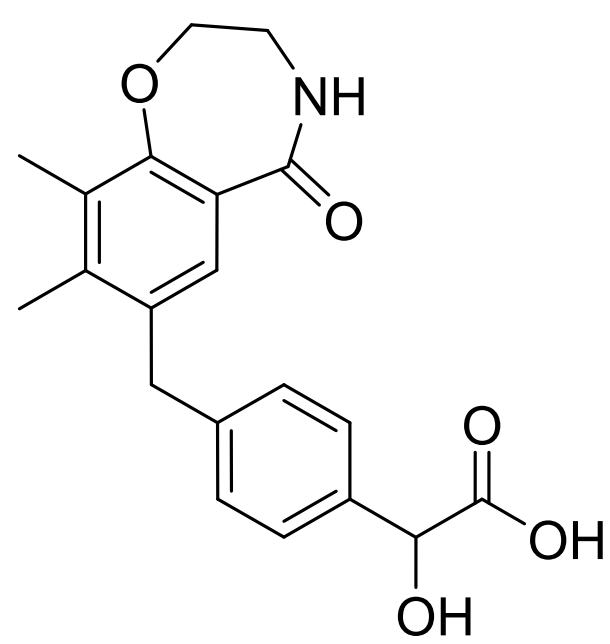

**M355 (27)**

To a solution of 4-((8,9-dimethyl-5-oxo-2,3,4,5-tetrahydrobenzo[*f*][1,4]oxazepin-7-yl)methyl)benzaldehyde (82 mg, 0.27 mmol) in chloroform (75 µL) was added benzyltriethylammonium chloride (31.4 mg, 0.14 mmol). The reaction mixture was stirred under nitrogen atmosphere at 50 °C and a solution of sodium hydroxide (12M, 70 µL, 0.83 mmol) was added dropwise over 1 h. The resulting mixture was allowed to stir at 50 °C until the starting material was completely consumed then diluted with DCM and 1M NaOH soln. The layers were separated. The aqueous layer was acidified with 1M HCl soln. and extracted with DCM (3x). The combined extracts were concentrated and purified using RP-HLPC (5-95% MeCN/0.1% aq. TFA). After concentration, the material was further purified using RP-HPLC (5-95% MeCN/0.05% aq. $NH_4OH$) to provide the title compound as a white powder (19.4 mg, 19% yield). $^1H$ NMR (400 MHz, DMSO-$d_6$) δ 8.17 (t, $J$ = 5.6 Hz, 1H), 7.29 – 7.21 (m, 3H), 7.02 – 6.96 (m, 2H), 4.50 (s, 1H), 4.18 (t, $J$ = 5.4 Hz, 2H), 3.92 (s, 2H), 3.20 – 3.16 (m, 2H), 2.54 (s, 1H), 2.13 (s, 3H), 2.13 (s, 3H), COOH proton not observable; HRMS (ESI/Q-TOF) m/z: $[M+H]^+$ Calcd for $C_{20}H_{21}NO_5$ 356.1492, Found 356.1496.

# NMR Spectra

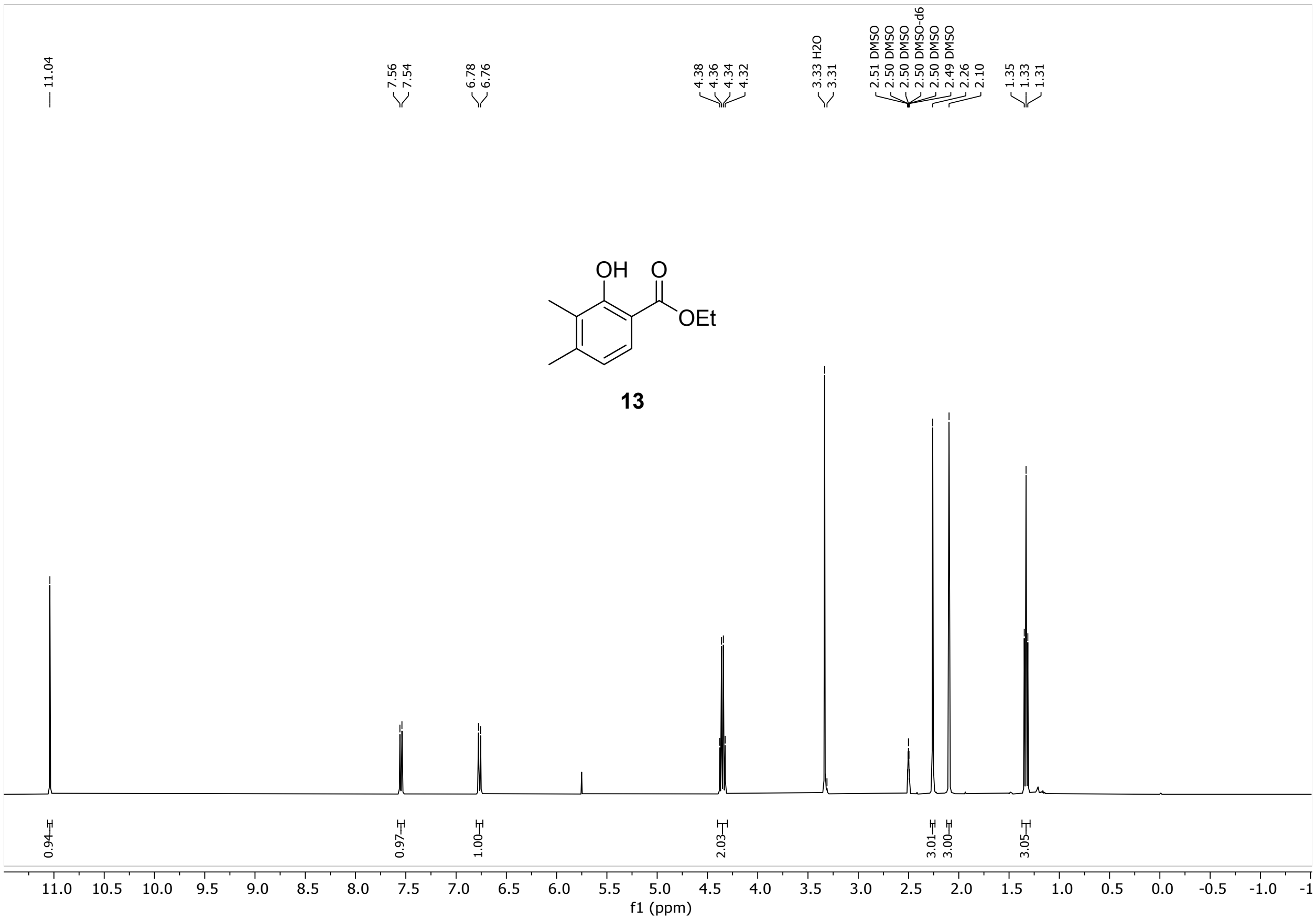


$^1$H NMR (400 MHz, DMSO-$d_6$) δ 11.04 (s, 1H), 7.55 (d, $J$ = 8.1 Hz, 1H), 6.77 (d, $J$ = 8.1 Hz, 1H), 4.35 (q, $J$ = 7.1 Hz, 2H), 2.26 (s, 3H), 2.10 (s, 3H), 1.33 (t, $J$ = 7.1 Hz, 3H).

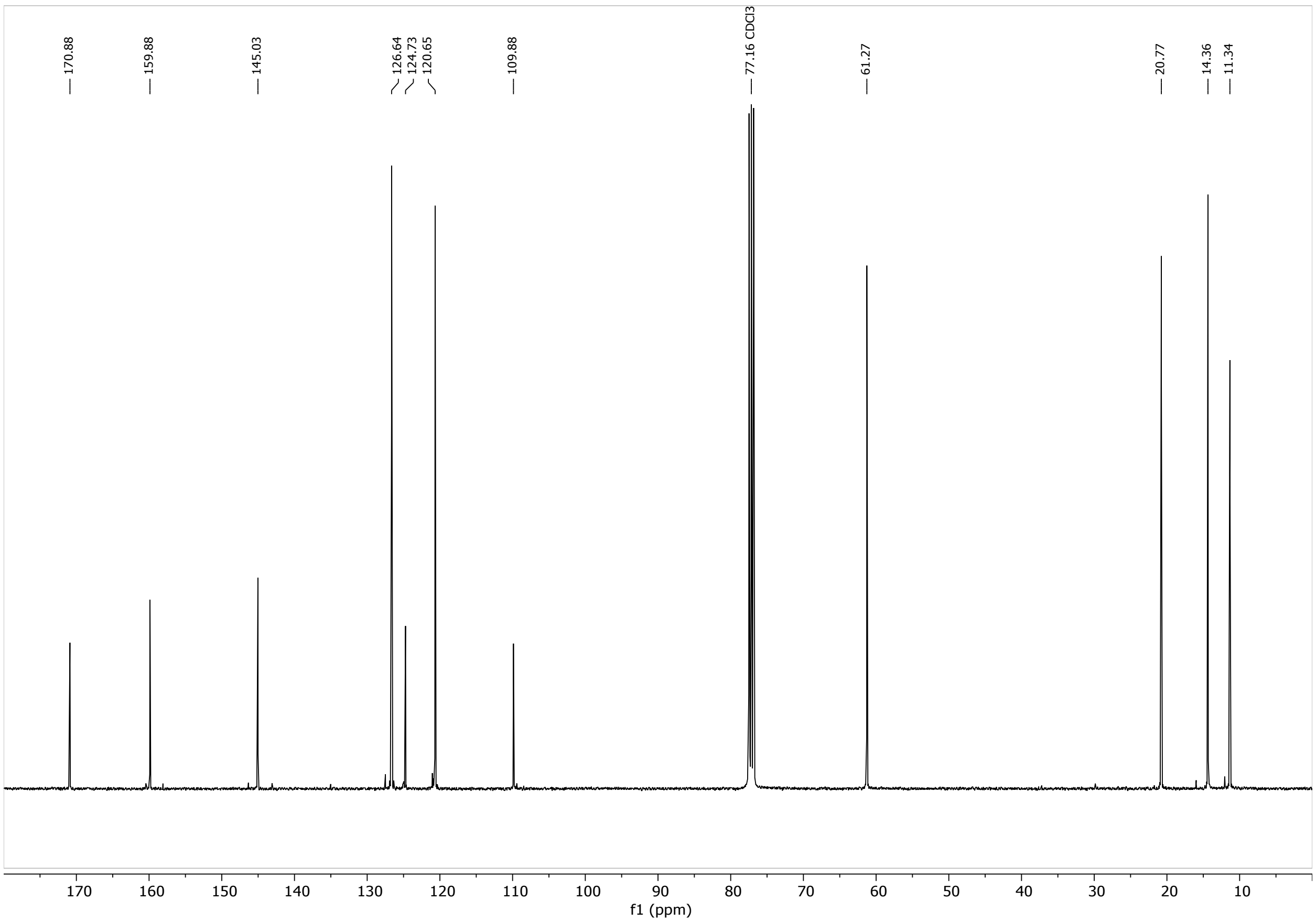


$^{13}C$ NMR (101 MHz, $CDCl_3$) δ 170.9, 159.9, 145.0, 126.6, 124.7, 120.7, 109.9, 61.3, 20.8, 14.4, 11.3

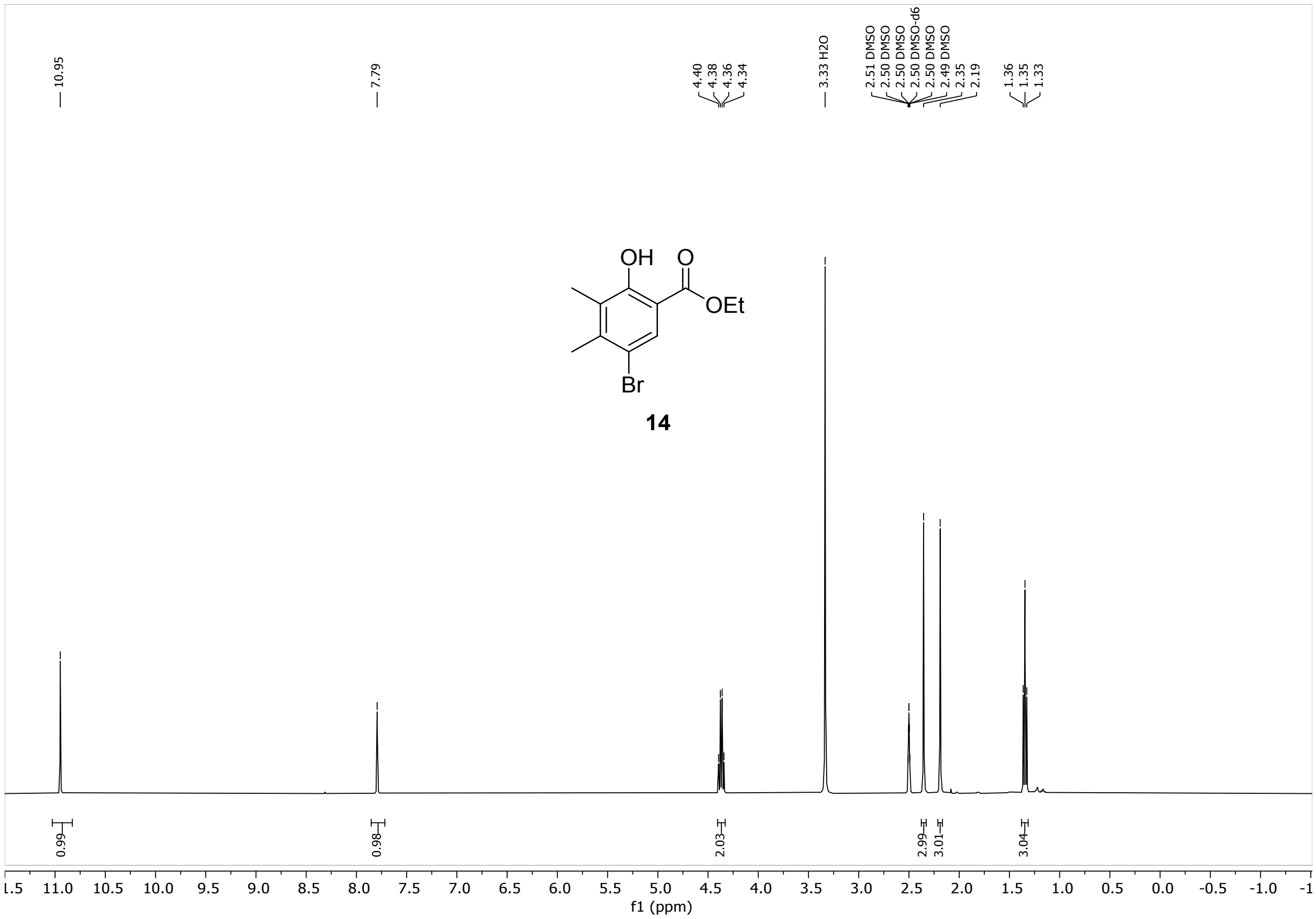

$^{1}H$ NMR (400 MHz, DMSO-$d_6$) 10.95 (s, 1H), 7.79 (s, 1H), 4.37 (q, $J$ = 7.1 Hz, 2H), 2.35 (s, 3H), 2.19 (s, 3H), 1.34 (t, $J$ = 7.1 Hz, 3H).

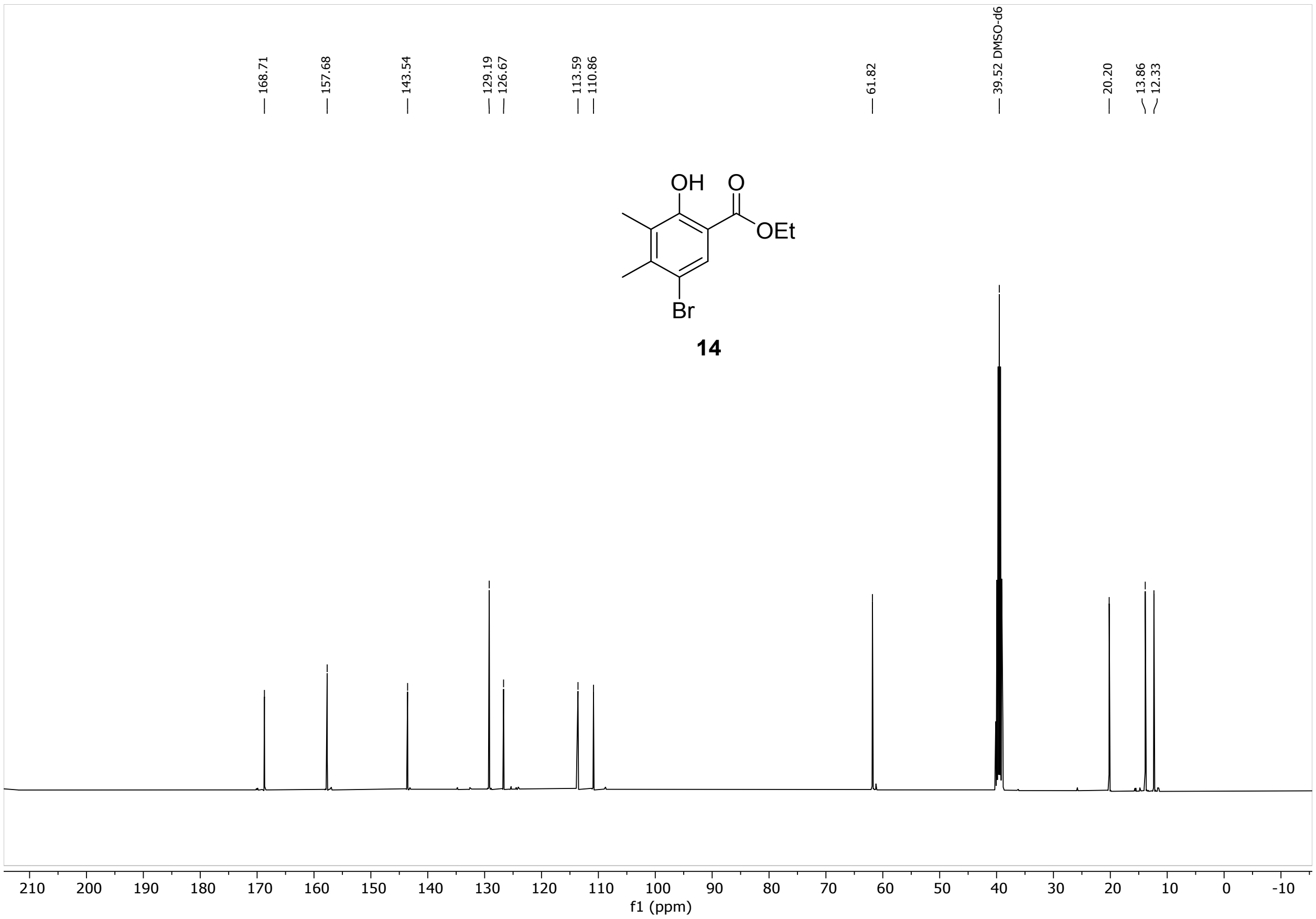


$^{13}C$ NMR (101 MHz, DMSO-$d_6$) δ 168.7, 157.7, 143.5, 129.2, 126.7, 113.6, 110.9, 61.8, 20.2, 13.9, 12.3.

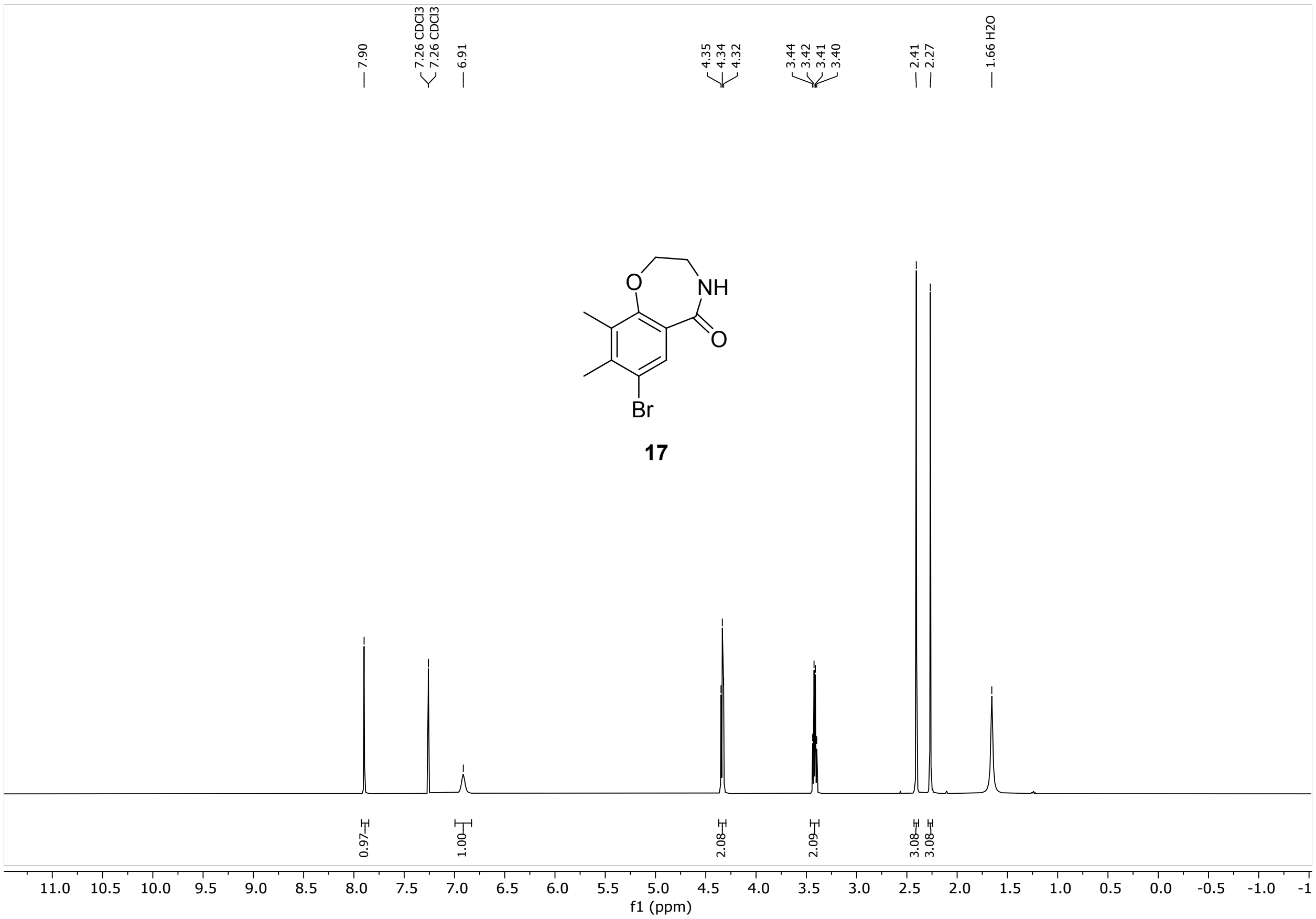


$^{1}$H NMR (400 MHz, $CDCl_3$) δ 7.90 (s, 1H), 6.91 (bs, 1H), 4.34 (t, *J* = 5.2 Hz, 2H), 3.42 (q, *J* = 5.4 Hz, 2H), 2.41 (s, 3H), 2.27 (s, 3H)

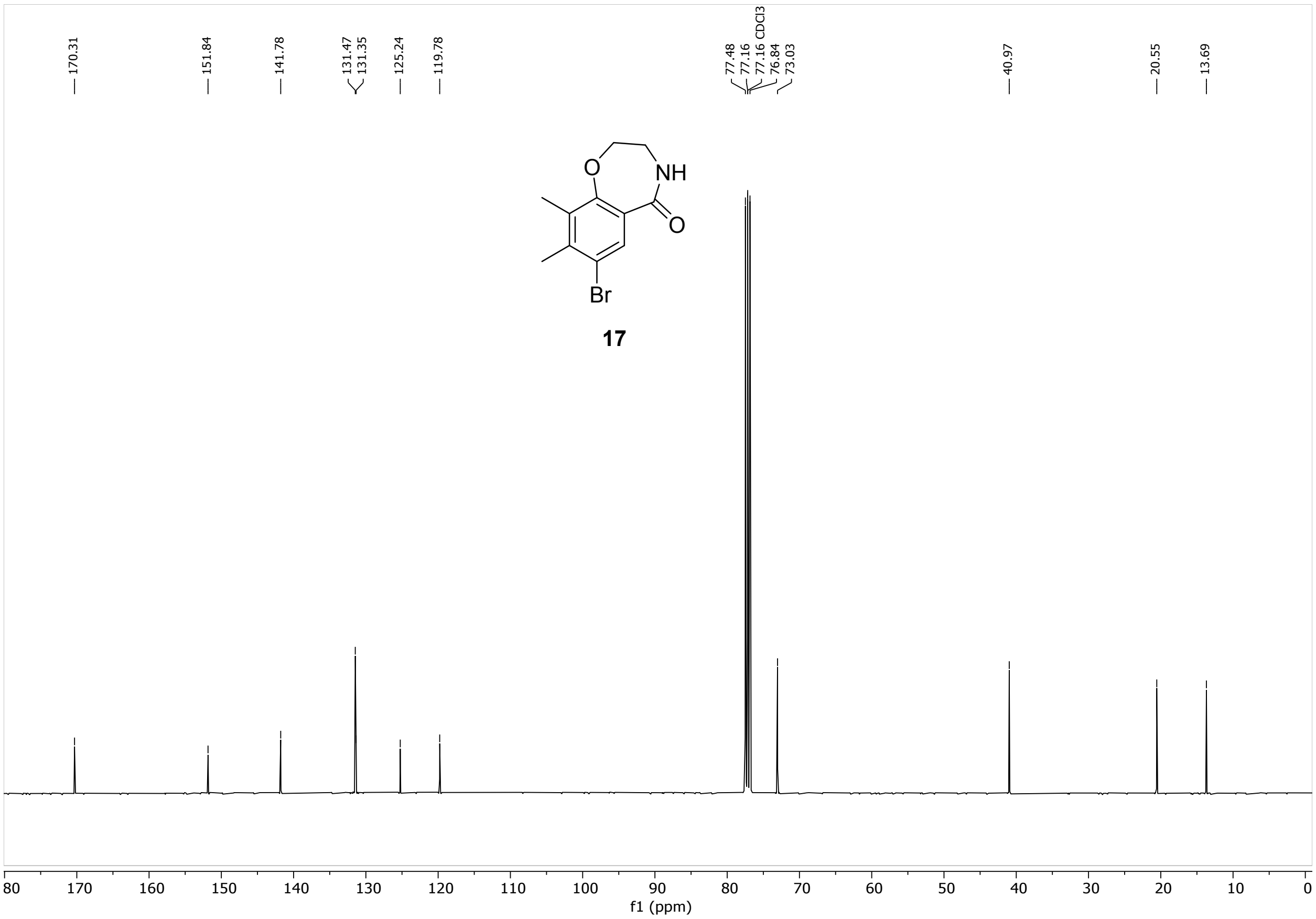


**17**

$^{13}$C NMR (101 MHz, $CDCl_3$) δ 170.3, 151.8, 141.8, 131.5, 131.4, 125.2, 119.8, 73.0, 41.0, 20.6, 13.7.

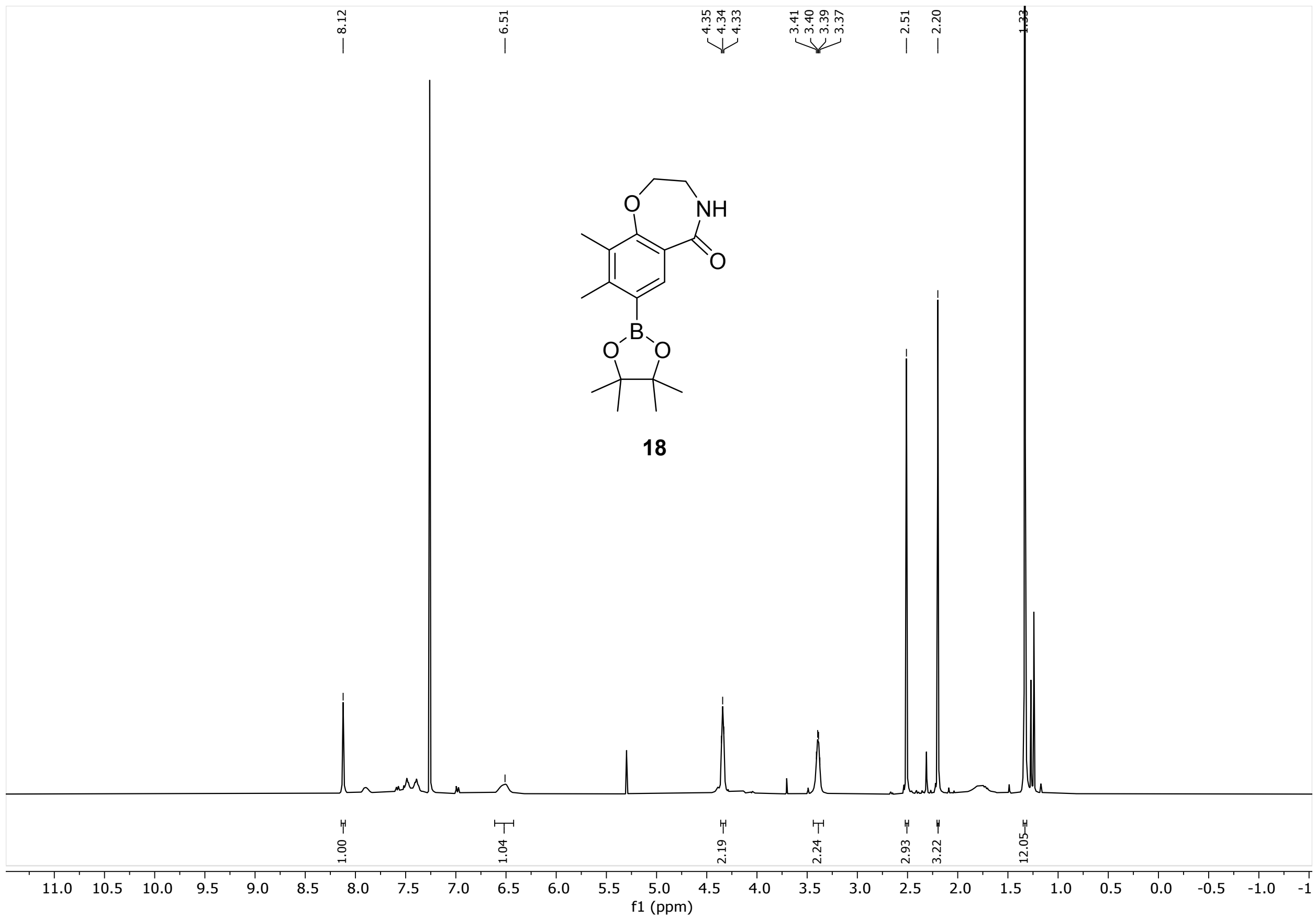


$^1$H NMR (400 MHz, $CDCl_3$) δ 8.12 (s, 1H), 6.51 (m, 1H), 4.34 (t, $J$ = 4.8 Hz, 2H), 3.44 – 3.34 (m, 2H), 2.51 (s, 3H), 2.20 (s, 3H), 1.33 (s, 12H).

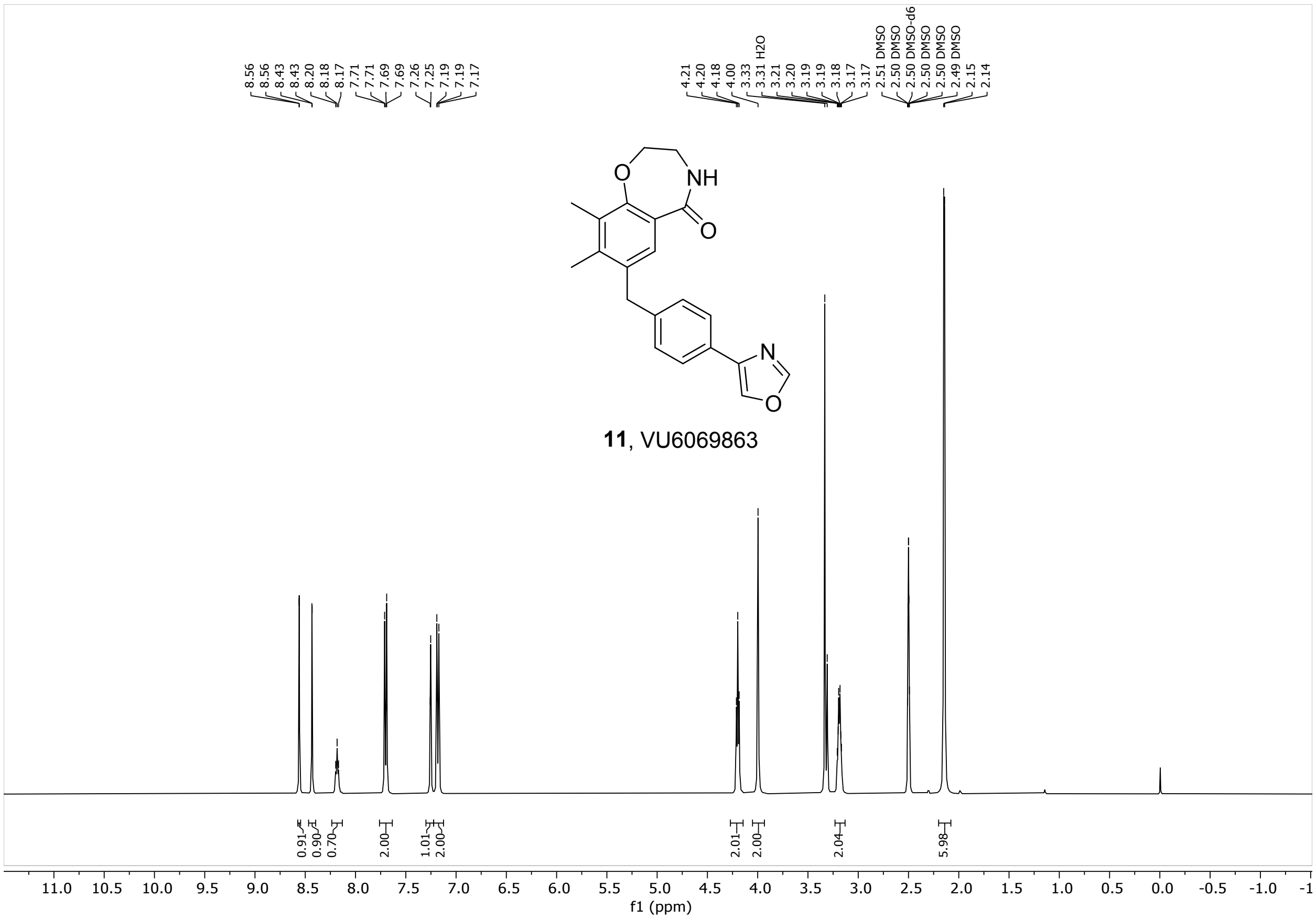


$^{1}$H NMR (400 MHz, DMSO-$d_6$) δ 8.56 (d, $J$ = 1.0 Hz, 1H), 8.43 (d, $J$ = 1.0 Hz, 1H), 8.19 (t, $J$ = 5.5 Hz, 1H), 7.73 – 7.67 (m, 2H), 7.26 (s, 1H), 7.19 – 7.17 (m, 2H), 4.20 (t, $J$ = 5.3 Hz, 2H), 3.99 (s, 2H), 3.34 (s, 1H), 3.19 (q, $J$ = 5.4 Hz, 2H), 2.14 (s, 3H), 2.15 (s, 3H).

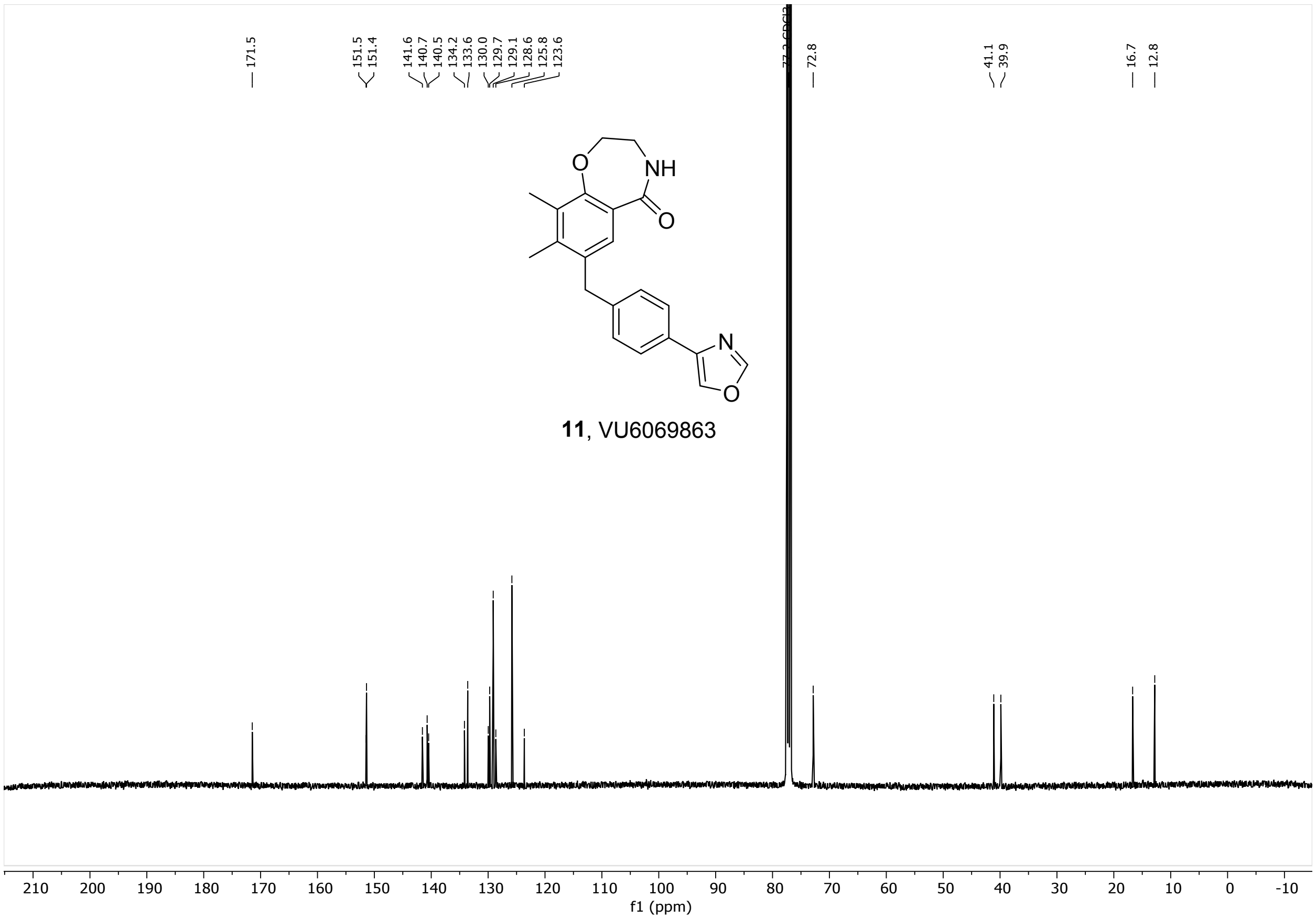


$^{13}C$ NMR (101 MHz, $CDCl_3$) δ 171.5, 151.5, 151.4, 141.6, 140.7, 140.5, 134.2, 133.6, 130.0, 129.7, 129.1 (2), 128.6, 125.8 (2), 123.6, 72.8, 41.1, 39.9, 16.7, 12.8.

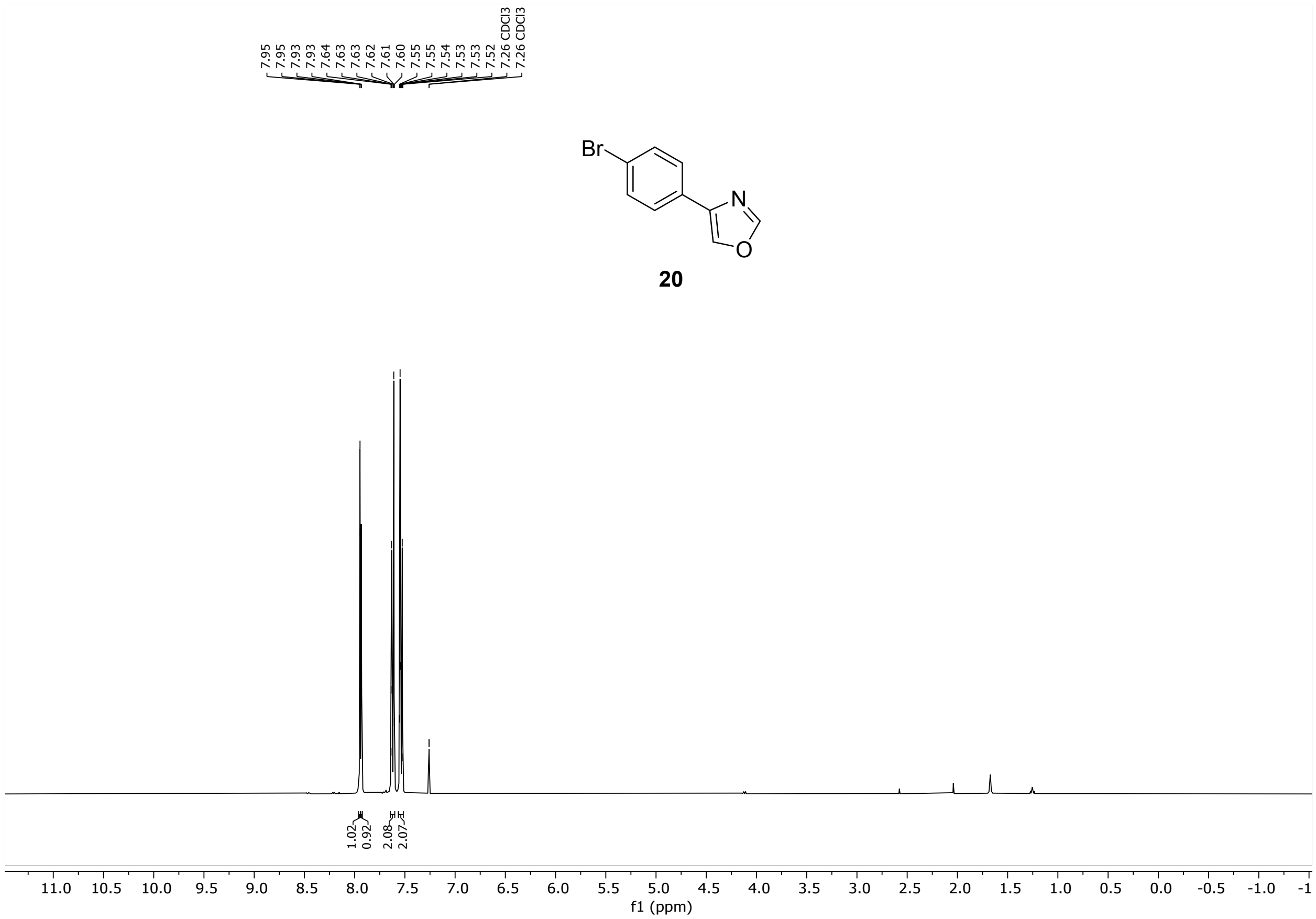

$^1H$ NMR (400 MHz, $CDCl_3$) δ 7.95 (d, *J* = 0.9 Hz, 1H), 7.94 (s, 1H), 7.65 – 7.59 (m, 2H), 7.57 – 7.51 (m, 2H).

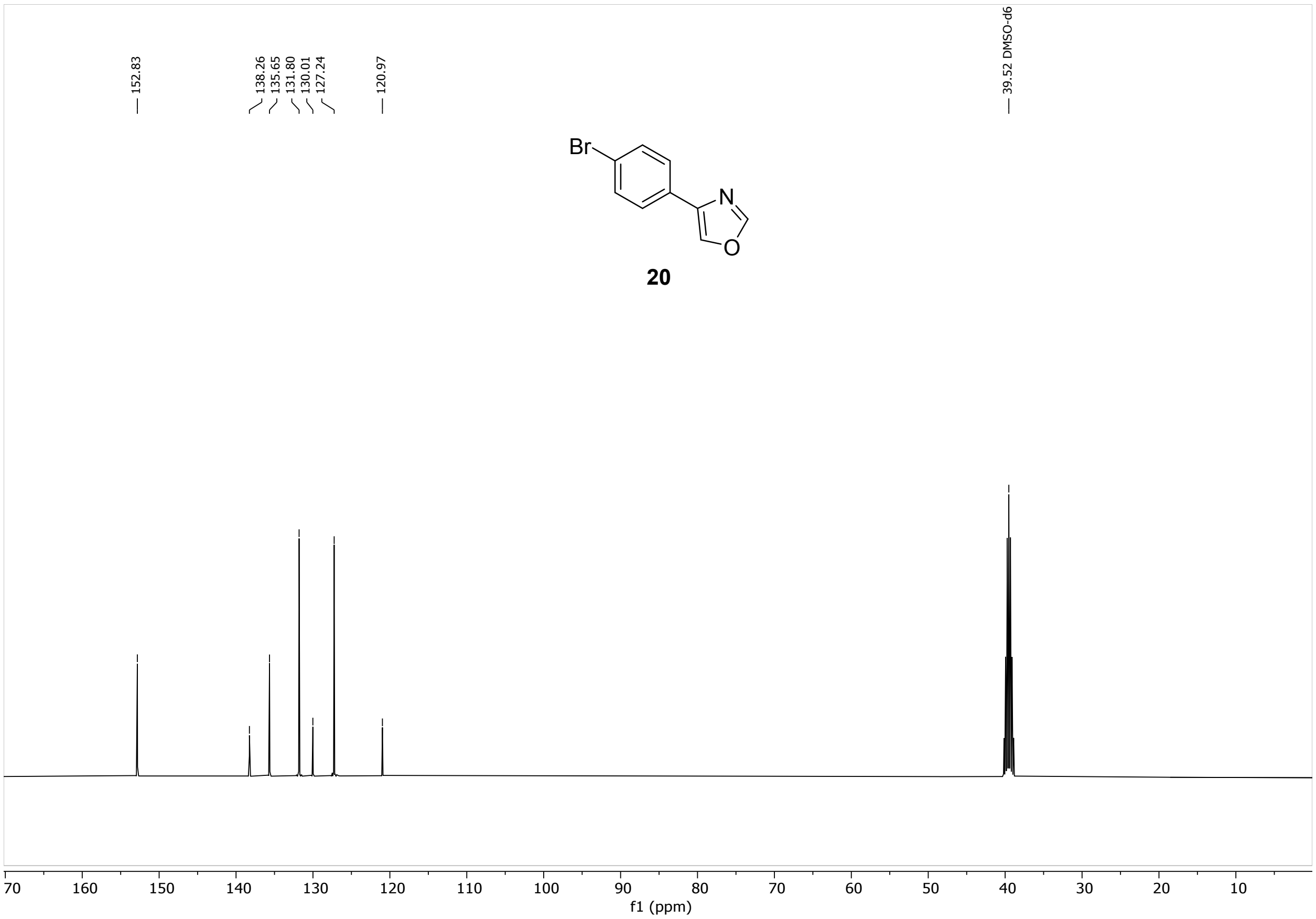


$^{13}$C NMR (101 MHz, DMSO-$d_6$) δ 152.8, 138.3, 135.7, 131.8 (2C), 130.0, 127.2 (2C), 121.0

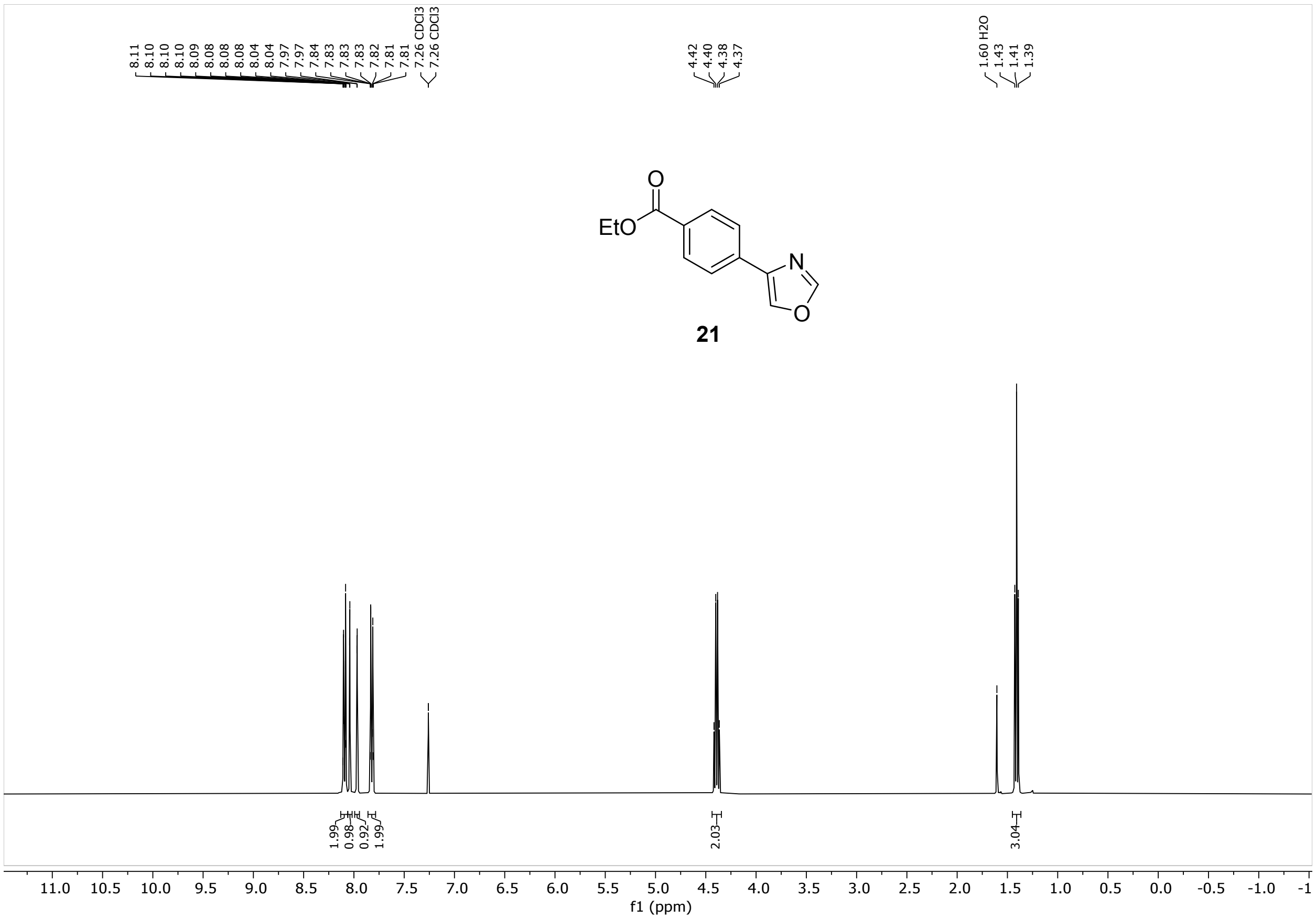

$^{1}H$ NMR (400 MHz, $CDCl_3$) δ 8.11 – 8.06 (m, 2H), 8.04 (d, *J* = 0.9 Hz, 1H), 7.97 (d, *J* = 0.9 Hz, 1H), 7.86 – 7.79 (m, 2H), 4.39 (q, *J* = 7.1 Hz, 2H), 1.41 (t, *J* = 7.1 Hz, 3H).

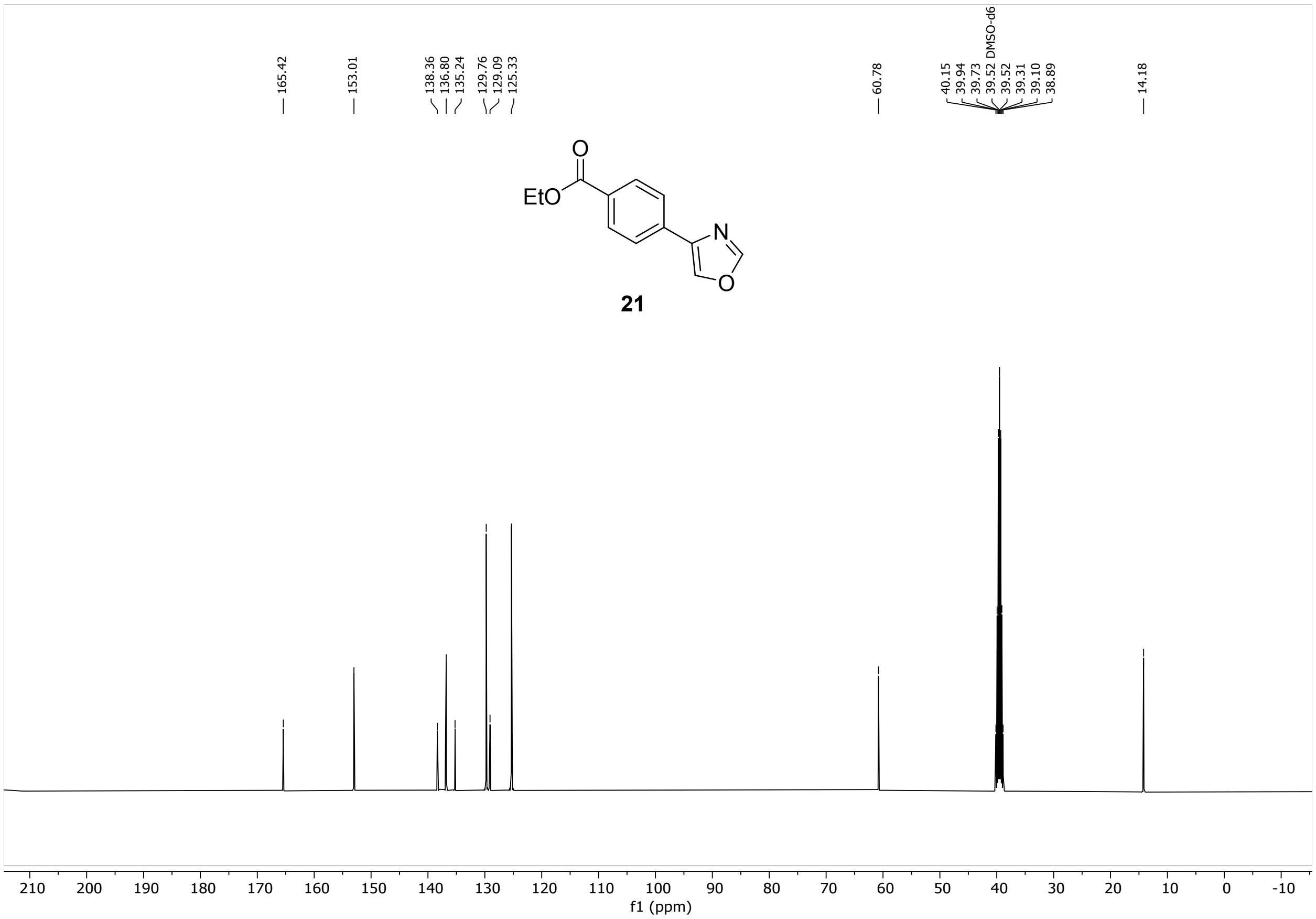


$^{13}$C NMR (101 MHz, DMSO-$d_6$) δ 165.4, 153.0, 138.4, 136.8, 135.2, 129.8 (2C), 129.1, 125.3 (2C), 60.8, 34.2.

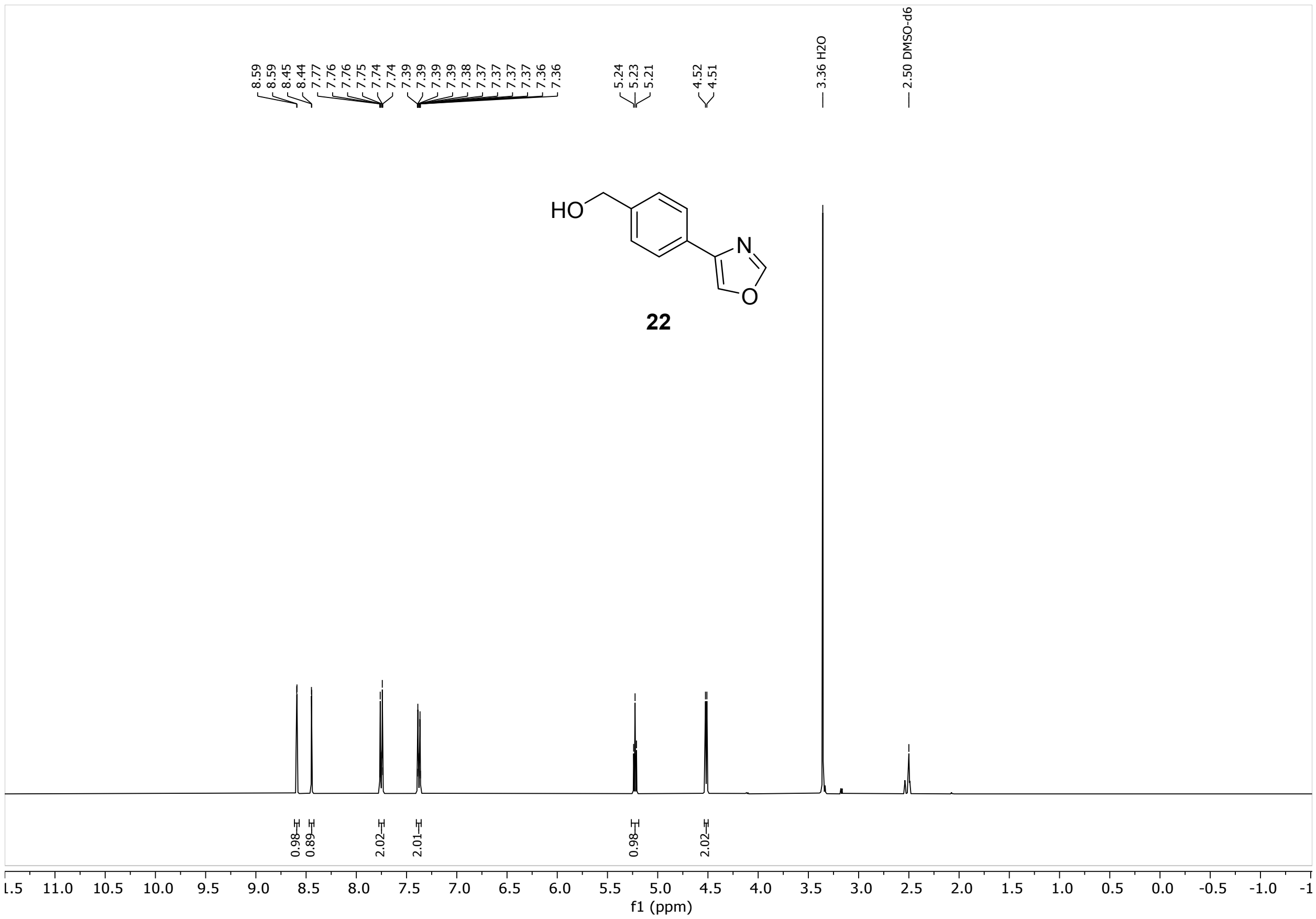


$^1$H NMR (400 MHz, DMSO-$d_6$) δ 8.59 (d, $J$ = 0.9 Hz, 1H), 8.45 (d, $J$ = 1.0 Hz, 1H), 7.78 – 7.72 (m, 2H), 7.39 – 7.36 (m, 2H), 5.23 (t, $J$ = 5.7 Hz, 1H), 4.52 (d, $J$ = 5.7 Hz, 2H).

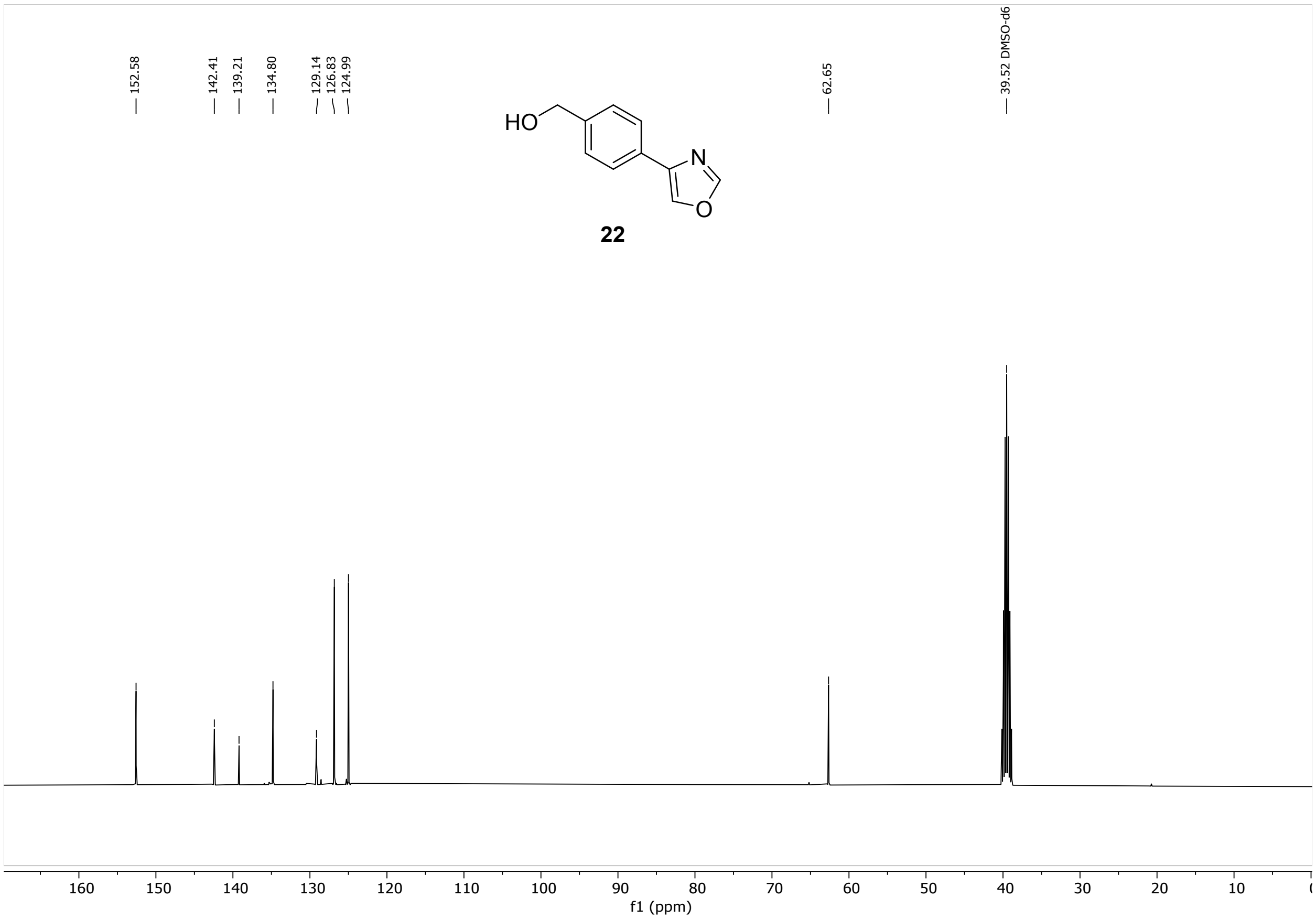


$^{13}C$ NMR (101 MHz, DMSO-$d_6$) δ 152.6, 142.4, 139.2, 134.8, 129.1, 126.8 (2C), 125.0 (2C), 62.65.

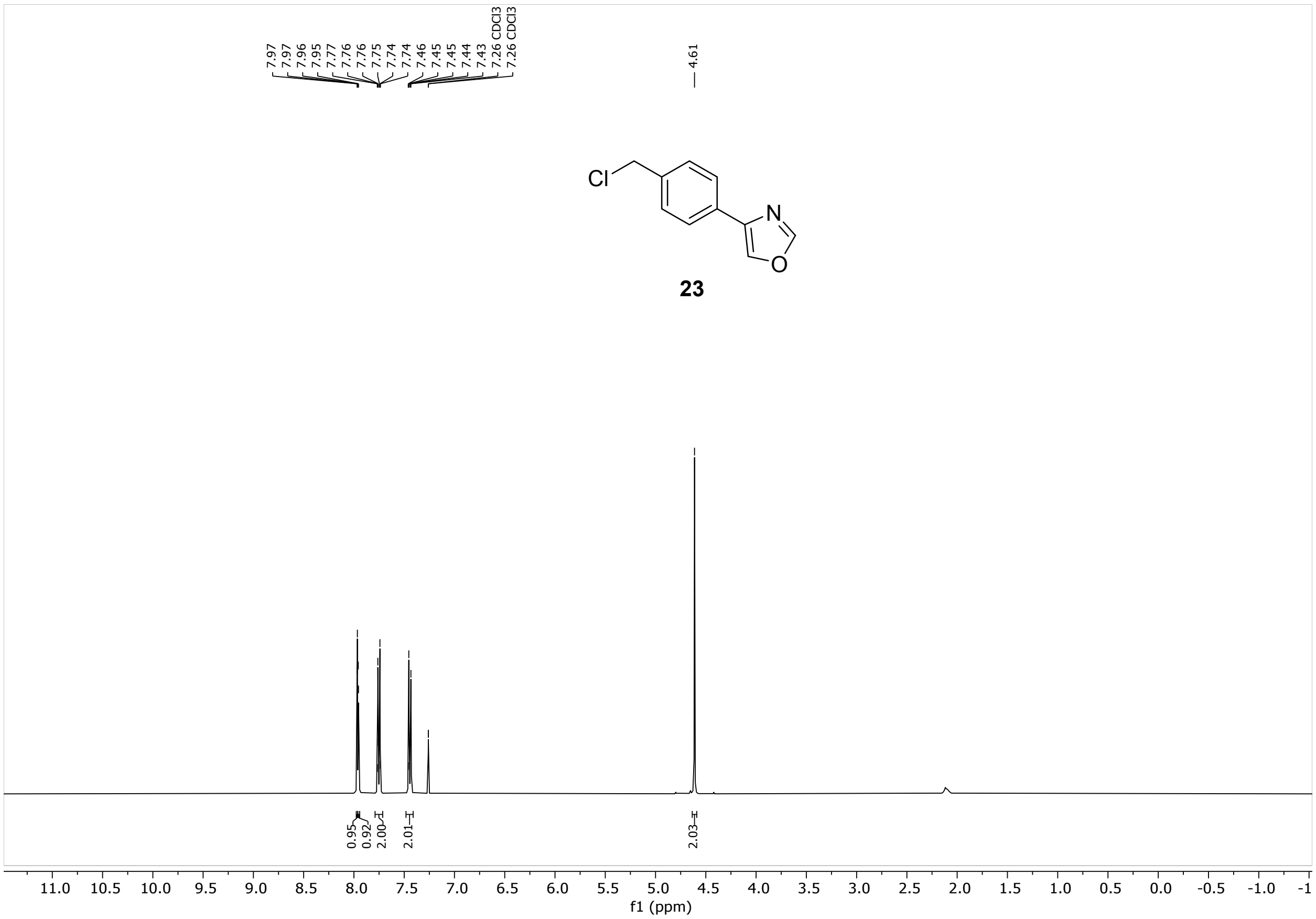

$^{1}$H NMR (400 MHz, $CDCl_3$) δ 7.97 (d, *J* = 0.9 Hz, 1H), 7.96 (d, *J* = 1.0 Hz, 1H), 7.79 – 7.71 (m, 2H), 7.48 – 7.40 (m, 2H), 4.61 (s, 2H).

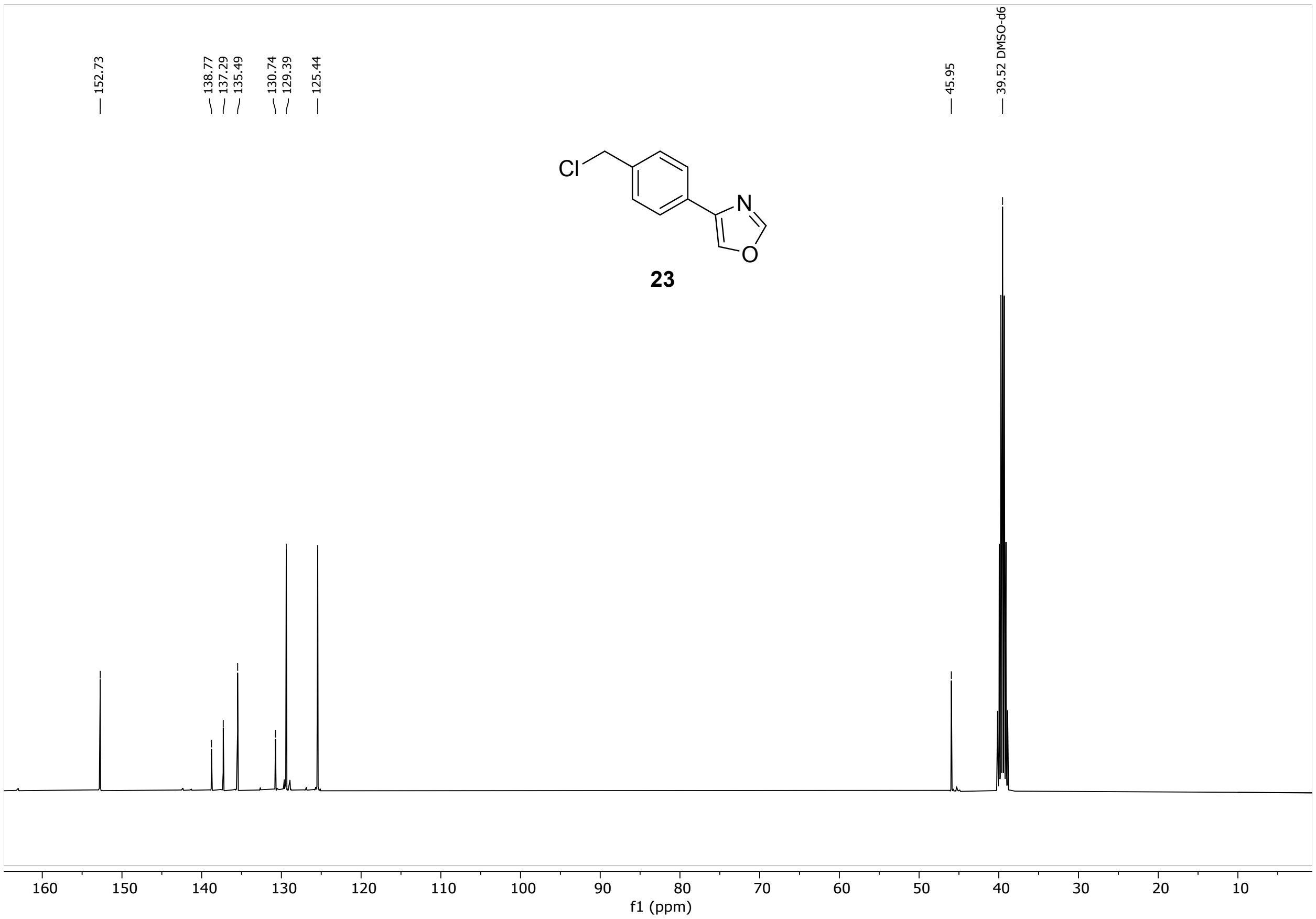


$^{13}C$ NMR (101 MHz, DMSO-$d_6$) δ 152.7, 138.8, 137.3, 135.5, 130.7, 129.4 (2C), 125.4 (2C), 46.0.

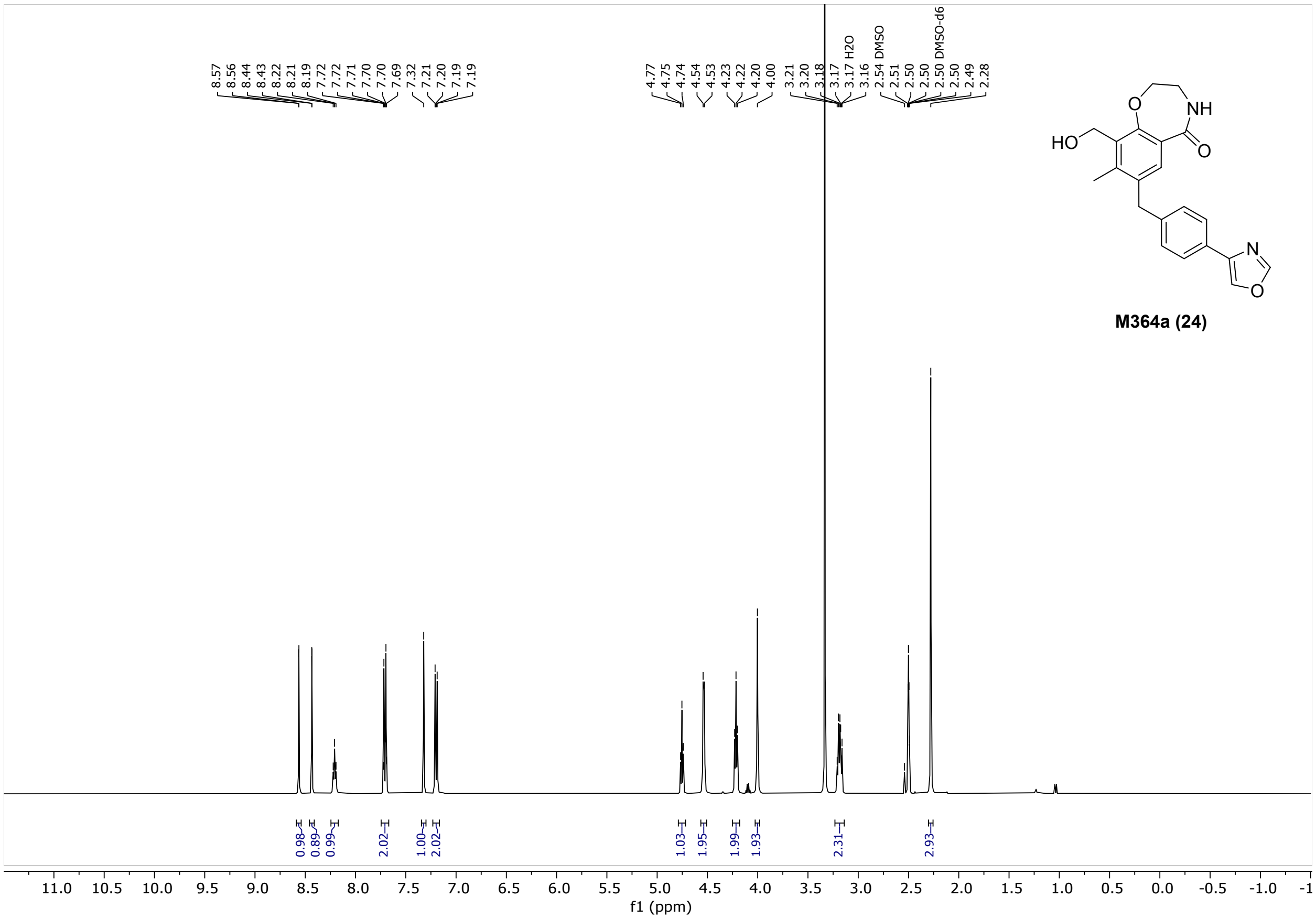


$^{1}$H NMR (400 MHz, DMSO-$d_6$) δ 8.56 (d, $J$ = 1.0 Hz, 1H), 8.43 (d, $J$ = 1.0 Hz, 1H), 8.21 (t, $J$ = 5.5 Hz, 1H), 7.75 – 7.67 (m, 2H), 7.32 (s, 1H), 7.23 – 7.17 (m, 2H), 4.75 (t, $J$ = 5.2 Hz, 1H), 4.54 (d, $J$ = 5.3 Hz, 2H), 4.22 (t, $J$ = 5.4 Hz, 2H), 4.00 (s, 2H), 3.23 – 3.14 (m, 2H), 2.28 (s, 3H).

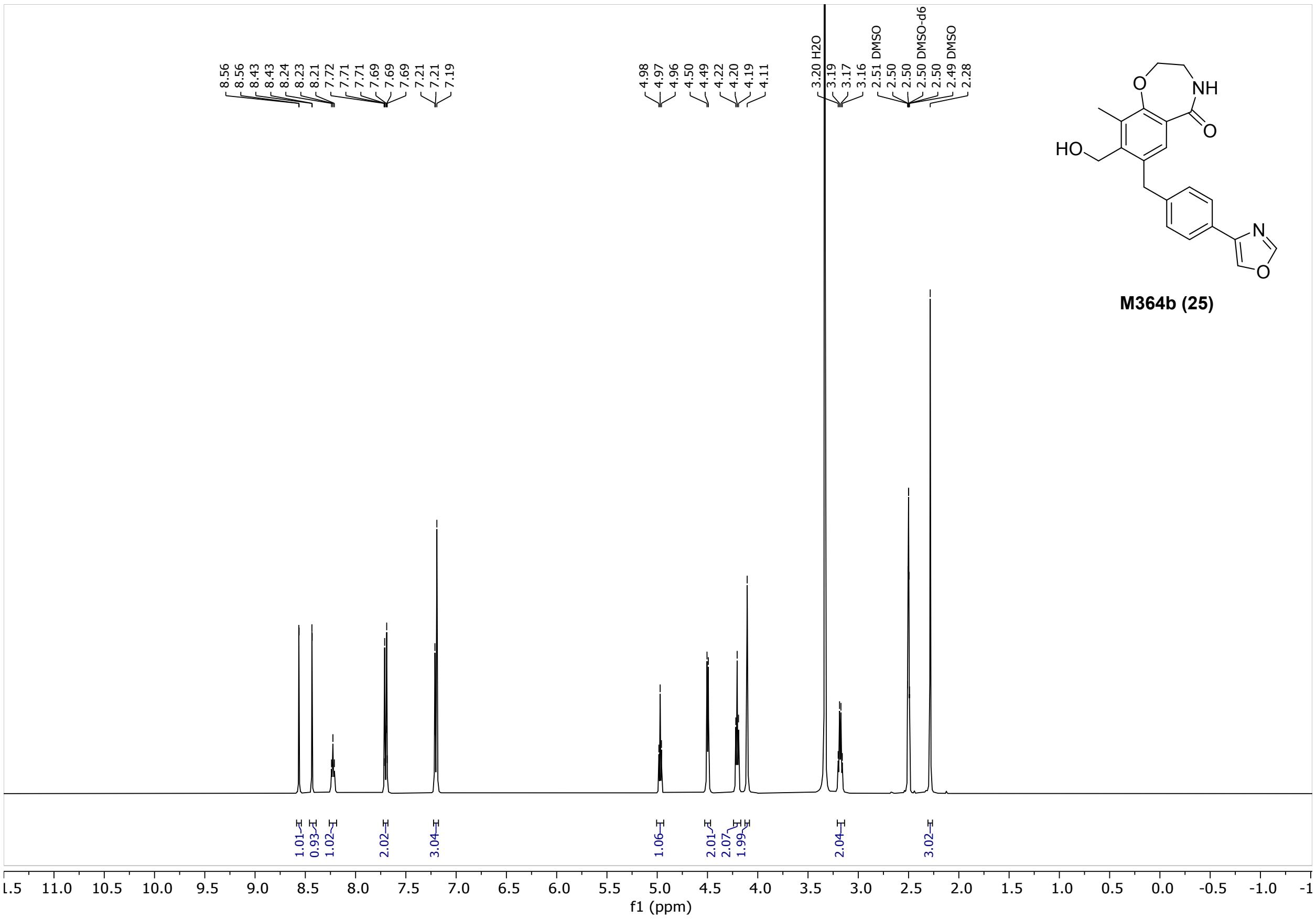


$^{1}$H NMR (400 MHz, DMSO-$d_6$) δ 8.56 (d, $J$ = 1.0 Hz, 1H), 8.43 (d, $J$ = 1.0 Hz, 1H), 8.23 (t, $J$ = 5.6 Hz, 1H), 7.74 – 7.66 (m, 2H), 7.21 – 7.19 (m, 3H), 4.97 (t, $J$ = 5.1 Hz, 1H), 4.50 (d, $J$ = 5.1 Hz, 2H), 4.20 (t, $J$ = 5.3 Hz, 2H), 4.11 (s, 2H), 3.21 – 3.14 (m, 2H), 2.28 (s, 3H).

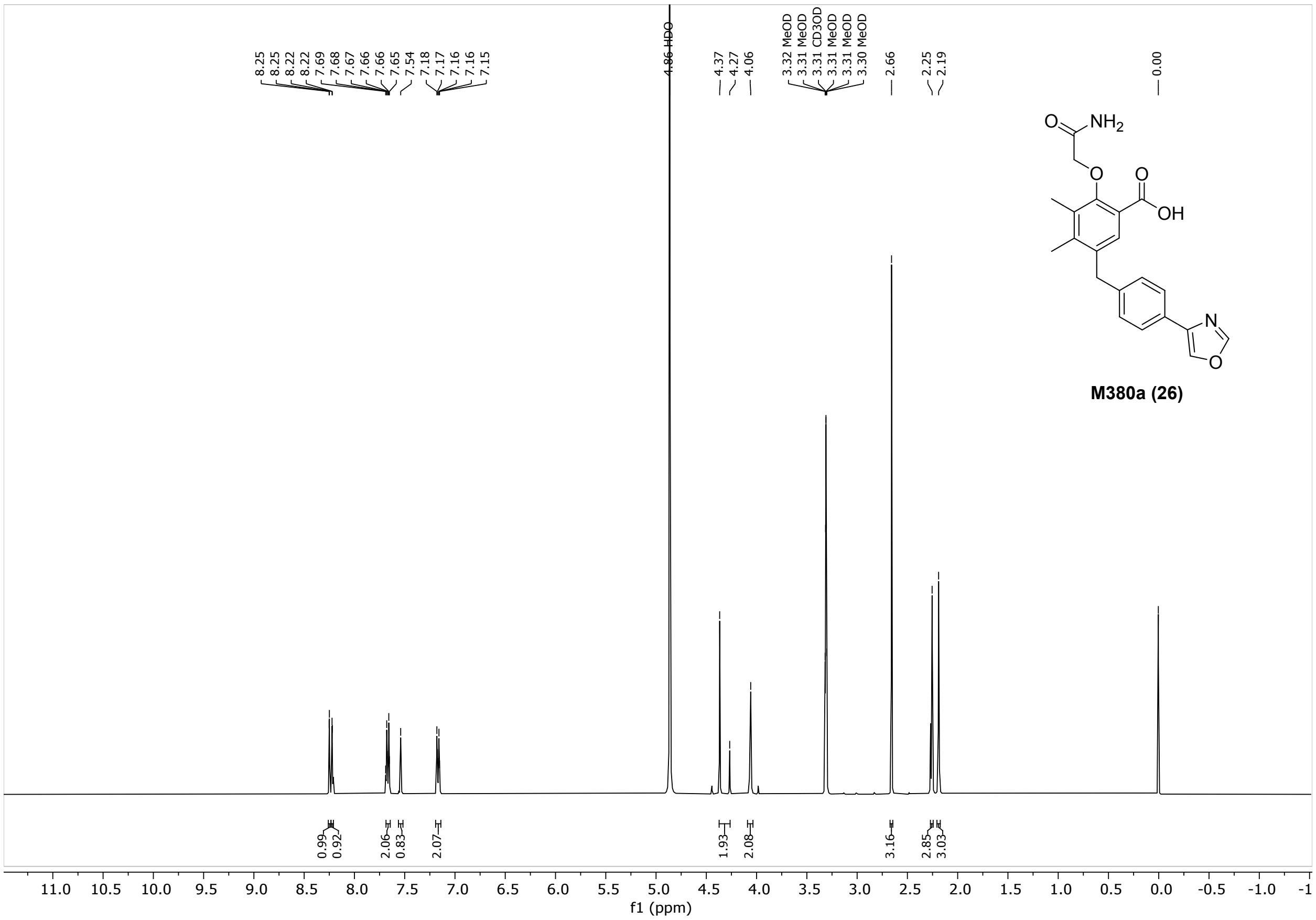

M380a (26)

$^{1}$H NMR (400 MHz, MeOD) δ 8.25 (d, $J$ = 1.0 Hz, 1H), 8.22 (d, $J$ = 1.0 Hz, 1H), 7.68 – 7.65 (m, 2H), 7.54 (s, 1H), 7.18 – 7.15 (m, 2H), 4.32 (s, 2H), 4.06 (s, 2H), 2.66 (s, 3H), 2.25 (s, 3H), 2.19 (s, 3H)

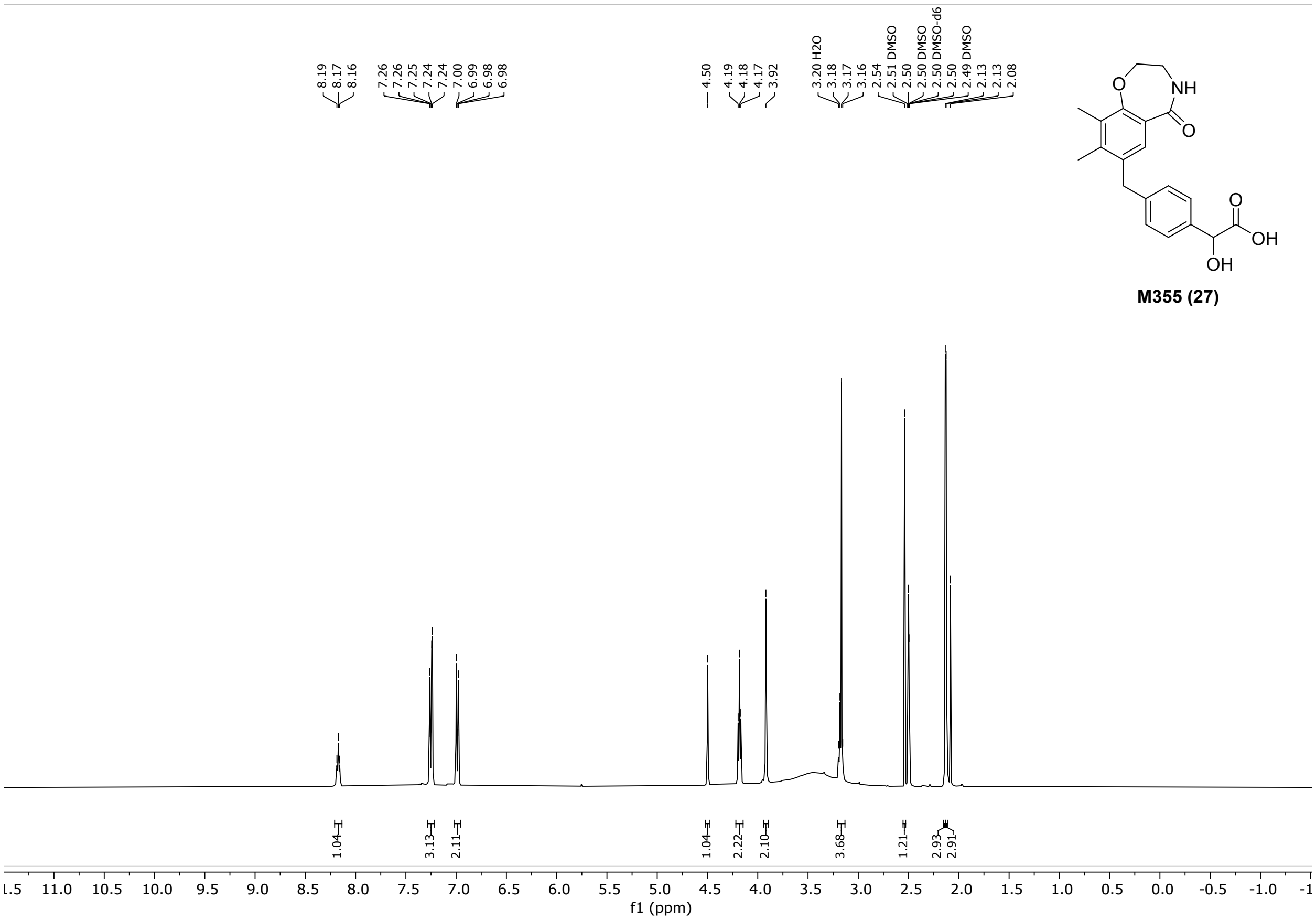

M355 (27)

$^{1}$H NMR (400 MHz, DMSO-$d_6$) δ 8.17 (t, $J$ = 5.6 Hz, 1H), 7.29 – 7.21 (m, 3H), 7.02 – 6.96 (m, 2H), 4.50 (s, 1H), 4.18 (t, $J$ = 5.4 Hz, 2H), 3.92 (s, 2H), 3.20 – 3.16 (m, 2H), 2.54 (s, 1H), 2.13 (s, 3H), 2.13 (s, 3H), COOH proton not observable.

# Purity Analysis by LC-MS

| Structure | Compound # | LC traces (215 and 265 nm) |
|---|---|---|
| 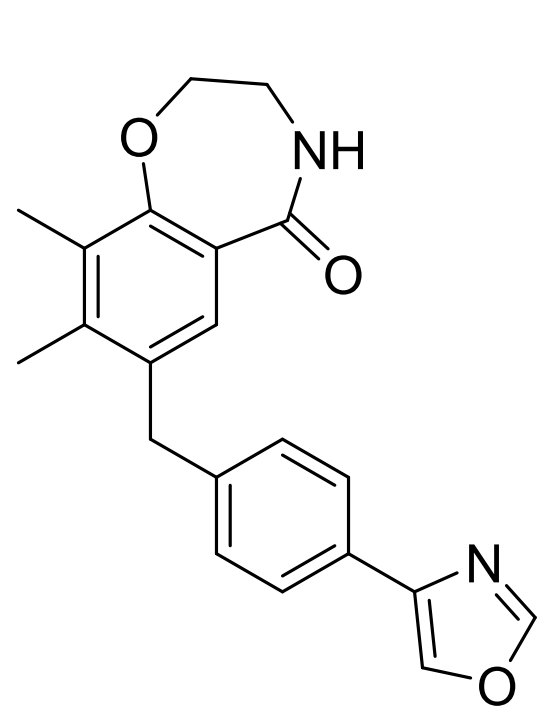 | **11,**<br>VU6069863 | 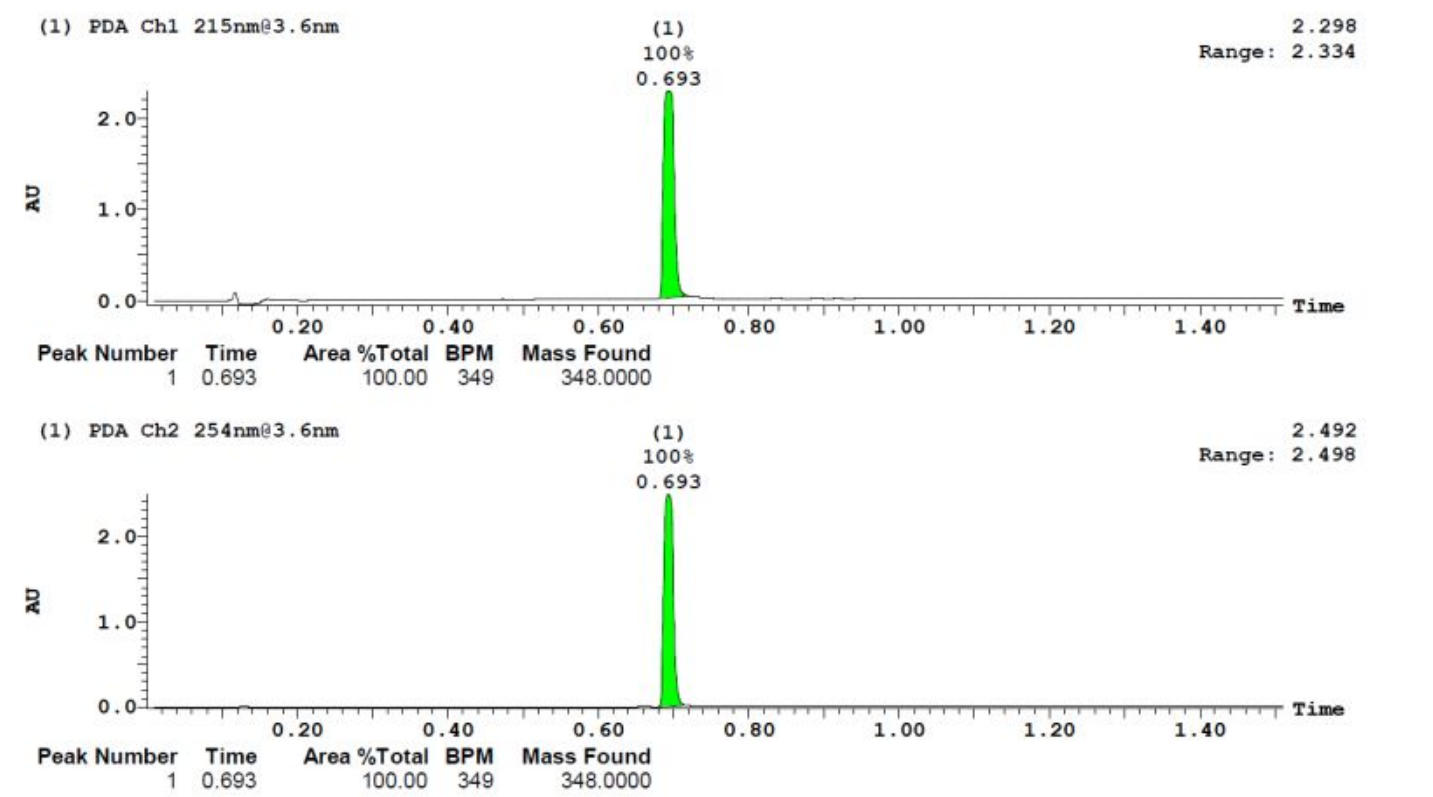 |
| 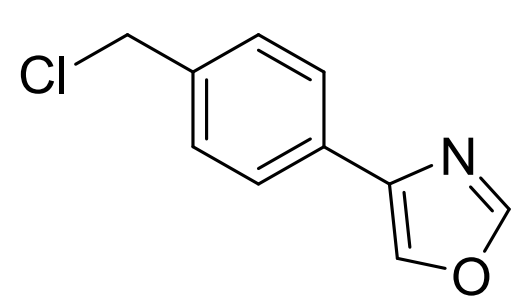 | **23** | 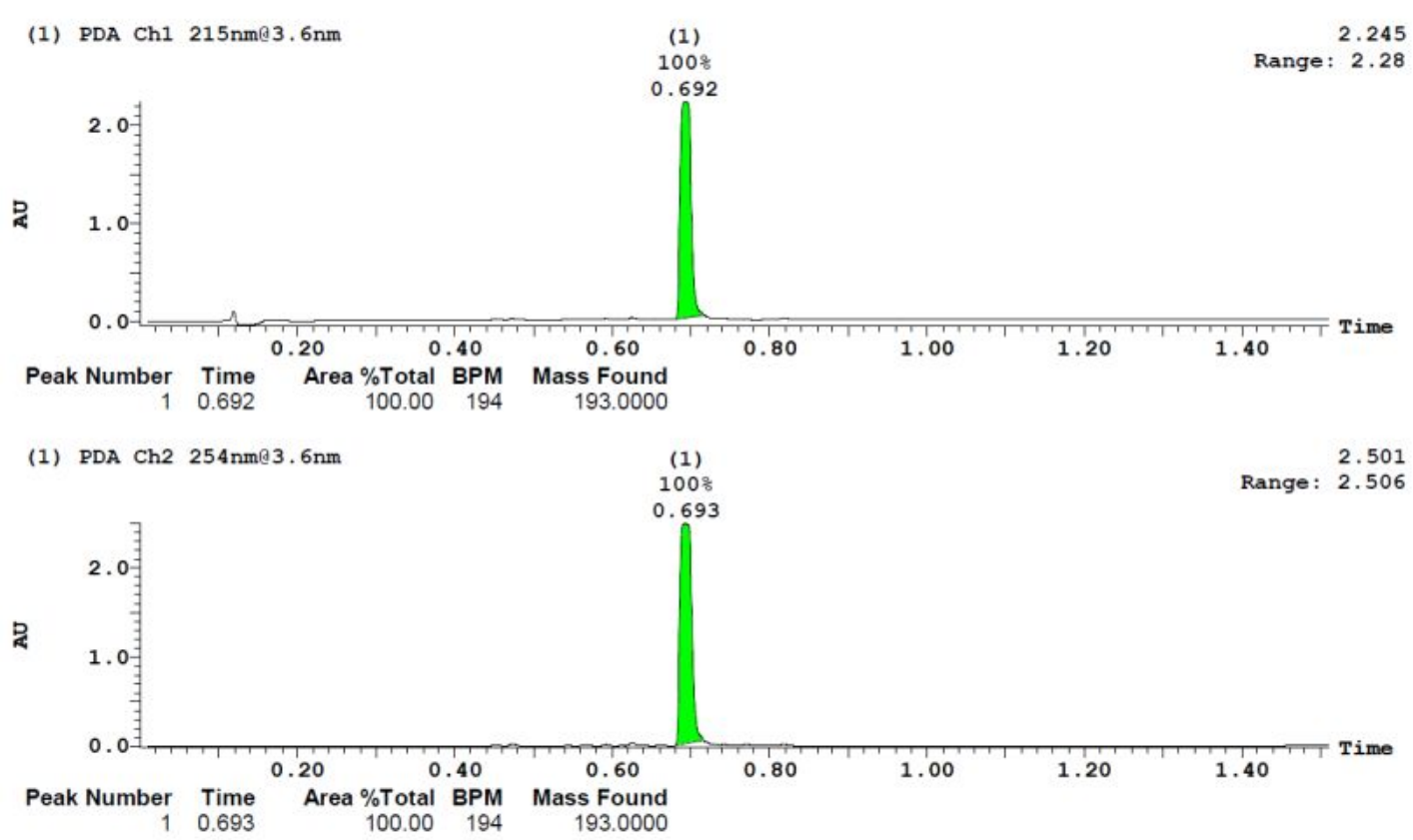 |
| 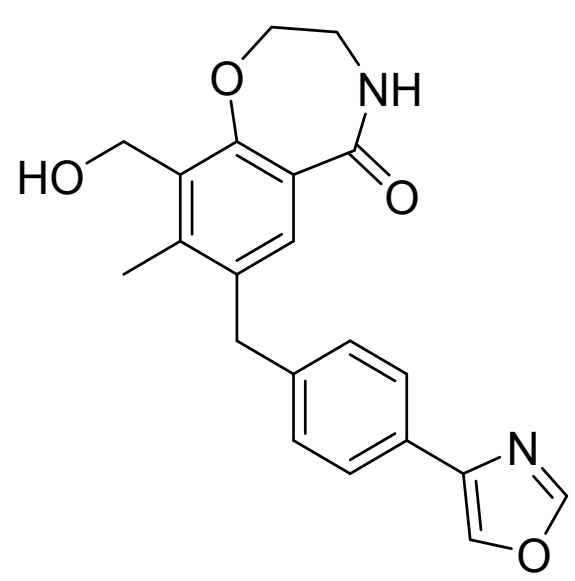 | **24 (M364a)** | 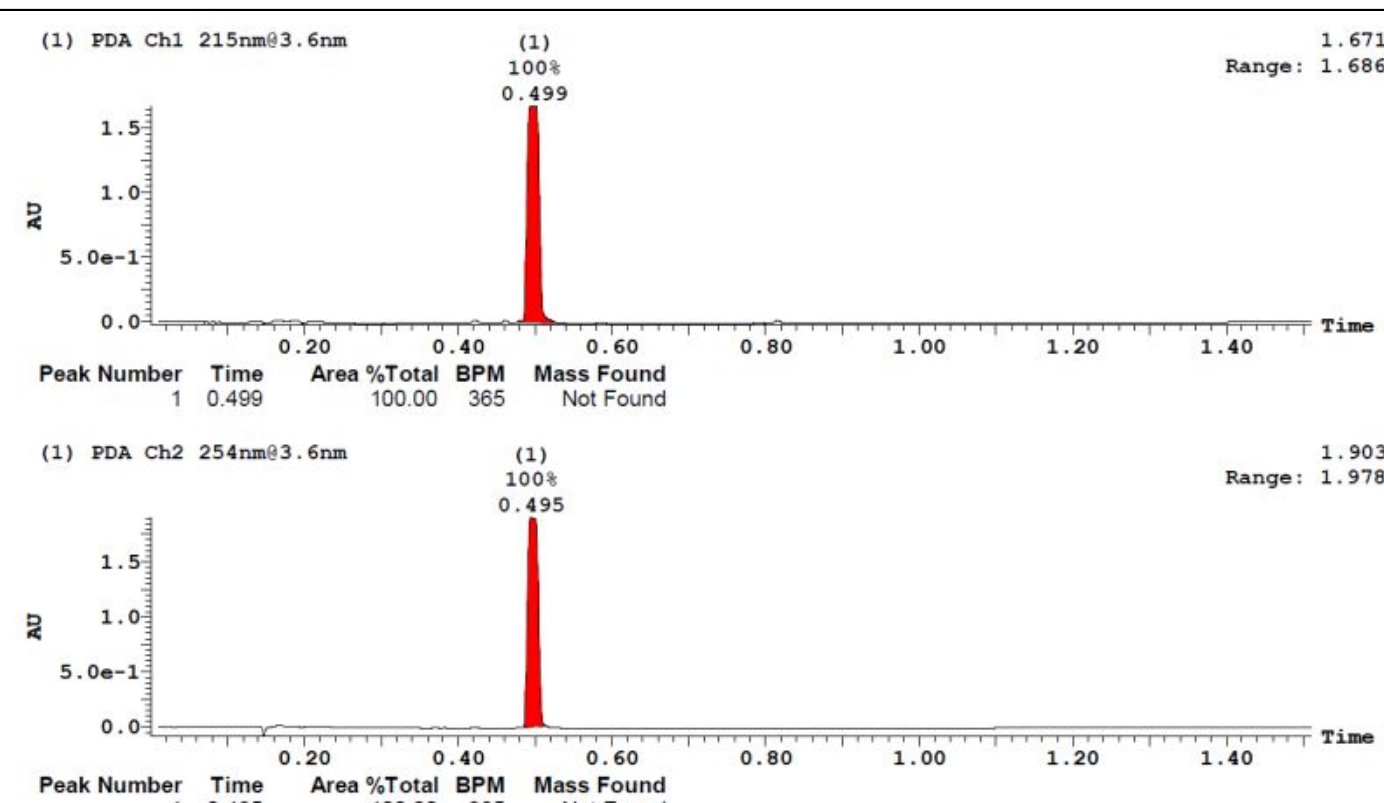 |

<table>
<tr>
<td>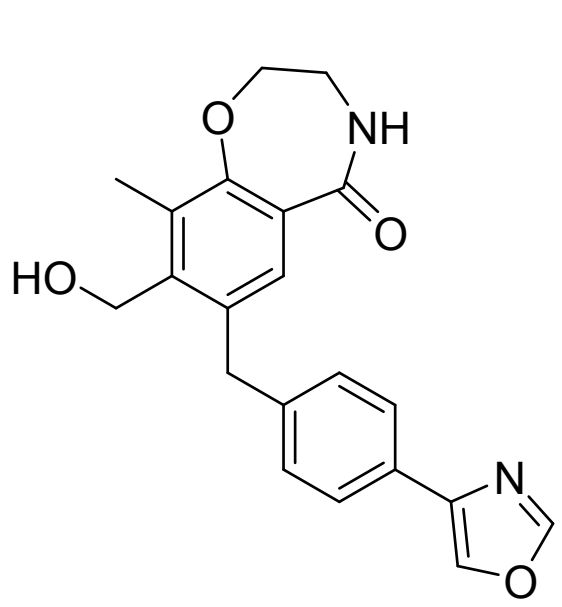</td>
<td>25 (M364b)</td>
<td>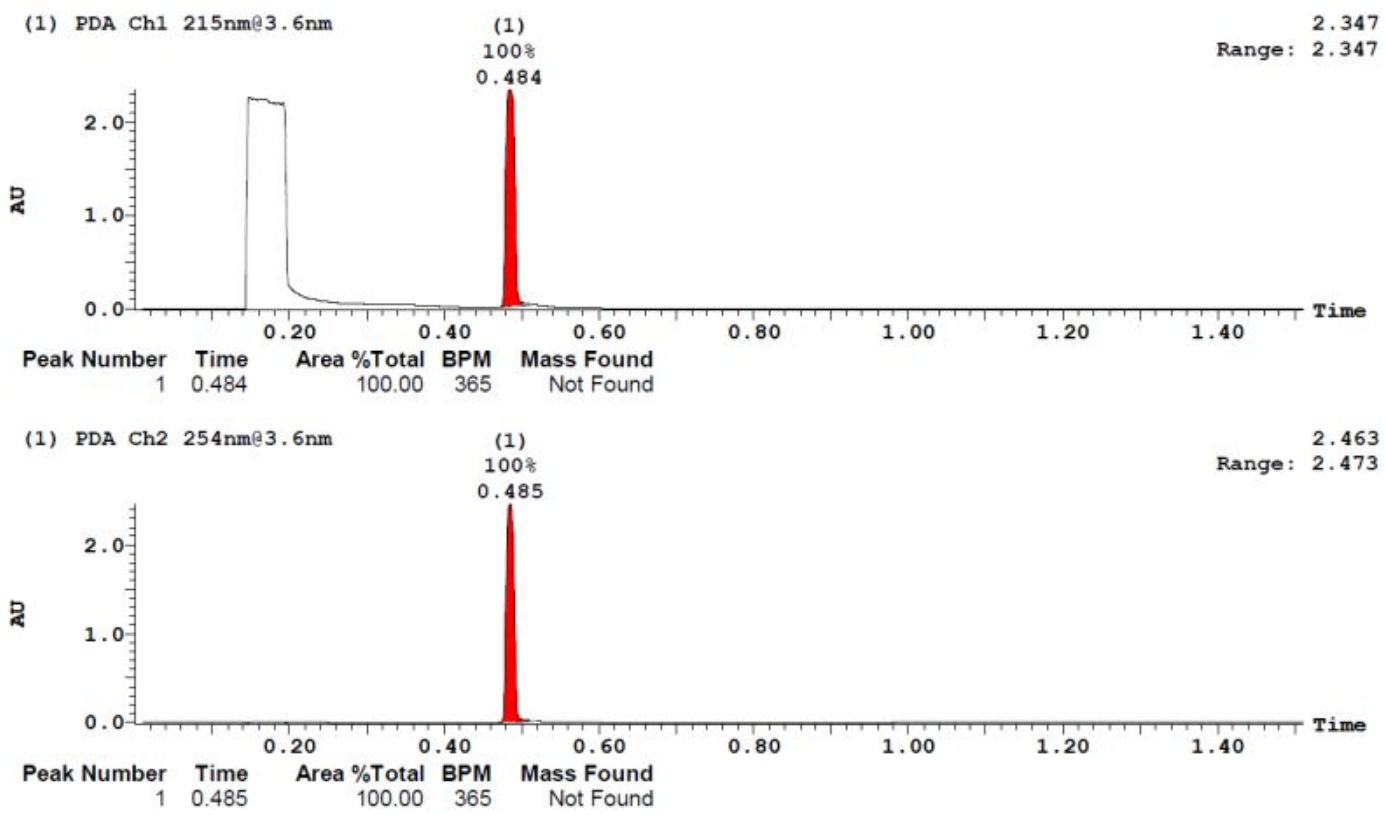
</td>
</tr>
<tr>
<td>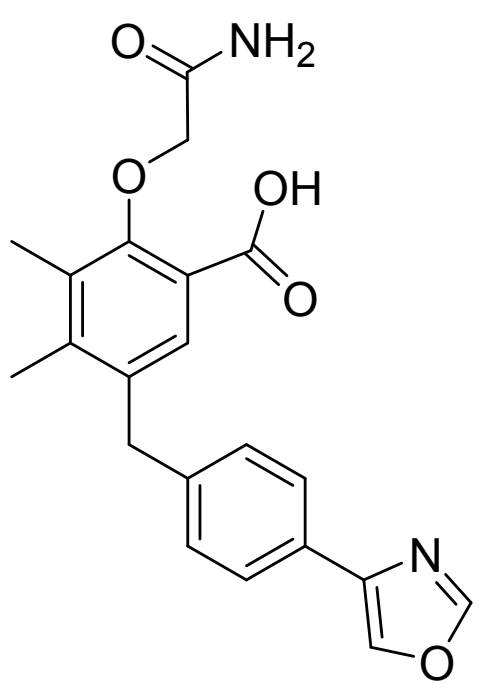</td>
<td>26 (M380a)</td>
<td>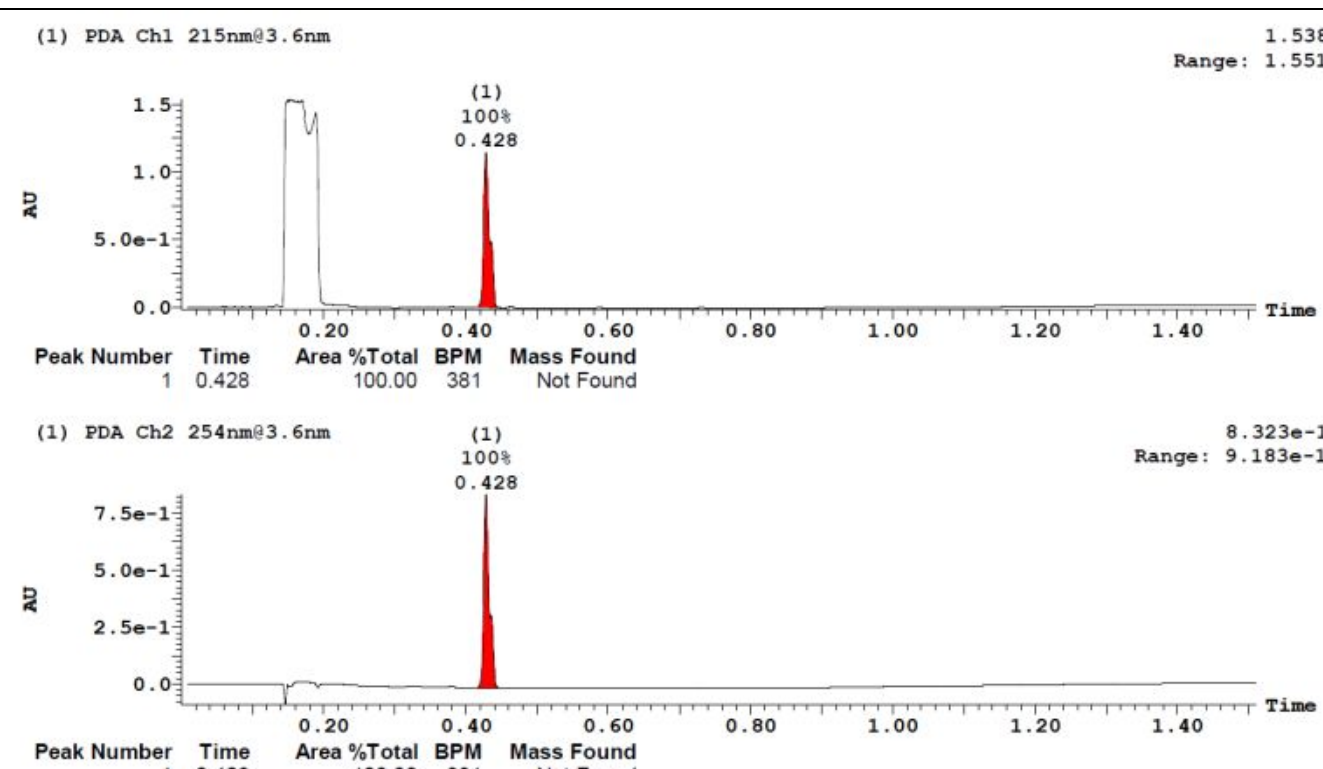
</td>
</tr>
<tr>
<td>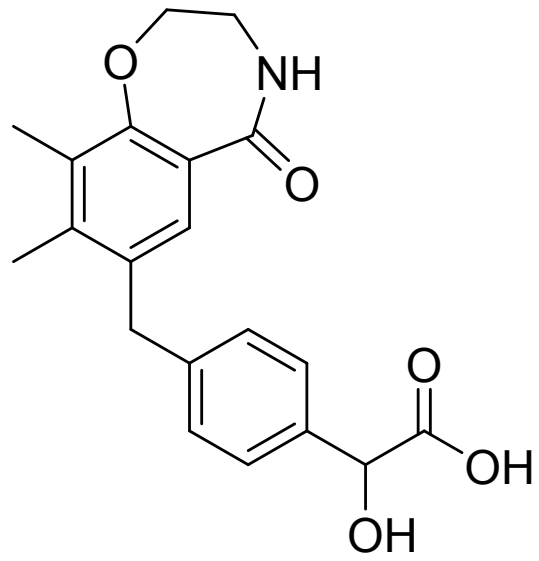</td>
<td>27 (M355)</td>
<td>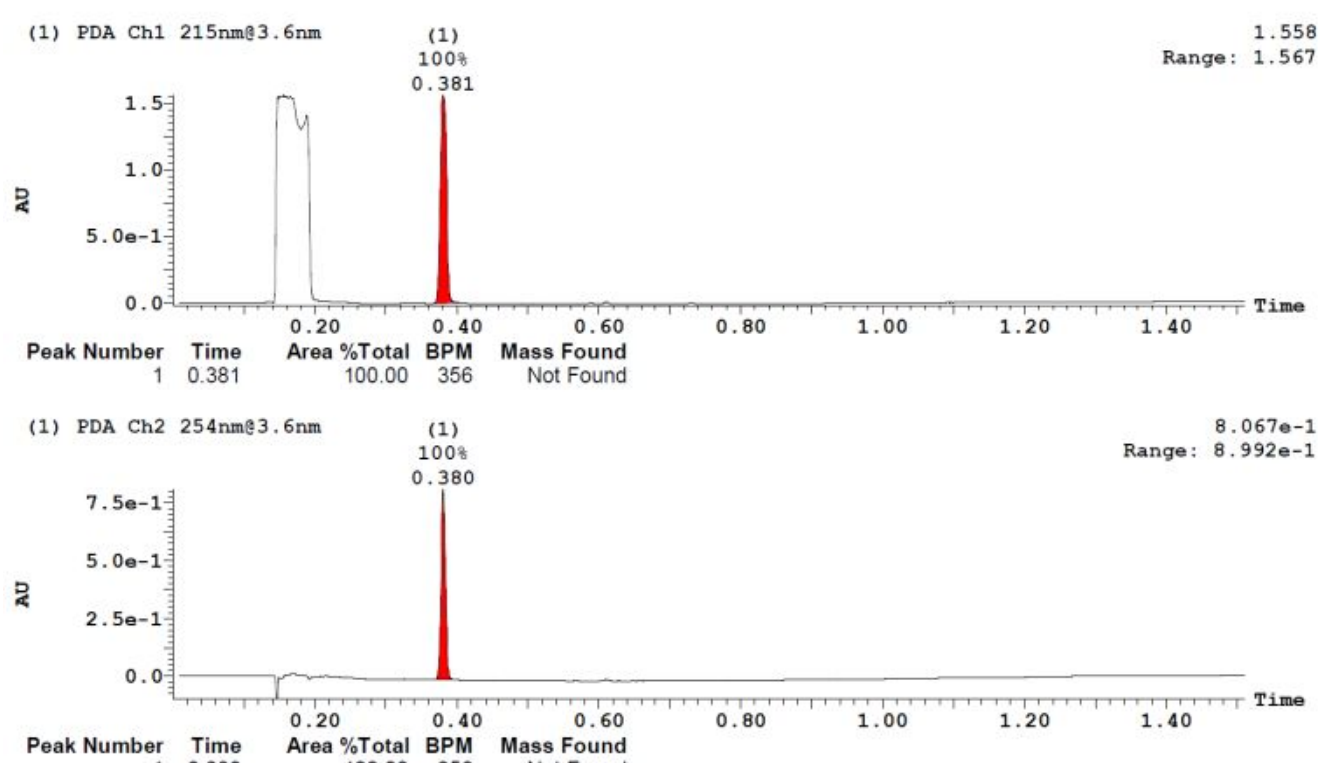
</td>
</tr>
</table>

# X-Ray Structure of VU6069863

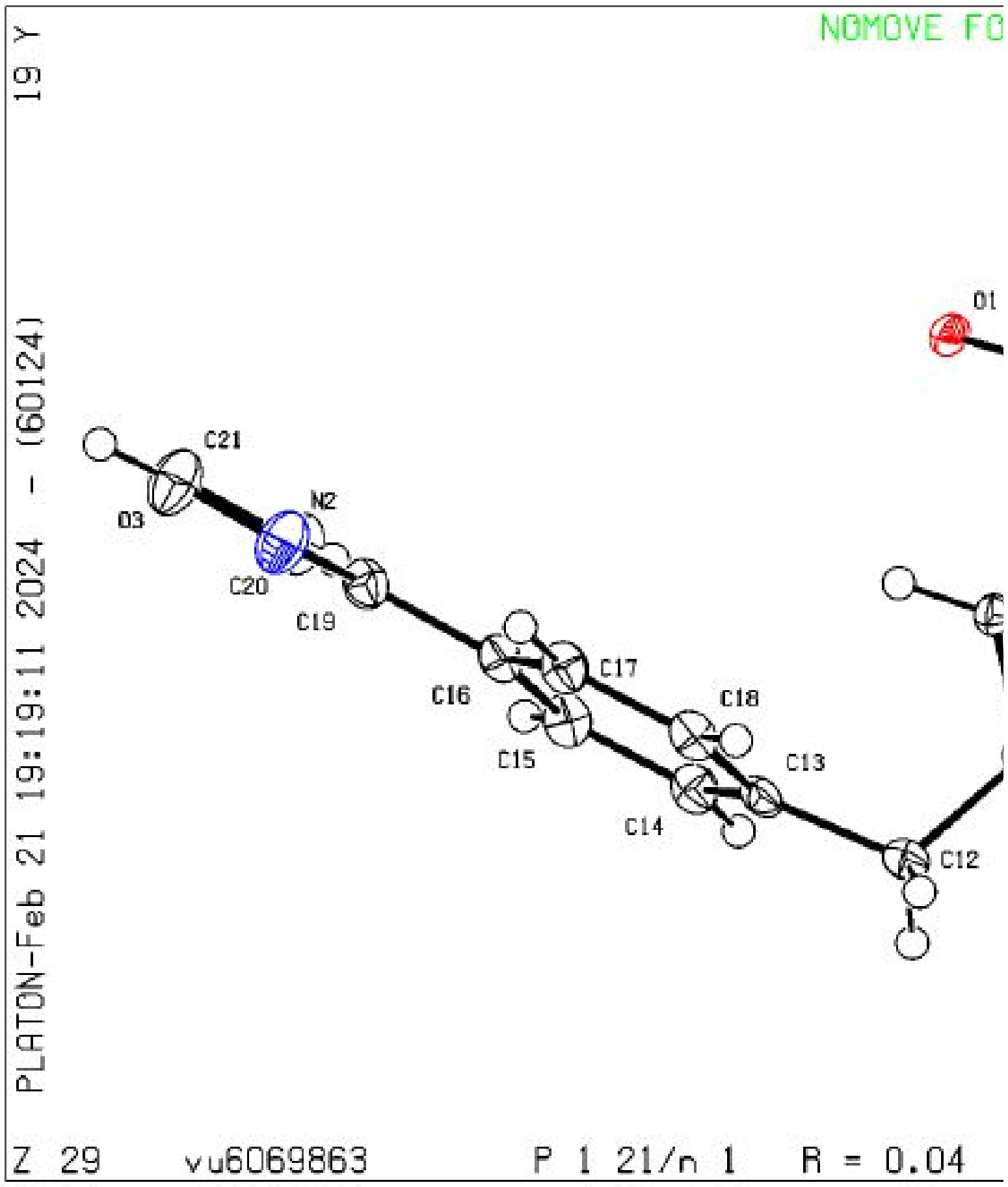


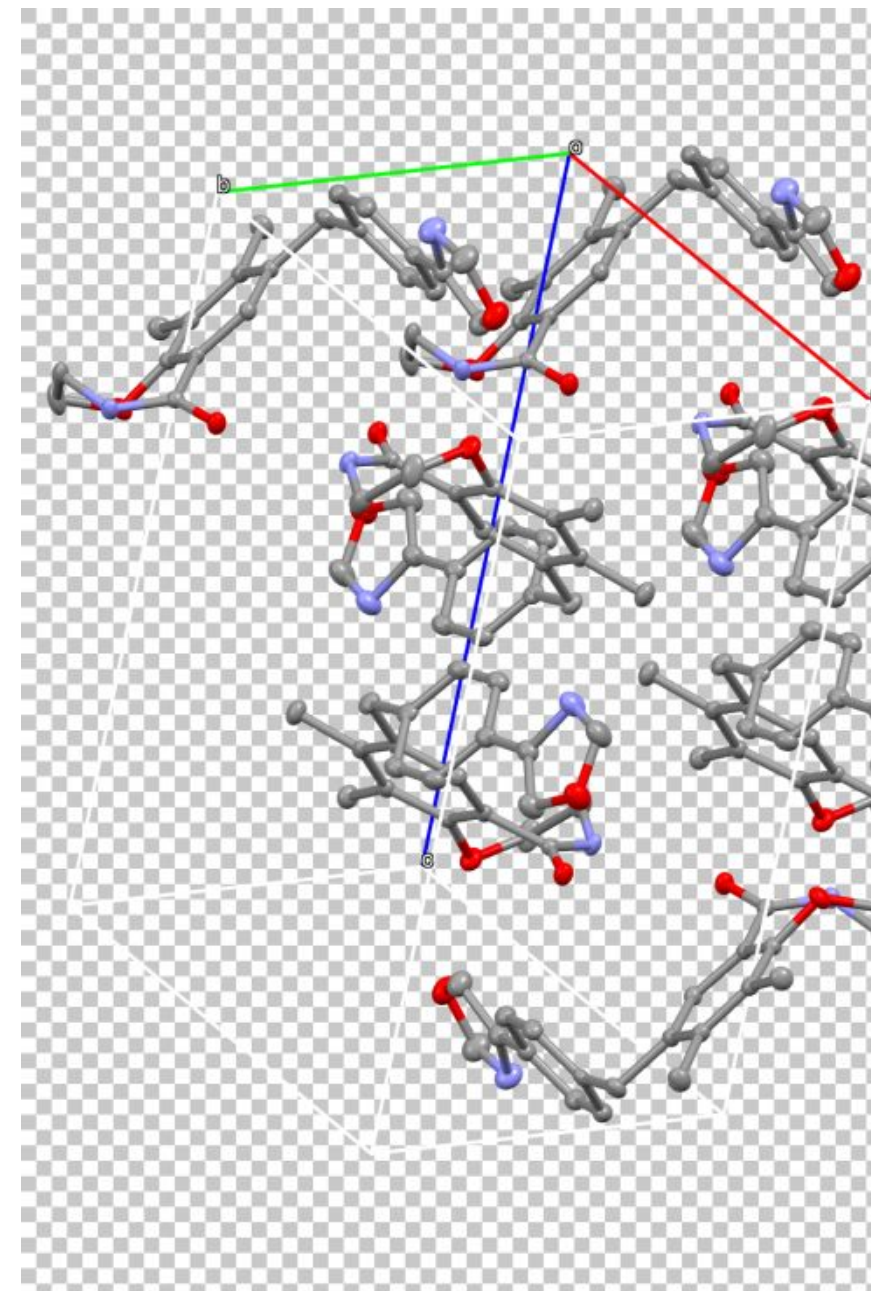

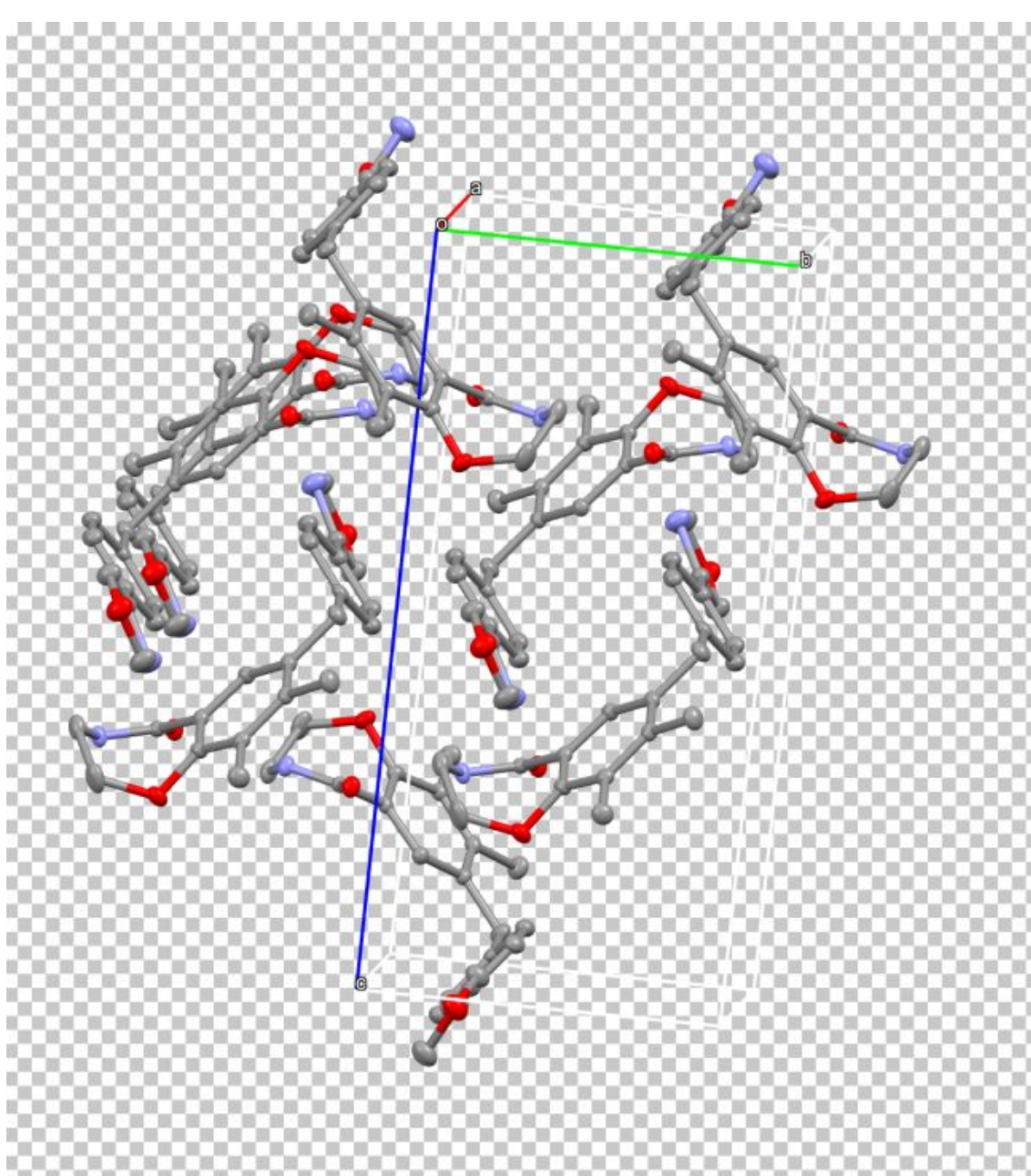

# Supplemental Figures

**IV PK and PO PK of VU6069863-02 in Rat**

| | |
|---|---|
| Project | M1 PAM |
| Study | IV PK and PO PK |
| Animals | Rat SD, male (n = 2) |
| Vehicle | (IV): 10% EtOH 40% PEG400 50% Saline<br>(PO): 10% Tween 80 |
| Dose | (IV) 1 mg/kg; (PO) 10 mg/kg |
| Matrix | EDTA Plasma |
| Analyte | VU6069863-02 |
| LLOQ | 0.5 ng/mL |

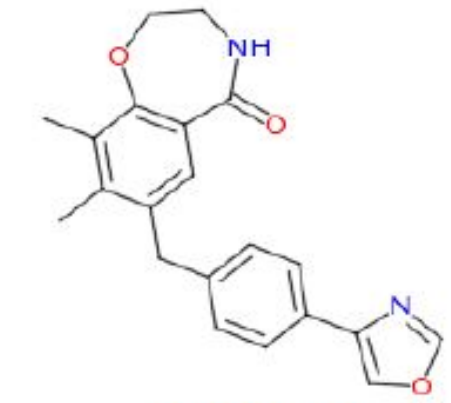


VU6069863-02

| 1 mg/kg IV VU6069863-02 | | | | |
|---|---|---|---|---|
| Dose (mg/kg) | Time (hr) | Concentration (ng/mL) | | |
| | | 1 | 2 | Ave |
| 1 | 0.0333 | 2057 | 1913 | 1985 |
| | 0.117 | 1902 | 1257 | 1579 |
| | 0.25 | 1349 | 1143 | 1246 |
| | 0.5 | 1365 | 624 | 994 |
| | 1 | 766 | 419 | 593 |
| | 2 | 366 | 195 | 281 |
| | 4 | 112 | 32.5 | 72.2 |
| | 7 | 13.4 | BLQ | 13.4 |
| | 24 | BLQ | BLQ | N/A |

| 10 mg/kg PO VU6069863-02 | | | | |
|---|---|---|---|---|
| Dose (mg/kg) | Time (hr) | Concentration (ng/mL) | | |
| | | 1 | 2 | Ave |
| 10 | 0.25 | 3604 | 4288 | 3946 |
| | 0.5 | 4441 | 5774 | 5108 |
| | 1 | 5432 | 8441 | 6936 |
| | 2 | 4358 | 5630 | 4994 |
| | 4 | 1403 | 2237 | 1820 |
| | 7 | 664 | 668 | 666 |
| | 24 | BLQ | BLQ | N/A |

| | PK Parameter | | 1 | 2 | Ave |
|---|---|---|---|---|---|
| IV PK | $t_{1/2\,(term)}$ | hr | 1.04 | 0.81 | 0.92 |
| | MRT | hr | 1.34 | 1.00 | 1.17 |
| | Cl_obs | mL/min/kg | 6.5 | 11.8 | 9.1 |
| | Vss | L/kg | 0.52 | 0.71 | 0.61 |
| | AUC (IV) | hr*ng/mL | 2576 | 1416 | 1996 |
| PO PK | Cmax | ng/mL | 5432 | 8441 | 6937 |
| | Tmax | hr | 1.00 | 1.00 | 1.00 |
| | AUC (PO) | hr*ng/mL | 19494 | 26181 | 22837 |
| | F | % | 98 | 131 | 114 |

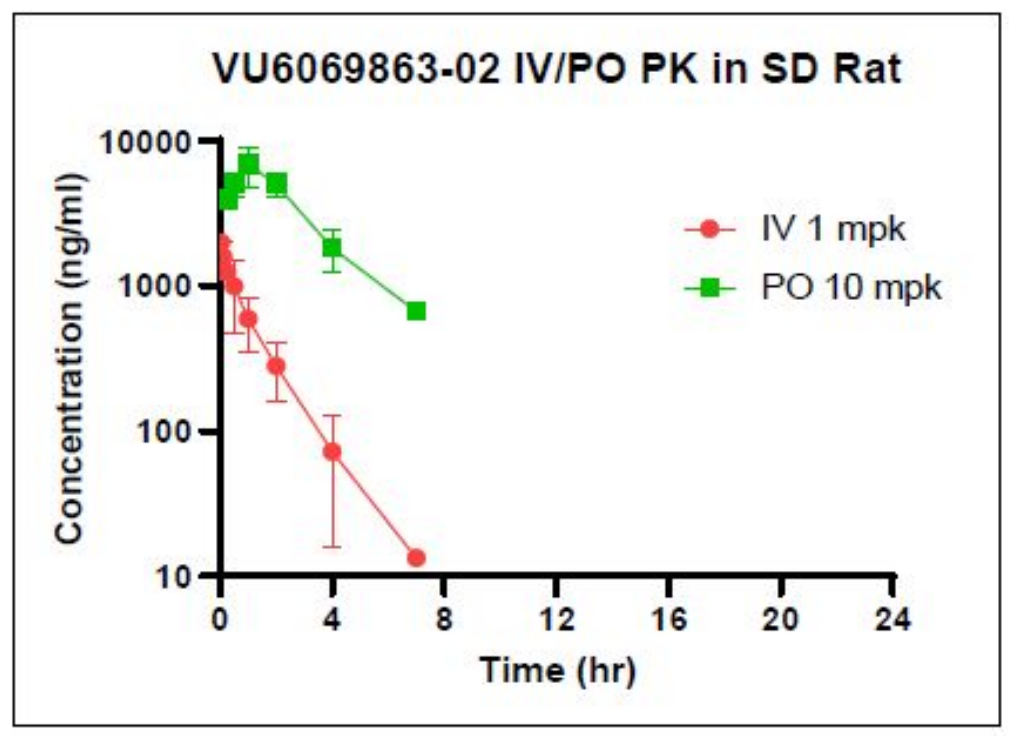


**Figure S1: Rat IV/PO PK of VU6069863 (11)**

| Compound I.D. | Client Compound I.D. | Test Concentration | %Control 1st | 2nd | 3rd | Mean %Control | Cytotoxicity (% of control) |
|---|---|---|---|---|---|---|---|
| **Bacterial cytotoxicity (TA98 - S9)** | | | | | | | |
| 100070986-1 | VU6069863-04 | 6.0E-07 M | 98.6 | 86.9 | 114.2 | **99.9** | 100 |
| 100070986-1 | VU6069863-04 | 1.2E-06 M | 107.9 | 97.8 | 95.5 | **100.4** | 100 |
| 100070986-1 | VU6069863-04 | 2.5E-06 M | 107.1 | 102.5 | 99.4 | **103.0** | 103 |
| 100070986-1 | VU6069863-04 | 5.0E-06 M | 111.0 | 88.4 | 92.3 | **97.3** | 97 |
| 100070986-1 | VU6069863-04 | 1.0E-05 M | 100.1 | 90.0 | 98.6 | **96.2** | 96 |
| 100070986-1 | VU6069863-04 | 2.5E-05 M | 96.2 | 104.0 | 97.8 | **99.4** | 99 |
| 100070986-1 | VU6069863-04 | 5.0E-05 M | 97.0 | 96.2 | 97.0 | **96.8** | 97 |
| 100070986-1 | VU6069863-04 | 1.0E-04 M | 97.8 | 102.5 | 101.7 | **100.6** | 101 |
| **Bacterial cytotoxicity (TA100 - S9)** | | | | | | | |
| 100070986-1 | VU6069863-04 | 6.0E-07 M | 96.9 | 87.7 | 92.7 | **92.4** | 92 |
| 100070986-1 | VU6069863-04 | 1.2E-06 M | 102.8 | 95.2 | 98.6 | **98.9** | 99 |
| 100070986-1 | VU6069863-04 | 2.5E-06 M | 111.2 | 106.2 | 103.6 | **107.0** | 107 |
| 100070986-1 | VU6069863-04 | 5.0E-06 M | 117.9 | 115.4 | 121.3 | **118.2** | 118 |
| 100070986-1 | VU6069863-04 | 1.0E-05 M | 123.8 | 113.7 | 129.7 | **122.4** | 122 |
| 100070986-1 | VU6069863-04 | 2.5E-05 M | 134.7 | 150.6 | 152.3 | **145.9** | 146 |
| 100070986-1 | VU6069863-04 | 5.0E-05 M | 140.6 | 155.7 | 139.7 | **145.3** | 145 |
| 100070986-1 | VU6069863-04 | 1.0E-04 M | 133.0 | 154.8 | 138.0 | **142.0** | 142 |
| **Bacterial cytotoxicity (TA1535 - S9)** | | | | | | | |
| 100070986-1 | VU6069863-04 | 6.0E-07 M | 81.6 | 95.2 | 87.1 | **88.0** | 88 |
| 100070986-1 | VU6069863-04 | 1.2E-06 M | 66.2 | 78.9 | 73.5 | **72.9** | 73 |
| 100070986-1 | VU6069863-04 | 2.5E-06 M | 77.1 | 82.5 | 78.9 | **79.5** | 80 |
| 100070986-1 | VU6069863-04 | 5.0E-06 M | 76.2 | 81.6 | 81.6 | **79.8** | 80 |
| 100070986-1 | VU6069863-04 | 1.0E-05 M | 80.7 | 83.4 | 83.4 | **82.5** | 83 |
| 100070986-1 | VU6069863-04 | 2.5E-05 M | 89.8 | 94.3 | 93.4 | **92.5** | 93 |
| 100070986-1 | VU6069863-04 | 5.0E-05 M | 91.6 | 91.6 | 94.3 | **92.5** | 93 |
| 100070986-1 | VU6069863-04 | 1.0E-04 M | 94.3 | 98.0 | 98.0 | **96.7** | 97 |
| **Bacterial cytotoxicity (TA1537 - S9)** | | | | | | | |
| 100070986-1 | VU6069863-04 | 6.0E-07 M | 79.9 | 105.3 | 119.4 | **101.5** | 102 |
| 100070986-1 | VU6069863-04 | 1.2E-06 M | 82.7 | 93.1 | 93.1 | **89.6** | 90 |
| 100070986-1 | VU6069863-04 | 2.5E-06 M | 82.7 | 88.4 | 98.7 | **89.9** | 90 |
| 100070986-1 | VU6069863-04 | 5.0E-06 M | 88.4 | 82.7 | 86.5 | **85.9** | 86 |
| 100070986-1 | VU6069863-04 | 1.0E-05 M | 94.0 | 84.6 | 88.4 | **89.0** | 89 |
| 100070986-1 | VU6069863-04 | 2.5E-05 M | 114.7 | 120.3 | 89.3 | **108.1** | 108 |
| 100070986-1 | VU6069863-04 | 5.0E-05 M | 95.9 | 106.2 | 106.2 | **102.8** | 103 |
| 100070986-1 | VU6069863-04 | 1.0E-04 M | 88.4 | 90.3 | 91.2 | **89.9** | 90 |

Notes:
1. Cytotoxicity is presented as % of control growth.
2. A cytotoxicity value of less than 60 % is flagged, and the compound is considered as toxic at the respective concentration.

**Figure S2: Genetic toxicity test (Ames) of VU6069863 (11) -** Eurofins Discovery Services Study US034-0023725

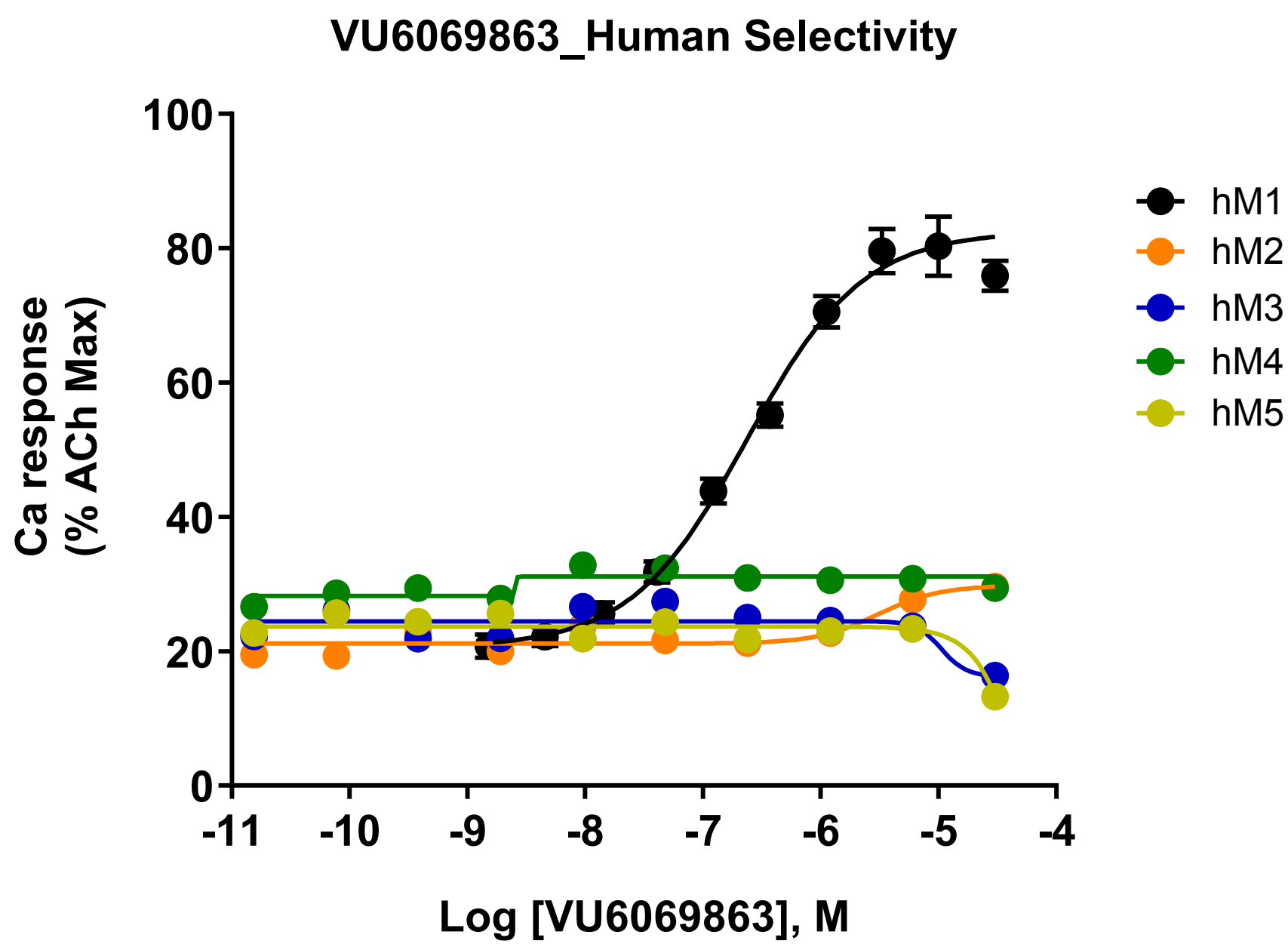


**Figure S3: human $M_2$-$M_5$ 11-point concentration response curves.**

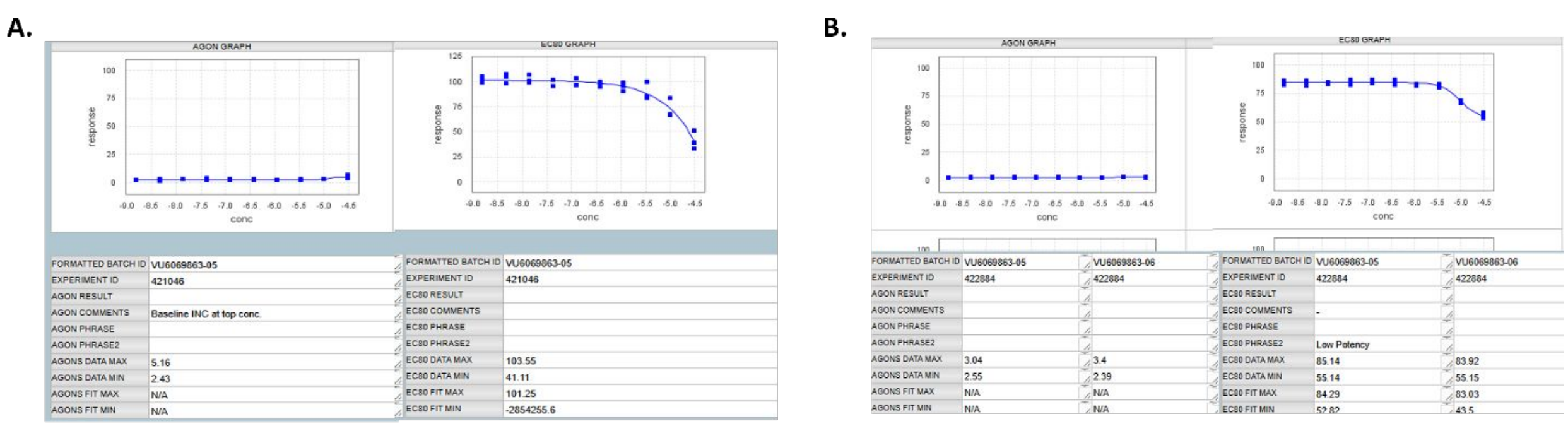


**Figure S4: A) 5-$HT_{2A}$ and B) 5-$HT_{2B}$ 10-point concentration response curves in agonist and antagonist mode.**

Left: acute phase (analysis between T25 and T30, 5 last min of the compound perfusion period)
Right: late phase (analysis between T75 and T80, 5 last min of the washout period)
Normalization to the baseline period (analysis between T0 and T10)

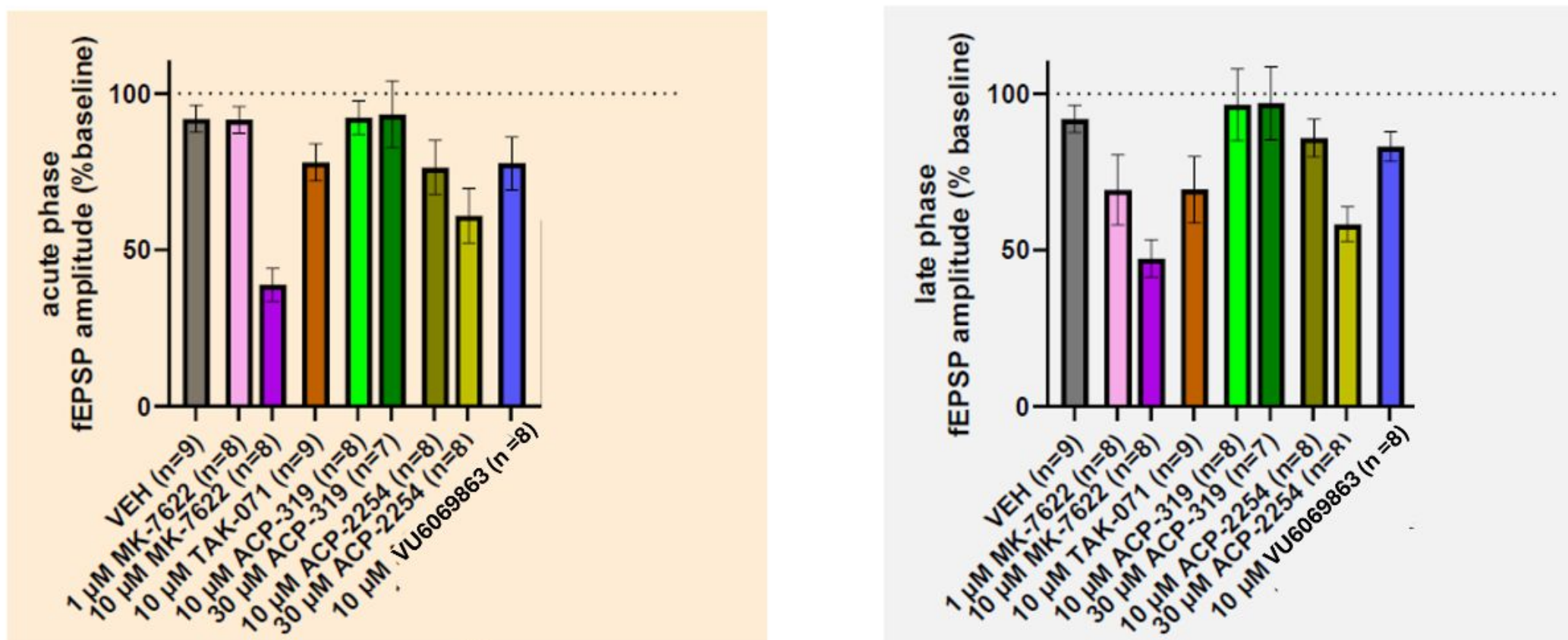


| Compounds | Concentration | % Baseline | PPR (%) | Mouse PAM EC50 | [LTD]/ mouse EC50 | Human PAM EC50 | 10,000/ human EC50 |
|---|---|---|---|---|---|---|---|
| Vehicle | -- | 91% | 102% | | | | |
| ACP-319 | 30 µM | 97% | 108% | 728 | 41 | 492 | 61 |
| ACP-319 | 10 µM | 92% | 113% | 728 | 14 | 492 | 20 |
| VU-2254 | 10 µM | 86% | 112% | 80 | 125 | 59 | 169 |
| VU-9863 | 10 µM | 83% | 115% | 945 | 11 | 248 | 40 |

**Figure S5: LTD electrophysiology studies.**

## References


1. Phelan, K.D., Shwe, U.T., Williams, D.K., Greenfield, L.J., Zheng, F. Pilocarpine-induced Status Epilepticus in Mice: A comparison of spectral analysis of electroencephalogram and behavioral grading using the Racine scale. *Epilepsy Research*, **2015**, *117*: 90-96.
2. Moran, S.P., Dickerson, J.W., Cho, H.P., Xiang, Z., Maksymetz, J., Remke, D.H., Lv, X., Doyle, C.A., Rajam, D.H., Niswender, C.M., Engers, D.W., Lindsley, D.W., Rook, J.M., Conn, P.J. $M_1$ positive allosteric modulators lacking agonist activity provide the optimal profile for enhancing cognition. *Neuropsychopharmacology*, **2018**, *43*, 1763-1771.
3. Rook, J.M., Bertron, J.L., Cho, H.P., Garcia-Barrantes, P.M., Moran, S.P., Maksymetz, J.T., Nance, K.D., Dickerson, J.W., Remke, D.H., Chang, S., Harp, J.M., Blobaum, A.L., Niswender, C.M., Jones, C.K., Stauffer, S.R., Conn, P.J., Lindsley, C.W. A Novel $M_1$ PAM VU0486846 Exerts Efficacy in Cognition Models without Displaying Agonist Activity or Cholinergic Toxicity. *ACS Chemical Neuroscience*, **2018**, *9*, 2274-2285.
